# Synthesis and Pharmacological Activities of Pyrazole Derivatives: A Review

**DOI:** 10.3390/molecules23010134

**Published:** 2018-01-12

**Authors:** Khalid Karrouchi, Smaail Radi, Youssef Ramli, Jamal Taoufik, Yahia N. Mabkhot, Faiz A. Al-aizari, M’hammed Ansar

**Affiliations:** 1 Medicinal Chemistry Laboratory, Faculty of Medicine and Pharmacy, Mohammed V University, 10100 Rabat, Morocco; Khalid.karrouchi@um5s.net.ma (K.K.); yramli76@yahoo.fr (Y.R.); jataoufik@hotmail.com (J.T.); ansarmhammed@gmail.com (M.A.); 2 LCAE, Department of Chemistry, Faculty of Sciences, University Mohamed I, 60000 Oujda, Morocco; 3 Physicochemical Service, Drugs Quality Control Laboratory, Division of Drugs and Pharmacy, Ministry of Health, 10100 Rabat, Morocco; 4 Department of Chemistry, Faculty of Science, King Saud University, P.O. Box 2455, Riyadh 11451, Saudi Arabia; faizalaizari@yahoo.com

**Keywords:** pyrazole derivatives, synthesis, biological activities

## Abstract

Pyrazole and its derivatives are considered a pharmacologically important active scaffold that possesses almost all types of pharmacological activities. The presence of this nucleus in pharmacological agents of diverse therapeutic categories such as celecoxib, a potent anti-inflammatory, the antipsychotic CDPPB, the anti-obesity drug rimonabant, difenamizole, an analgesic, betazole, a H2-receptor agonist and the antidepressant agent fezolamide have proved the pharmacological potential of the pyrazole moiety. Owing to this diversity in the biological field, this nucleus has attracted the attention of many researchers to study its skeleton chemically and biologically. This review highlights the different synthesis methods and the pharmacological properties of pyrazole derivatives. Studies on the synthesis and biological activity of pyrazole derivatives developed by many scientists around the globe are reported.

## 1. Introduction

Pyrazoles are five-membered heterocycles that constitute a class of compounds particularly useful in organic synthesis. They are one of the most studied groups of compounds among the azole family. Indeed, a huge variety of synthesis methods and synthetic analogues have been reported over the years.

The presence of the pyrazole nucleus in different structures leads to diversified applications in different areas such as technology, medicine and agriculture. In particular, they are described as inhibitors of protein glycation, antibacterial, antifungal, anticancer, antidepressant, antiinflammatory, anti-tuberculosis, antioxidant as well as antiviral agents [1,2].

**Figure 1:**
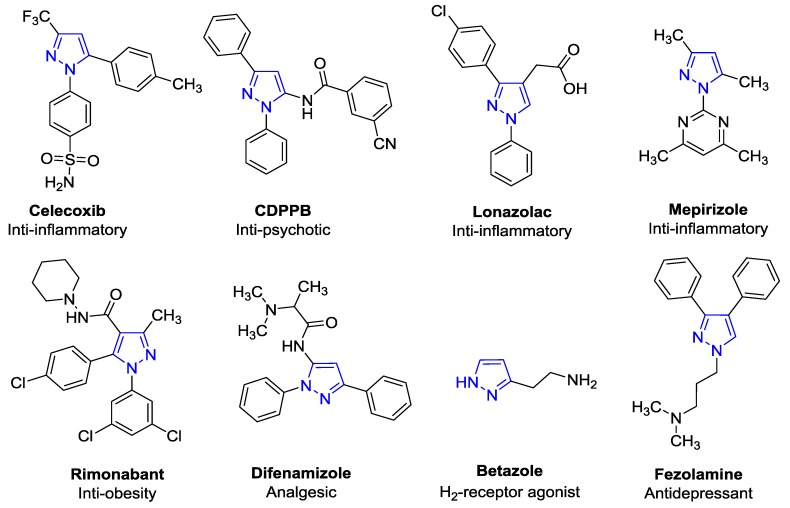
Pharmaceutical drugs containing pyrazole unit.

Nowadays, pyrazole systems, as biomolecules, have attracted more attention due to their interesting pharmacological properties. This heterocycle can be traced in a number of well-established drugs belonging to different categories with diverse therapeutic activities (Figure 1) [3,4,5,6,7,8,9,10].

In this review, we present descriptions and discussions on the most relevant synthesis methods and pharmacological properties of pyrazole-derived heterocyclic systems.

## 2. The Main Methods of Access to the Pyrazole Nucleus

**Figure 2:**
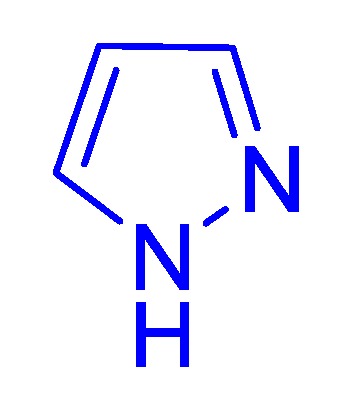
Structure of pyrazole.

Pyrazole is a π-excess aromatic heterocycle. Electrophilic substitution reactions occur preferentially at position 4 and nucleophilic attacks at positions 3 and 5 (Figure 2).

The pyrazoles diversely substituted by aromatic and heteroaromatic groups possess numerous biological activities, which makes them particularly interesting. The various access routes to the pyrazole nucleus have undergone numerous modifications since the first syntheses described by Knorr [11]. In this section, we will study this evolution and present the methods generally used to access substituted pyrazoles, that is to say:

- Cyclocondensation of hydrazine and similar derivatives with carbonyl systems.
- Dipolar cycloadditions.
- Multicomponent reactions.

### 2.1. Cyclocondensation of Hydrazine and Its Derivatives on 1,3-Difunctional Systems

**Figure 3:**
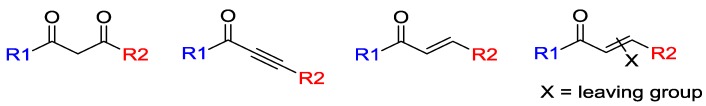
Examples of α,β-unsaturated carbonyl compounds.

The leading method used for obtaining substituted pyrazoles is a cyclocondensation reaction between an appropriate hydrazine acting as a bidentate nucleophile and a carbon unit like a 1,3-dicarbonyl compound, a 1,3-dicarbonyl derivatives or an α,β-unsaturated ketone (Figure 3).

#### 2.1.1. From 1,3-Diketones

**Scheme 1:**
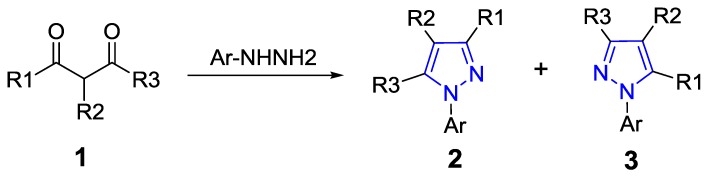
Synthesis of polysubstituted pyrazoles form 1,3-dicarbonyl compounds.

The cyclocondensation of the 1,3-dicarbonyl compounds with the hydrazine derivatives is a simple and rapid approach to obtain polysubstituted pyrazoles. The first synthesis of the substituted pyrazoles was carried out in 1883 by Knorr et al. [11] who reacted β-diketone **1** with hydrazine derivatives to give two regioisomers **2** and **3** (Scheme 1).

**Scheme 2:**
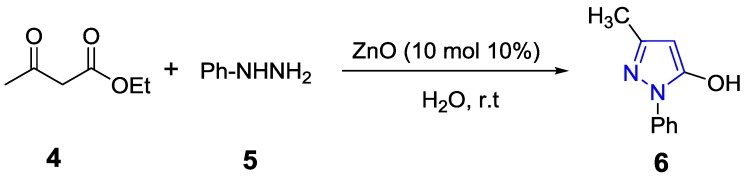
Synthesis of 1,3,5-substituted pyrazoles from ethyl acetoacetate.

Girish et al. [12] described an efficient nano-ZnO catalyzed green protocol for the synthesis of 1,3,5-substituted pyrazoles derivatives **6** by condensation of phenylhydrazine **5** with ethyl acetoacetate (**4**) (Scheme 2). The main advantage of this protocol is the excellent yield (95%) achieved, short reaction time and easy work-up procedure. 

**Scheme 3:**
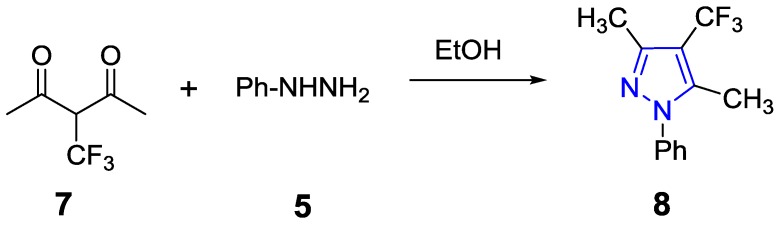
Synthesis of 1,3,4,5-substituted pyrazoles from 2-(trifluoromethyl)-1,3-diketone.

Similarly, Ohtsuka et al. [13] studied the condensation of phenylhydrazine **5** with the 2-(trifluoromethyl)-1,3-diketone **7** in ethanol, affording 1,3,4,5-substituted pyrazole **8** in good yield (63%). Compound **8** was exclusively formed presumably owing to the fact that the sterically small NH_2_ is more nucleophilic than NHPh (Scheme 3).

**Scheme 4:**
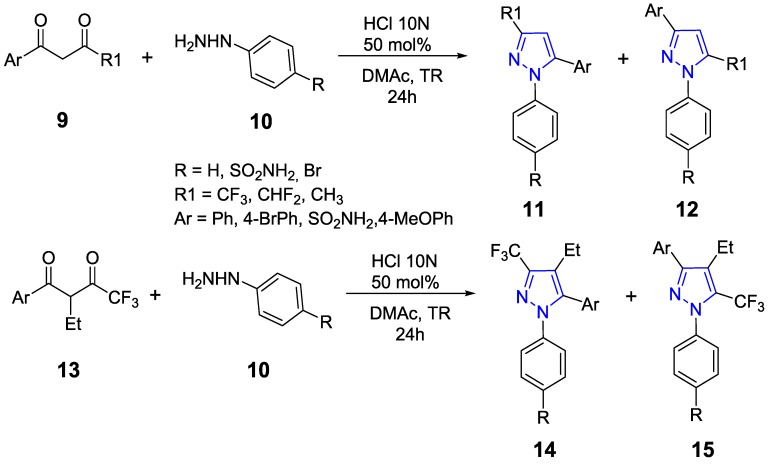
Synthesis of pyrazoles from 1,3-diketones and arylhydrazines.

Gosselin and co-workers have proposed new reaction conditions for the regioselective synthesis of 1,3-substituted 1-arylpyrazoles from 1,3-dicarbonyl compounds. Indeed, the authors have found that the cyclocondensation of an aryl hydrochloride hydrazine with 1,3-diketones in aprotic dipolar solvents gives better results than in the polar protic solvents (like ethanol) generally used for this type of reaction. After optimization of the conditions, the addition of a solution of HCl 10 N to the amide solvent (DMF, NMP, DMAc) or urea (DMPU, TMU) makes it possible to increase the yields by accelerating the dehydration steps. The cyclocondensation of the diketones with hydrazine thus takes place at ambient temperature in *N*,*N*-dimethylacetamide, in an acid medium, to give the corresponding pyrazoles with good yields and good regioselectivity. The condensation of various arylhydrazine with 4,4,4-trifluoro-1-arylbutan-1,3-diketones **9**, afforded two isomers **11**, **12** with 74–77% yields. The selectivity obtained is of the order of 98:2 in favor of the isomer **11**. By comparison, the reactions carried out under conventional conditions in ethanol, at ambient temperature, give equimolar mixtures of the regioisomers. Nevertheless, a loss of control of the regioselectivity is observed when the CF_3_ group is replaced by a CH_3_ or CHF_2_. Finally, the condensations of aryl hydrazines with the 1,3-diketones **13** that are 2-substituted by an alkyl group give the trisubstituted pyrazoles **14** and **15** in 79–89% yields and a regioselectivity greater than 99.8:0.2 in favor of isomer **15** in all cases (Scheme 4) [14].

#### 2.1.2. From Acetylenic Ketones

**Scheme 5:**
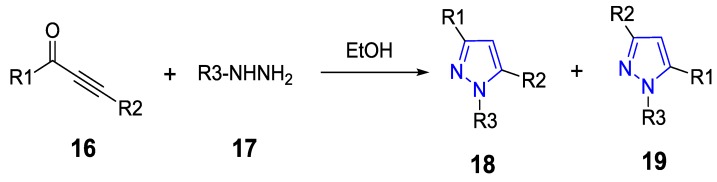
Synthesis of pyrazoles from acetylenic ketones.

The cyclocondensation reaction of hydrazine derivatives **17** on acetylenic ketones **16** to form pyrazoles has been known for more than 100 years [15]. However, the reaction again results in a mixture of two regioisomers **18** and **19** (Scheme 5).

**Scheme 6:**
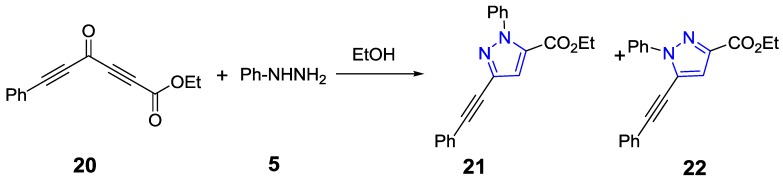
Synthesis of pyrazoles from diacetylene ketones.

The diacetylene ketones **20** reacted with phenylhydrazine **5** in ethanol to give two regioisomeric pyrazoles **21** and **22**. When phenylhydrazine was used, a mixture of regio-isomers **21**/**22** was generated in approximately 3:2 ratio. When hydrazine hydrate was used as the nucleophile, only regioisomer **21** was isolated, presumably due to hydrogen bonding to the ethyl ester group (Scheme 6) [16].

**Scheme 7:**
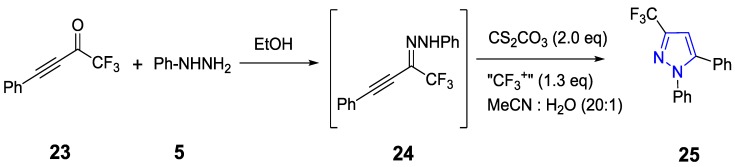
Synthesis of 3-trifluoromethylpyrazoles via cyclization of acetylenic ketones.

Guojing et al. reported a new efficient method for the synthesis of 3-trifluoromethylpyrazoles **25** with good yields via trifluoromethylation/cyclization of acetylenic ketones **25** on phenylhydrazine **5** using a hypervalent iodine reagent under transition-metal-free conditions. The optimal conditions were obtained when the ratio **24**/Togni reagent was maintained at 1:1.3, giving **25** in 70% isolated yield (Scheme 7) [17].

**Scheme 8:**
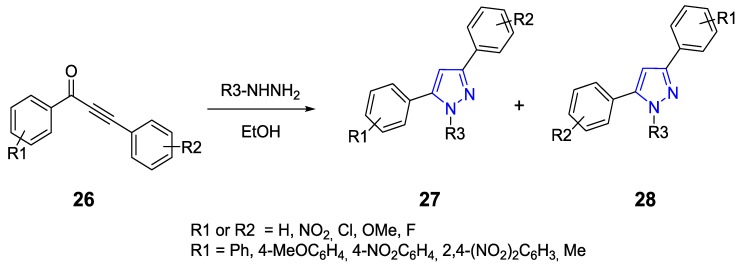
Synthesis of 3,5-diarylpyrazoles from acetylenic ketones and hydrazines derivatives.

Bishop et al. were interested in the factors determining the regioselectivity of this type of reaction in the framework of the synthesis of 3,5-diarylpyrazoles. They studied the cyclocondensation of acetylenic ketones **26** on methylhydrazine or aryl hydrazines in ethanol, which provides two difficultly separable regioisomeric pyrazoles **27** and **28** (Scheme 8) [18]. 

The difference in regioselectivity observed when using methylhydrazine (ratio **27**/**28** = 93:3 to 97:3) or an arylic hydrazine (ratio **28**/**27** = 87:13 to 99:1) is explained by the fact that the nitrogen carrying a methyl group is much more nucleophilic and will react by Michael addition on the triple bond of the acetylenic ketone followed by the intramolecular formation of an imine. In the case of a hydrazine substituted by an aryl group, the primary amine is the most nucleophilic and will react on the triple bond followed by the attack of the secondary amine on the carbonyl.

#### 2.1.3. From Vinyl Ketones

**Scheme 9:**
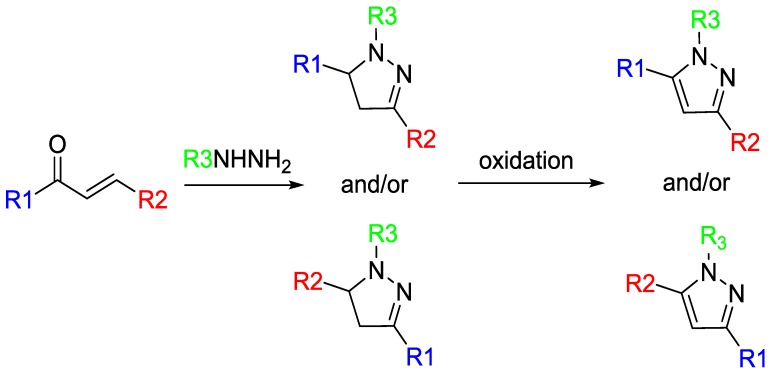
Synthesis of pyrazoles by cyclocondensation reaction of α,β-ethylenic ketone.

The cyclocondensation reaction between an α,β-ethylenic ketone and a hydrazine derivative results in the synthesis of pyrazolines which, after oxidation, provide the pyrazole ring (Scheme 9).

**Scheme 10:**
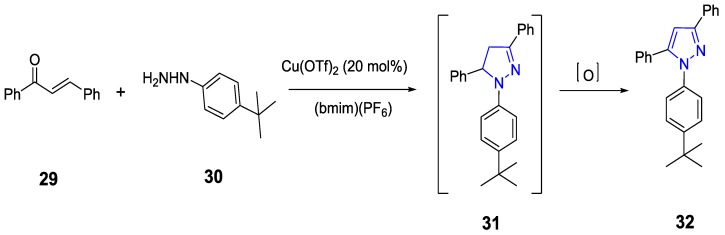
Synthesis of pyrazoles from α,β-ethylenic ketone.

Rao et al. described the condensation of an α,β-ethylenic ketone **29** with *p*-(4-(*tert*-butyl)phenyl)hydrazine **30** in the presence of copper triflate and 1-butyl-3-methylimidazolium hexafluorophosphate [bmim] (PF6) as catalysts, to access pyrazoline **31**. The corresponding 1,3,5-trisubstituted pyrazole **32** was obtained after oxidation in situ of this pyrazoline. The reaction protocol gave 1,3,5-triarylpyrazoles in good yields (about 82%) via a one-pot addition–cyclocondensation between chalcones and arylhydrazines, and oxidative aromatization stands without the requirement of an additional oxidizing reagent. The catalyst can be reused more than four cycles without much loss in the catalytic activity (Scheme 10) [19].

**Scheme 11:**
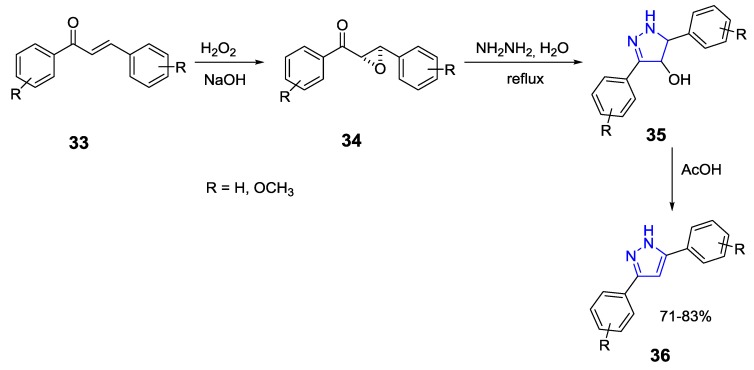
Synthesis of 3,5-diaryl-*1H*-pyrazoles from β-arylchalcones.

On the other hand, Bhat et al. described a method for the synthesis of 3,5-diaryl-1*H*-pyrazoles from the reaction β-arylchalcones **33** with hydrogen peroxide that gave epoxides **34**. Then, addition of hydrazine hydrate afforded pyrazoline intermediates **35**, dehydration of which yielded desired 3,5-diaryl-1*H*-pyrazoles **36** (Scheme 11) [20].

**Scheme 12:**
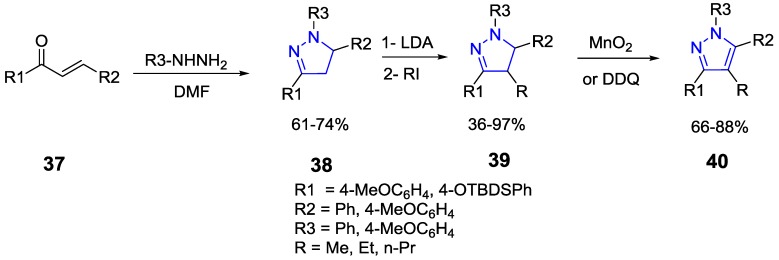
Synthesis of 4-alkyl-1,3,5-triarylpyrazoles from α,β-ethylenic ketones.

Huang et al. developed a new regioselective synthesis of 4-alkyl-1,3,5-triarylpyrazoles for the preparation of unsymmetrically substituted systems of interest as ligands for the estrogen receptor. The condensation of hydrazines with α,β-ethylenic ketones **37** in DMF gave pyrazolines **38**. However, the corresponding pyrazole derivatives **40** were obtained in good yield (66–88%) by alkylation of the pyrazolines **38** in the presence of LDA, before undergoing the oxidation reaction (Scheme 12) [21].

**Scheme 13:**
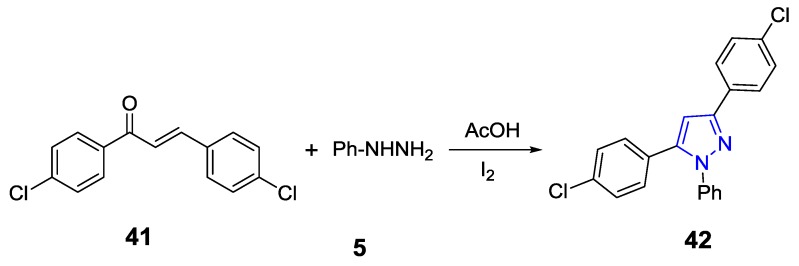
Synthesis of pyrazoles by cyclocondensation reaction of the α,β-ethylenic ketone.

Similarly, a method for the synthesis of 1,3,5-trisubstituted pyrazoles from an α,β-ethylenic ketone was described. Cyclocondensation of the α,β-ethylenic ketone **41** with phenylhydrazine (1.2 eq.) **5** in acetic acid and in the presence of iodine (1.0 eq.) afforted the corresponding pyrazole **42** in good yield (70%) (Scheme 13) [22]. 

#### 2.1.4. From Vinyl Ketones Having a Leaving Group

**Scheme 14:**
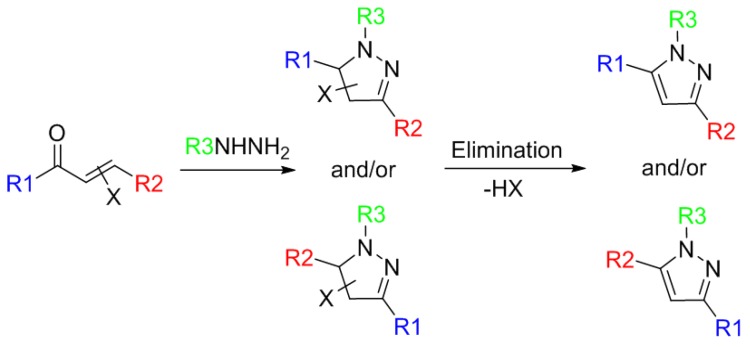
Synthesis of pyrazoles via cyclocondensation of α,β-ethylenic ketones having a leaving group.

The α,β-ethylenic ketones having a leaving group may react with hydrazine derivatives to form pyrazolines which, after removal of the leaving group, provide the desired pyrazoles (Scheme 14).

**Scheme 15:**
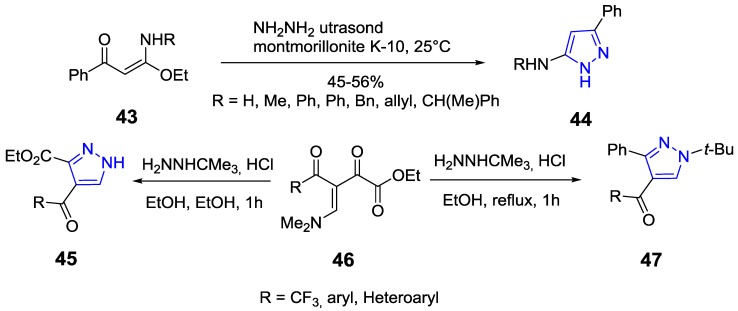
Synthesis of pyrazoles from α-oxoketene *O*,*N*-acetals and enaminodicketones.

5-Amino-3-phenylpyrazoles **44** were prepared from α-oxoketene *O*,*N*-acetals **43** using montmorillonite K-10 under sonication conditions [23]. The cyclocondensation of non-symmetrical enaminodicketones **46** on various hydrazine derivatives has been studied in the case of *tert*-butylhydrazine and carboxymethylhydrazine. The various pyrazoles **45** and **47** are obtained regiospecifically and in good yields (74–94%). It should be noted that in the case of carboxymethylhydrazine, the reaction leads directly to the corresponding NH-pyrazoles [24] (Scheme 15).

**Scheme 16:**
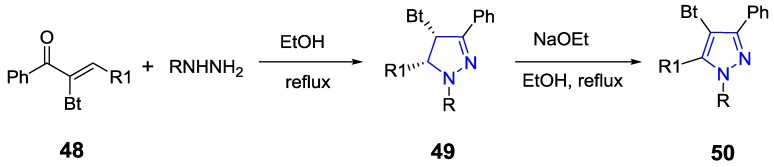
Synthesis of pyrazoles starting from α-benzotriazolylenones.

Katritzky et al. [25] described the synthesis of 1-methyl(aryl)-3-phenyl-5-alkyl(aryl)pyrazoles **50** by a regioselective condensation reaction of α-benzotriazolylenones **48** with methyl and phenylhydrazines. The intermediate pyrazolines **49** are then treated in a basic medium to give the expected pyrazoles in 50–94% yields after removal of benzotriazole. The advantage of using the benzotriazole group lies in the fact that the proton in the α-position is made more acidic and thus permits functionalization in the 4-position of the pyrazoline nucleus, thus allowing access to tetrasubstituted pyrazoles **50** (Scheme 16).

**Scheme 17:**
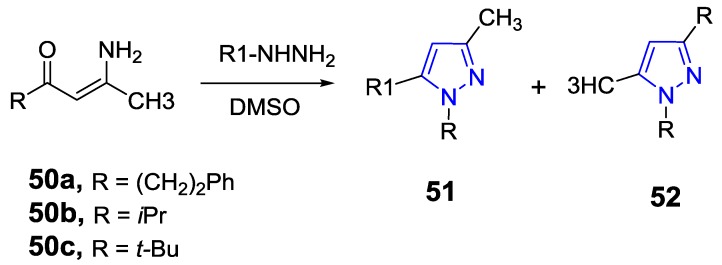
Synthesis of pyrazoles from β-aminoenones.

Alberola et al. [26] studied the regioselectivity of the reaction of various β-aminoenones on different monoalkyl, acetyl-, methoxycarbonyl-hydrazine and semicarbazide. Indeed, when the least bulky substituent is attached at the β position of the enone, high regioselectivity is obtained. This is the case of pyrazoles **51** and **52** which have been obtained with a regioselectivity greater than 90% from the reaction of alkylhydrazines (R1 = Me, t-Bu), in DMSO, with β-aminoenones **50a**, **50b** and **50c** which possess the smallest group (CH_3_) in the β position (Scheme 17 and Table 1).

**Scheme 18:**
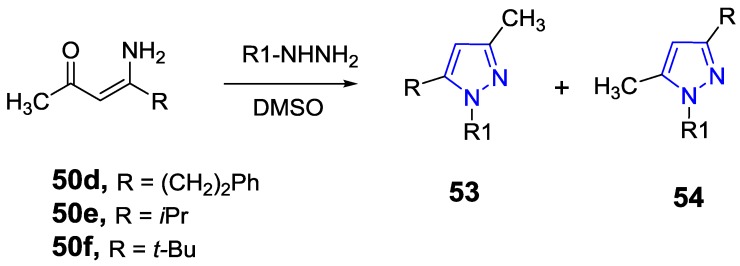
Synthesis of pyrazoles from β-aminoenones.

In the inverse case where the substituent in position β is the largest, the regioselectivity decreases. Indeed, when β-aminoenones **50a**, **50b** and **50c** were replaced by their position isomers **50d**, **50e** and **50f** in the previous reactions, a drop in regioselectivity was observed (Scheme 18 and Table 2).

This phenomenon is all the more important as R and alkylhydrazine are more voluminous. In this context, in order to increase the regioselectivity (to increase the ratio **54**/**53**) several experimental attempts have been carried out and have suggested that the selectivity would be improved if the reactions are catalyzed by acetic acid and carried out in DMSO or in ethanol.

### 2.2. The 1,3-Dipolar Cycloaddition

Other methods allowing access to the pyrazole nucleus involve [3 + 2] cyloaddition reactions between an alkyne (or an olefin) and 1,3-dipolar compounds such as the diazo compounds, the sydnones or the nitrilimines.

#### 2.2.1. Cycloaddition of Diazocarbonyl Compounds

**Scheme 19:**
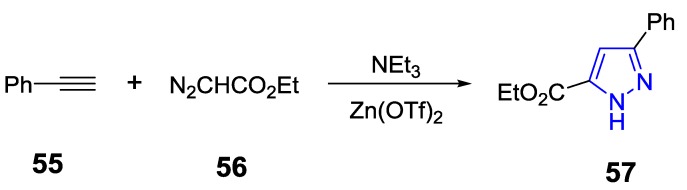
Synthesis of pyrazoles by 1,3-dipolar cycloaddition of ethyl α-diazoacetate.

He et al. [27] investigated the action of ethyl α-diazoacetate **56** on phenylpropargyl **55** in triethylamine and in the presence of zinc triflate as a catalyst; the 1,3-dipolar cycloaddition reaction, leads to the corresponding pyrazole **57** in good yield (89%). The simple reaction conditions, straightforward procedure, synthetically useful products, good yielding, and easy manipulation make this method potentially useful in organic synthesis (Scheme 19).

**Scheme 20:**
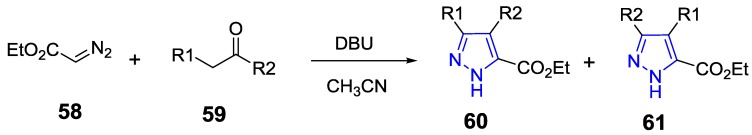
Synthesis of pyrazole-5-carboxylates using 1,3-dipolar cycloaddition of ethyl diazoacetate and α-methylene carbonyl.

Gioiello and co-workers described a facile one-pot procedure for the synthesis of pyrazole-5-carboxylates by 1,3-dipolar cycloaddition of ethyl diazoacetate **58** with α-methylene carbonyl **59** compounds utilizing 1,8-diazabicyclo[5.4.0]undec-7-ene (DBU) as base and acetonitrile as solvent. Pyrazoles **60** and **61** were obtained with excellent regioselectivity and good yields. In particular, when **58** was reacted with **59** (with R1 = R2 = Ph) in the presence of 1.7 eq. of DBU in acetonitrile under argon atmosphere at room temperature, ethyl 3,4-diphenyl-1*H*-pyrazole-5-carboxylate was obtained in 65% yield after flash chromatography. The reaction was found to proceed by a domino 1,3-dipolar cycloaddition water elimination (Scheme 20) [28].

**Scheme 21:**
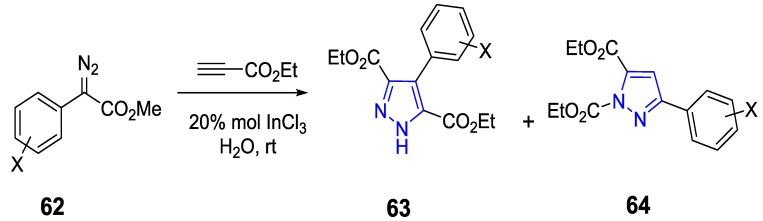
Synthesis of pyrazoles using 1,3-dipolar cycloaddition of α-diazoarylacetacetates.

Under the same conditions, the reaction of various aryl α-diazoarylacetacetates on methyl propionate led to the formation of two regioisomers **63** and 64. After cyclization, minor compound **64** (4–12%) is obtained by migration of the ester group on nitrogen atom. The majority compound **63** (77–90%) would be obtained by migration of the aryl group to the adjacent carbon atom followed by a prototropic rearrangement (Scheme 21) [29].

**Scheme 22:**
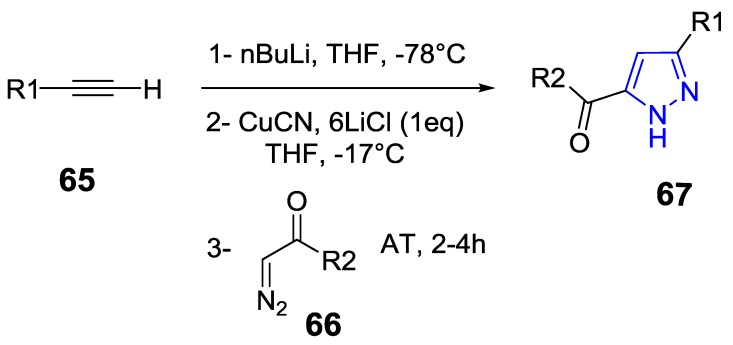
Synthesis of 3-acylpyrazoles using 1,3-dipolar cycloaddition of diazocarbonyl and acetylides.

In another approach, Qi and Ready developed a direct and efficient access towards 3-acylpyrazoles **67** that involves the copper-promoted cycloaddition of acetylides **65** with diazocarbonyl compounds **66** under mild conditions. A wide variety of substituents is tolerated at both the acetylide and the diazo compound. The method is a rare example of an inverse-electron-demand cycloaddition (Scheme 22) [30]. 

#### 2.2.2. The Sydnones

**Scheme 23:**
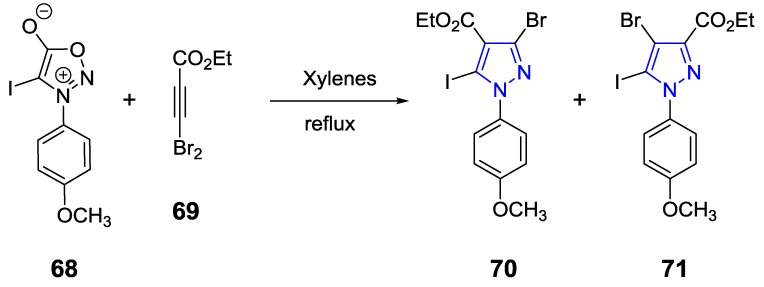
Synthesis of pyrazoles by cycloaddition reaction of sydnones and alkyne.

The pyrazoles ca obtained by a cycloaddition reaction of sydnones. Delaunay and co-workers presented the synthesis of the two regioisomeric 1,3,4,5-substituted pyrazoles **70** and **71** via a cycloaddition reaction between a sydnone **68** and alkyne 69. The reaction was completed within 15 h, giving rise to a 3:1 mixture of regioisomeric 5-iodopyrazoles **70** and **71** in 84% combined yield. The pyrazoles were easily separated by silica gel chromatography, and the structure assignment of the desired major isomer **70** (63% isolated yield) was made on the basis of the ^1^H-NMR spectrum [31] (Scheme 23).

**Scheme 24:**
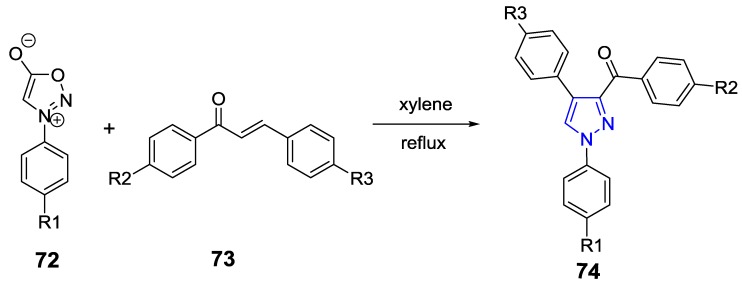
Synthesis of pyrazoles by 1,3-dipolar cycloaddition of arylsydnones and chalcone.

On the other hand, Chen et al. [32] described the synthesis of a trisubstituted pyrazole **74**, by 1,3-dipolar cycloaddition of arylsydnones **72** and α,β-unsaturated ketone **73** in dry xylene (Scheme 24).

#### 2.2.3. Nitrilimines

**Scheme 25:**
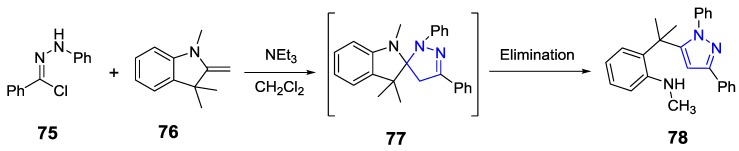
Synthesis of pyrazole by 1,3-dipolar cycloaddition of diphenylnitrilimine and alkene.

Dadiboyena et al. described the synthesis of 1,3,5-trisubstituted pyrazole **78** by 1,3-dipolar cycloaddition of diphenylnitrilimine **75** with an alkene **76** in dichloromethane in the presence of triethylamine. The trisubstituted pyrazole **78**, isolated in 88% yield, was the only product rather than the expected spiro-pyrazoline **77** (Scheme 25) [33]. 

**Scheme 26:**
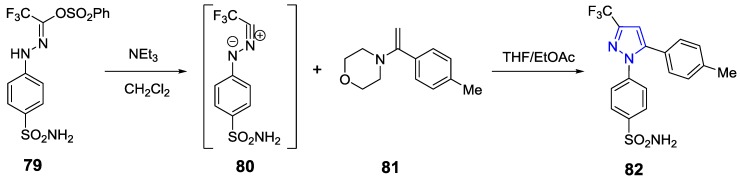
Synthesis of pyrazole via 1,3-dipolar cycloaddition reaction of nitrilimine and vinyl.

Oh et al. reported the synthesis of the 1,3,5-substituted pyrazole **82** via 1,3-dipolar cycloaddition reaction of a vinyl derivative **81** with the nitrilimine **80** generated in situ from an arylhydrazone **79**. The reaction yielded the corresponding pyrazole in 72% yield. The protocol is simple and practical, employing economical and readily available reagents (Scheme 26) [34].

### 2.3. Multicomponent Approaches

#### 2.3.1. In Situ Formation of Carbonyl Derivatives

**Scheme 27:**
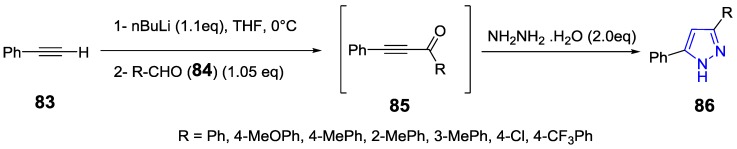
Synthesis of pyrazole from α,β-unsaturated carbonyl and hydrazine.

Harigae and co-workers reported the preparation of 3,5-substituted pyrazole **86** in good yields (68–99%) with high regioselectivity in one pot by the treatment of terminal alkynes **83** with aromatic aldehydes **84**, molecular iodine, and hydrazines. The present reaction is a simple and practical method for the preparation of various 1,3-disubstituted pyrazoles from easily available compounds (Scheme 27) [35].

**Scheme 28:**
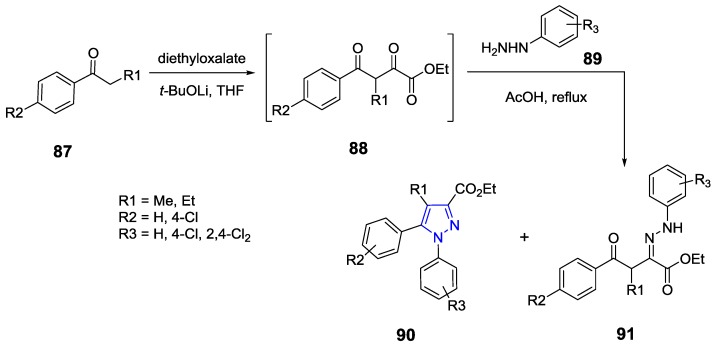
Synthesis of pyrazole via cyclocondensation reaction of 1,3-dicarbonyl and arylhydrazine.

1,3,4,5-Substituted pyrazole **90** was synthetized via a cyclocondensation reaction of arylhydrazine **89** and carbonyl derivatives **88** generated in situ from a ketone **87** and diethyl oxalate. The diketoesters **88** was converted into the desired 1,5-isomers **90** in the yields of 60–66%. Meanwhile, *N*-arylhydrazones **91** were obtained in the yields of 24–31% (Scheme 28) [36].

**Scheme 29:**
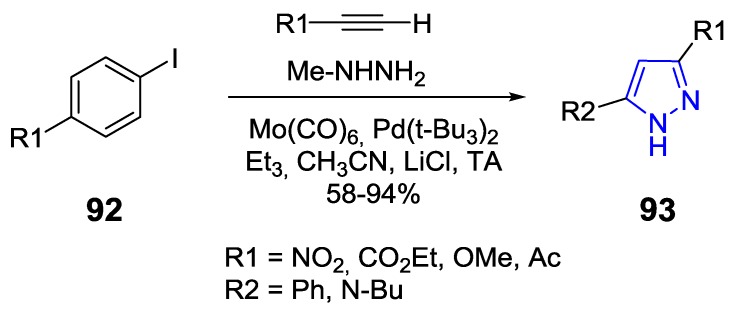
Synthesis of pyrazoles by one-pot reaction of aryl iodides, acetylenic acids and methylhydrazine.

Lizuka and Kondo demonstrate palladocatalyzed carbonylation of acetylenic acids on aryl iodides **92** in the presence of hexacarbonyl molybdenum as a source of CO and tri-*tert*-butylphosphine as a palladium ligand, to similarly access 1,3,5-substituted pyrazoles **93** with excellent yields (58–94%). The one-pot formation of pyrazole in the presence of methylhydrazine was also successful and aryl iodides with electron withdrawing groups were easily converted into pyrazoles (Scheme 29) [37]. 

#### 2.3.2. In Situ Formation of β-Aminoenones

**Scheme 30:**
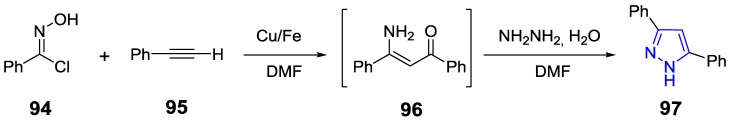
Synthesis of 3,5-diphenylpyrazoles via cuprocatalyzed coupling between alkyne and oxime.

Kovacs et al. developed a novel process for the synthesis of 3,5-substituted pyrazoles **97** via cuprocatalyzed coupling between an alkyne **95** and an oxime **94** in dimethylformamide, which provides the β-aminoenone **96**. The valuable β-aminoenone was transformed into pyrazoles with the addition of hydrazine in a straightforward one-pot procedure; the product **97** was isolated with 70% yield (Scheme 30) [38].

#### 2.3.3. In Situ Formation of a Hydrazone

**Scheme 31:**
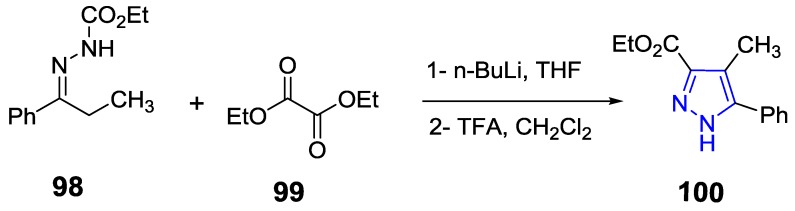
Synthesis of pyrazoles by one-pot cyclization of hydrazone with diethyl dioxalate.

Dang et al. described a novel reaction for the synthesis of pyrazole-3-carboxylates by one-pot cyclization of hydrazone dianions **98** with diethyl dioxalate **99**. The cyclization of diethyl oxalate with the dianions of hydrazones **98** afforded the pyrazole-3-carboxylates **100** in good yields (53%) (Scheme 31) [39].

**Scheme 32:**
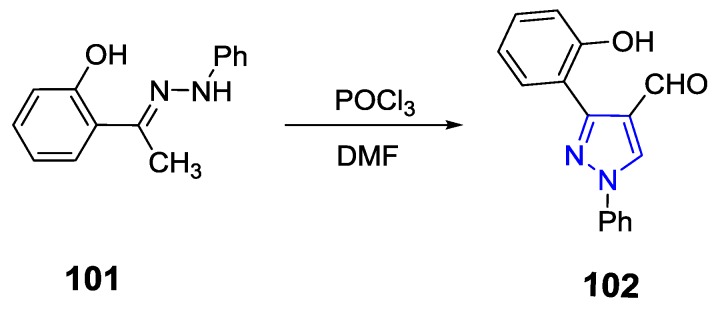
Synthesis of carboxaldehyde pyrazoles by Vilsmeier-Haack reaction.

Lokhande et al. used the Vilsmeier-Haack reaction to synthesize carboxaldehyde pyrazoles **101**. Condensation of a hydrazine **102** in the presence of phosphorus oxychloride gives the 4-formyl pyrazole (Scheme 32) [40].

**Scheme 33:**
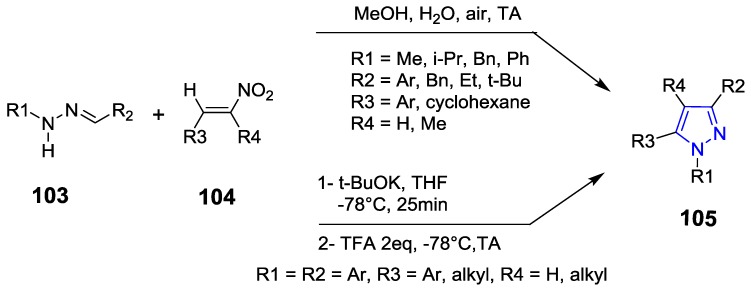
Synthesis of 1,3,4,5-Substituted pyrazole derivatives.

1,3,4,5-Substituted pyrazoles **105** were regioselectively synthesized in modest to good yields (26–92%) by Deng and Mani from hydrazone **103** and a nitroolefin **104** in methanol [41]. As a result of this work, the same authors proposed another strategy for synthesis based on the use of the same substrates and selectively leading to 1,3,4-substituted pyrazoles. In this new approach, the cyclocondensation of nitroolefins **104** with hydrazones **103** is carried out using a strong base such as *t*-BuOK. After treatment with a strong acid, the desired pyrazole **105** is obtained in the form of a single regioisomer in excellent yields (42–88%) (Scheme 33) [42]. 

#### 2.3.4. In Situ Formation of Diazo Compounds

**Scheme 34:**
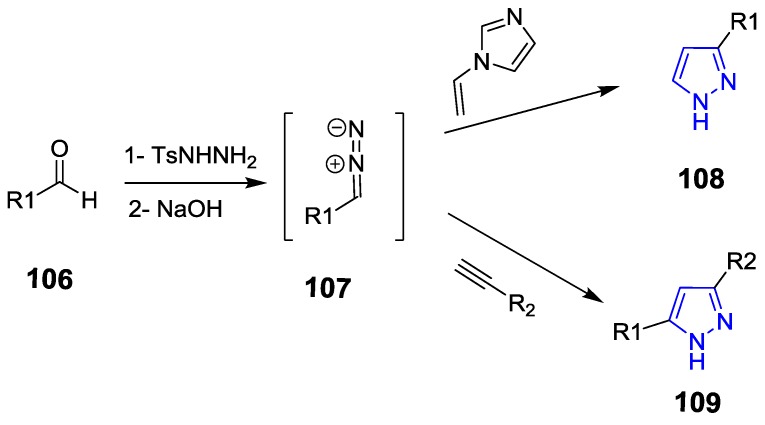
Synthesis of pyrazoles by 1,3-dipolar cycloaddition of diazo derivatives.

The Aggarwal team has developed a multicomponent process in which diazo **107** derivatives are generated in situ from various aldehydes **106** and tosylhydrazines, thus limiting the risks associated with the isolation of these compounds. These are then used in a 1,3-dipolar cycloaddition reaction to give corresponding pyrazoles **108** and **109** Diazo compounds derived from aldehydes were reacted with terminal alkynes to furnish regioselectively 3,5-disubstituted pyrazoles in 24–67% yields. Furthermore, the reaction of *N*-vinylimidazole and diazo compounds derived from aldehydes gave exclusively 3-substituted pyrazoles in a one-pot process with 32–79% yields (Scheme 34) [43].

### 2.4. From Heterocyclic Systems

#### 2.4.1. From the Pyranones

**Scheme 35:**
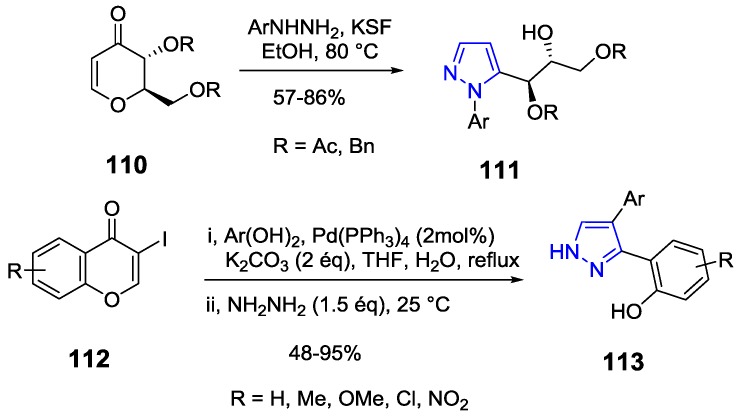
Synthesis of pyrazoles from pyranones.

Pyranones are among the most widely used heterocycles for the preparation of pyrazoles. The condensation of 2,3-dihydro-4*H*-pyran-4-ones **110** with arylhydrazines in ethanol and in the presence of montmorillonite KSF as catalyst, gives access to 5-substituted pyrazoles **111** in yields of (57–86%) (Scheme 35) [44]. Similarly, Xie et al. have developed a general method for the synthesis of pyrazoles. This involves the use of Suzuki coupling of arylboronic acids with chromones **112**, followed by the action of hydrazine hydrate, yields the corresponding 3,4-diarylpyrazoles **113** in a yield of 48–95% (Scheme 35) [45].

**Scheme 36:**
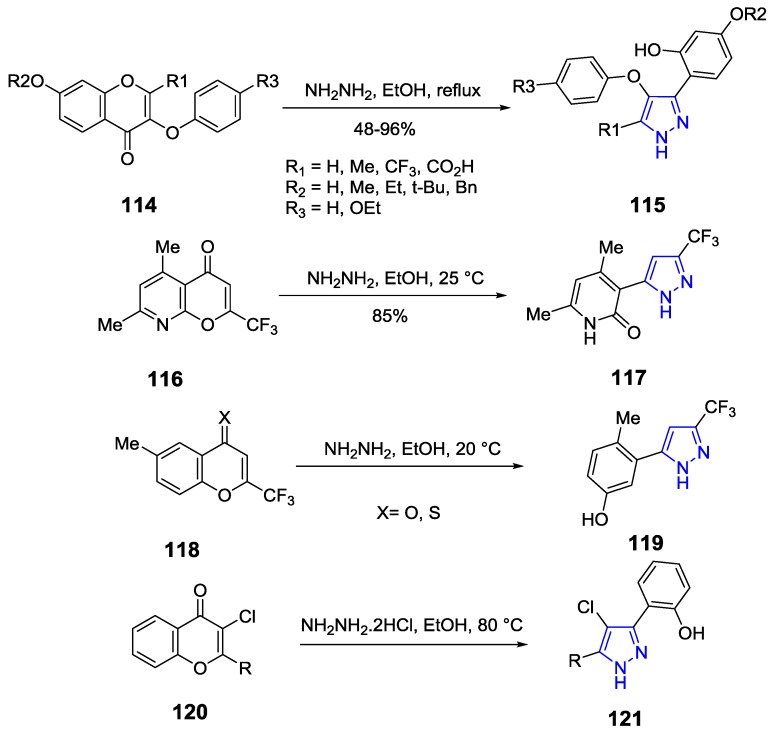
Synthesis of pyrazoles by reaction of pyranones derivatives with hydrazine.

Similarly, reaction of pyranones derivatives **114**, **116**, **118** and **120** with hydrazine in ethanol the give corresponding pyrazoles **115**, **117**, **119** and **121** respectively (Scheme 36) [46,47,48,49].

#### 2.4.2. From Furandiones

**Scheme 37:**
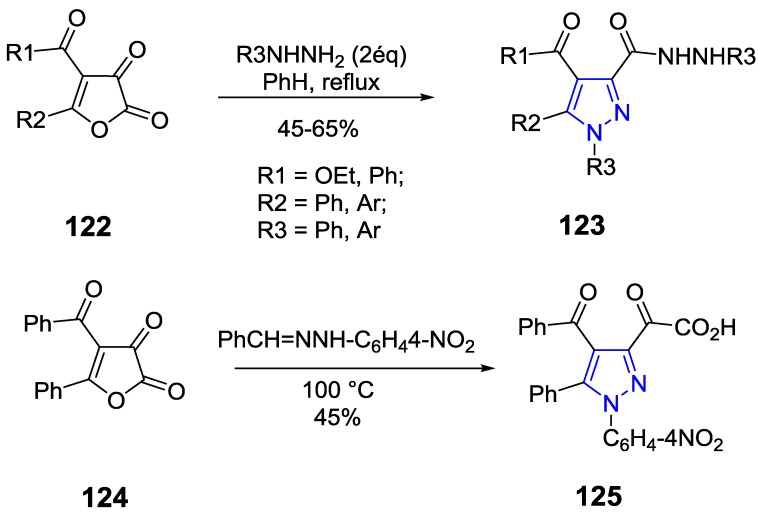
Synthesis of pyrazoles from furandiones.

Ilham et al. [50] carried out condensation in refluxing benzene, furan-2,3-diones **126** with arylhydrazines, allowing access to pyrazole-3-hydrazides **127** in acceptable to good yields (45–65%) (Scheme 37). Similarly, condensation of furan-2,3-dione **128** with *N*-benzylidene-*N*′-(4-nitrophenyl) hydrazine afforted 4-benzoyl-1-(4-nitrophenyl)-5-phenyl-1*H*-pyrazole-3-carboxylic acid **129** in a 45% yield (Scheme 37) [51].

#### 2.4.3. From Pyrimidines and Pyrimidones

**Scheme 38:**
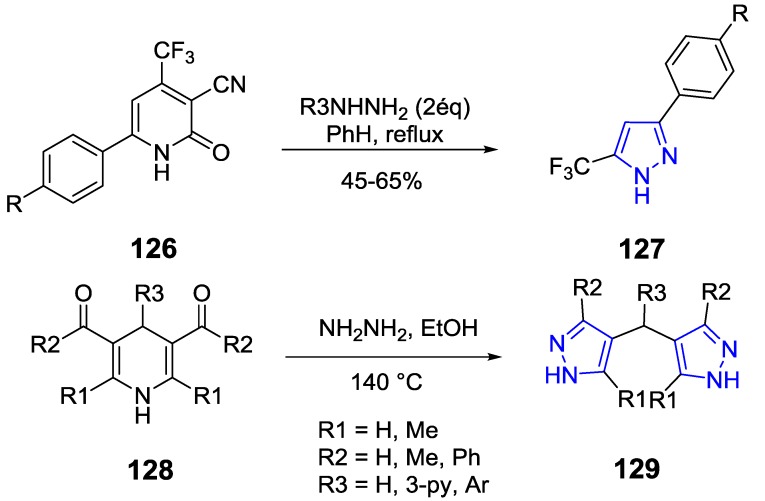
Synthesis of pyrazoles from pyrimidines and pyrimidones.

3-Cyano-4-trifluoromethyl-6-aryl-2(1*H*)-pyridones **126** react with hydrazine hydrate under reflux to give 5-trifluoromethyl-3-arylpyrazoles **127** in 45–65% yields (Scheme 38) [52]. Similarly, the reaction of 3,5-diacyl-1,4-dihydropyridine **128** with hydrazine in ethanol at 140 °C afforded bis-pyrazolyl methanes **129** in good yields (Scheme 38) [53].

#### 2.4.4. From Imidazole

**Scheme 39:**
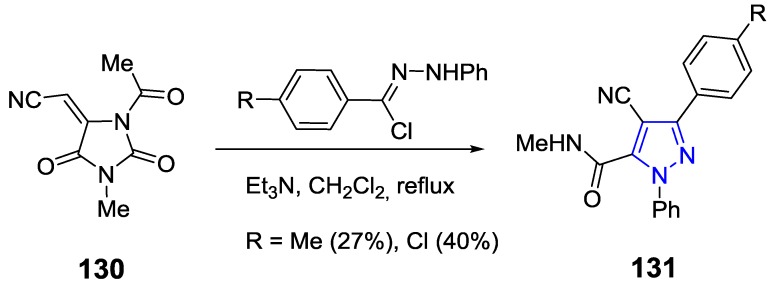
Synthesis of pyrazoles from oxazoles.

Cycloaddition of (5*Z*)-1-acyl-5-(cyanomethylidene)-3-methylimidazolidine-2,4-diones **130** with arylhydrazonyl chloride under basic conditions to give pyrazole-5-carboxamides **131** in moderate 27–40% yields (Scheme 39) [54].

#### 2.4.5. From Oxazoles

**Scheme 40:**
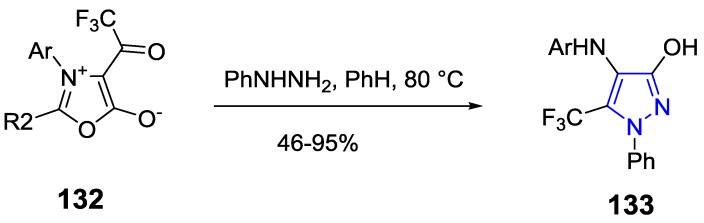
Synthesis of pyrazoles from oxazoles.

5-Trifluoromethyl-3-hydroxypyrazoles **132** were obtained in good yield (46–95%) by heating phenylhydrazine and 4-trifluoroacetyl-1,3-oxazolium-5-olates **133** under reflux of benzene (Scheme 40) [55].

#### 2.4.6. From Tetrazoles

**Scheme 41:**
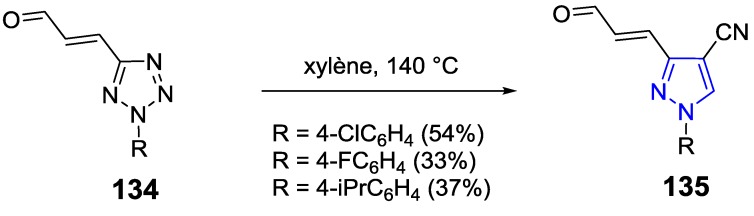
Synthesis of pyrazoles from tetrazoles.

Cyanopyrazoles **135** are readily prepared from tetrazolo[1,5-*b*]pyridazines, tetrazolo[1,5-*a*] pyrimidines, or tetrazolo[1,5-*a*]pyridines. Tetrazolyl acroleins **134** reacts with fumaronitrile in xylene at 140 °C to give the corresponding pyrazole formation **135** (Scheme 41) [56].

#### 2.4.7. From Triazines

**Scheme 42:**
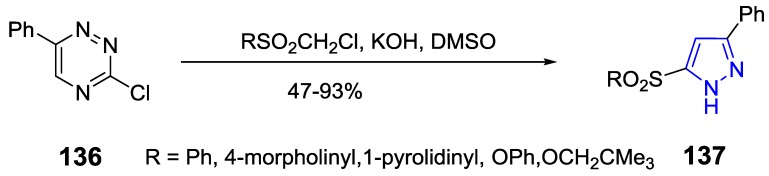
Synthesis of pyrazoles from triazines.

Rykowski et al. [57] proposed a synthesis of pyrazoles, based on the condensation of 3-chloro-6-phenyl-1,2,4-triazines **136** on α-chlorosulfonyls in the presence of potassium hydroxide for obtain the corresponding pyrazoles **137** (Scheme 42).

#### 2.4.8. From 1,5-Benzodiazepin-2-one

**Scheme 43:**
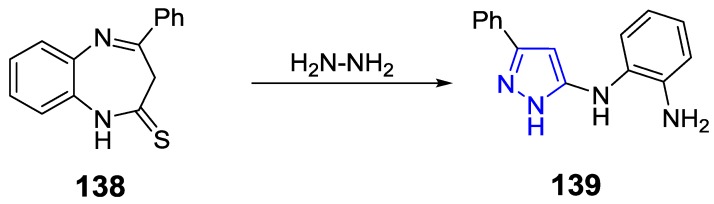
Synthesis of pyrazoles from 1,5-Benzodiazepin-2-one.

Ferfra et al. [58] prepared pyrazole from benzodiazepine-2-thione in one step. They opened the seven-membered ring by reacting hydrazine with benzodiazepine-2-thione **138** to give *o*-aminophenylaminopyrazole **139** (Scheme 43).

#### 2.4.9. From Other Heterocycles

**Scheme 44:**
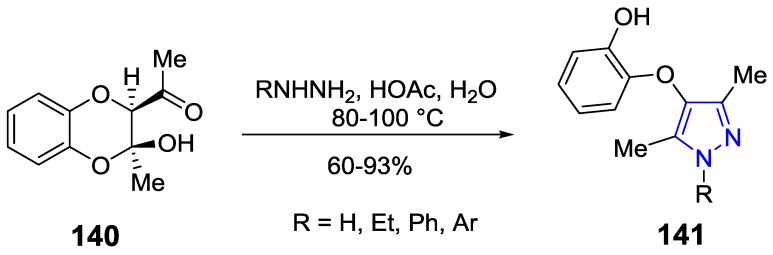
Synthesis of pyrazoles from (*Z*)-3-Acetyl-2-methyl-2,3-dihydro-1,4-benzodioxin-2-ol.

Substituted pyrazoles **141** were synthesized in high yields through the condensation reaction of (*Z*)-3-Acetyl-2-methyl-2,3-dihydro-1,4-benzodioxin-2-ol **140** with arylhydrazines in a mixture of water and acetic acid (Scheme 44) [59].

**Scheme 45:**
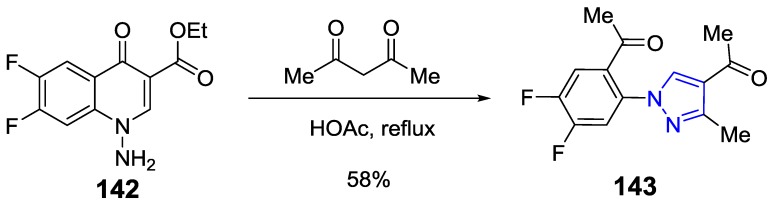
Synthesis of pyrazoles from ethyl-1-amino-6,7-difluorooxoquinolin-4-one-3-carboxylate.

Similarly, condensation of ethyl-1-amino-6,7-difluorooxoquinolin-4-one-3-carboxylate **142** with pentane-2,4-dione in acetic acid at reflux afforded 1-(2-acetyl-4,5-difluorophenyl)-3-methyl-4-acetylpyrazole **143** (Scheme 45) [60].

**Scheme 46:**
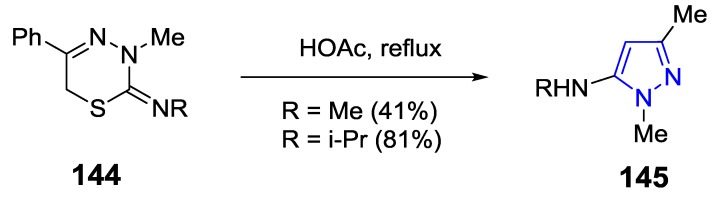
Synthesis of pyrazoles from 3-methyl-6*H*-1,3,4-thiadiazine.

5-Aminopyrazoles **145** were obtained in good yield by heating of 3-methyl-6*H*-1,3,4-thiadiazine **146** under reflux of acetic acid (Scheme 46) [61].

**Scheme 47:**
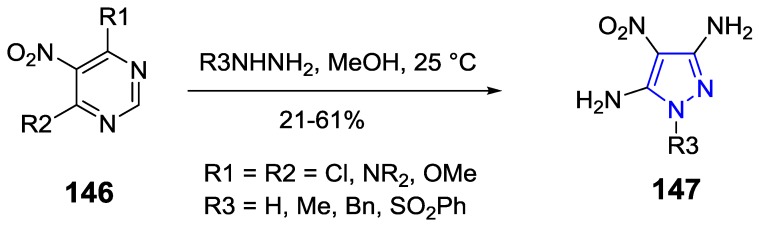
Synthesis of pyrazoles from nitropyrimidine.

On the other hand, the reaction of nitropyrimidine **146** with arylhydrazines in methanol at room temperature, to afford 4-nitro-3,5-diaminopyrazoles **147** in yields of 21–61% (Scheme 47) [62]. 

**Scheme 48:**
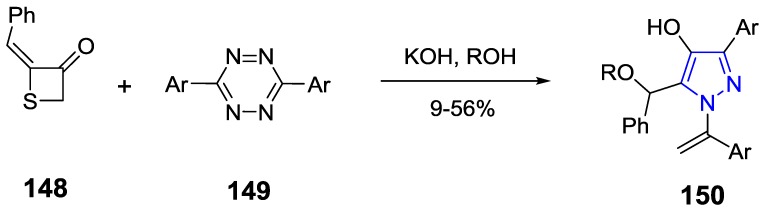
Synthesis of pyrazoles by condensation of thietanone and 1,2,4,5-tetrazines.

Suen et al. prepared a series of substituted 1*H*-pyrazoles **150** by condensing thietanone **148** with 1,2,4,5-tetrazines **149** in the presence of potassium hydroxide (Scheme 48) [63]. 

## 3. Pharmacological Activities

### 3.1. Antibacterial and Antifungal Activity

**Figure 4:**
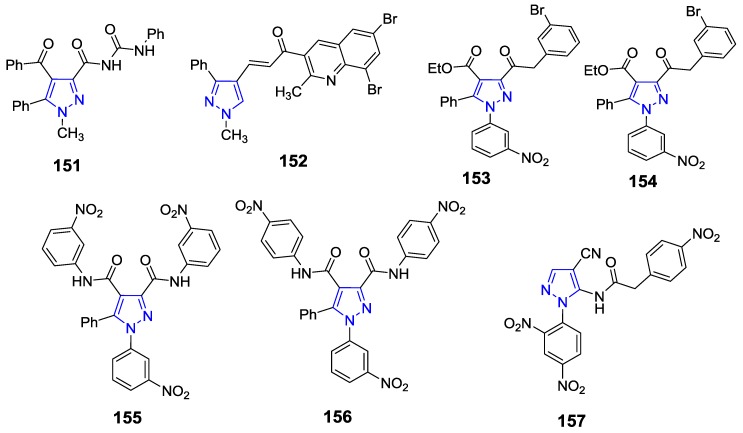
Structures of some pyrazole derivatives as antimicrobial compounds.

Akbas et al. synthesized of series 1*H*-pyrazole-3-carboxylic acid derivatives (Figure 4) and evaluated for their antibacterial activities against *Bacillus cereus*, *Staphylococcus aureus*, *Escherichia coli* and *Pseudomonas putida*. The results showed that the compound **151** was the best compound in the series, exhibiting antibacterial activity against both Gram-positive and Gram-negative bacteria [64]. A series of new pyrazoles containing a quinolinyl chalcone group were synthesized and assessed for antibacterial activity. Compound **152** was the most potent against bacterial and fungal strains [65]. A series of pyrazole-3-carboxylic acid and pyrazole-3,4-dicarboxylic acid derivatives were synthesized and evaluated for their antibacterial and antifungal activities against five bacterial and five fungal pathogens. However, only the molecules **153**, **154**, **155** and **156** demonstrated some inhibitory effects on *Candida parapsilosis*, *Candida tropicalis*, and *Candida glabrata* strains [66]. Rahimizadeh et al. reported the synthesis and antibacterial activity of a series of 5-amido-1-(2,4-dinitrophenyl)-1*H*-pyrazole-4-carbonitriles. Results showed that the compound **157** exhibit antimicrobial activities against methicillin susceptible *S. aureus* and methicillin resistant *S. aureus* with MIC values of 25.1 µM [67]. 

**Figure 5:**
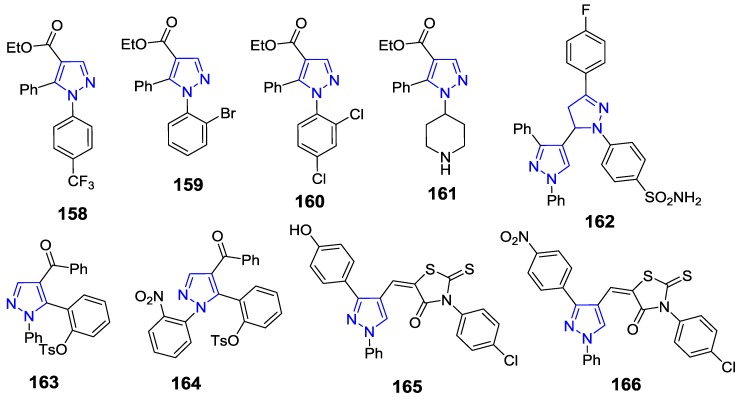
Structures of some pyrazole derivatives with antibacterial activity.

A series of pyrazole derivatives were synthesized and screened for their antibacterial properties against *S. aureus*, *Bacillus subtilis*, *E. coli* and *P. aeruginosa*. Among the tested compounds **158**, **159**, **160** and **161** (Figure 5) have shown excellent antibacterial activity against all the tested bacterial strains as compared with the standard drug ceftriaxone, which was active at 3.125, 1.6125, 1.6125 and 1.6125 µg/mL against *S. aureus*, *B. subtilis*, *E. coli*, and *P. aeruginosa* strains, respectively [68]. 

A series of pyrazolylpyrazolines was synthesized and evaluated for their in vitro anti-microbial activity against two Gram-positive bacteria and two Gram-negative bacteria. The results schowed that the compound **162** was able to inhibit the growth of both the Gram-positive as well as Gram-negative bacteria [69]. A series of pyrazole derivatives were prepared and screened for their anti-bacterial and antifungal activities using ampicillin and norcadine as standard drugs. All the compounds were screened for their antimicrobial activities. The results for these derivatives showed good antibacterial activity for **163** and **164** [70].

B’Bhatt and Sharma synthesized a series of 3-(4-chlorophenyl)-5-((1-phenyl-3-aryl-1*H*-pyrazol-4-yl)methylene)-2-thioxothiazolidin-4-ones. All the synthesized compounds were screened for in vitro antibacterial activity against *E. coli*, *P. aeruginosa*, *S. aureus*, and *S. pyogenes* and in vitro anti-fungal activity, these compounds were tested against *C. albicans*, *A. niger* and *A. clavatus* using ampicillin and griseofulvin as standard drugs. Compound **165** was found as a potent compound against *E. coli*, while compound **166** was found to be potent against *S. aureus*, *S. pyogenes* and was found to have very good activity against *C. albicans* [71]. 

**Figure 6:**
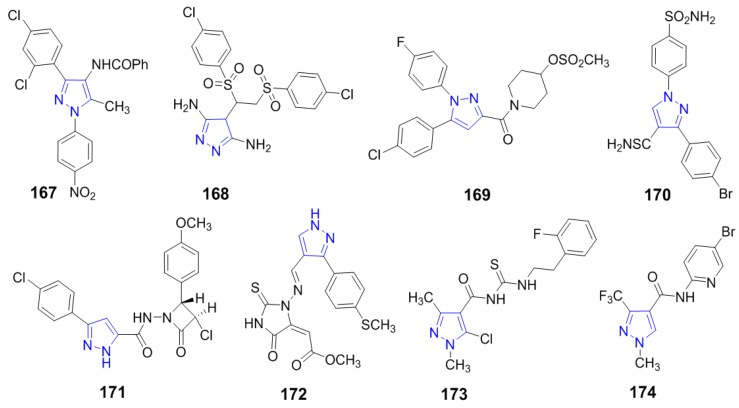
Pyrazole derivatives with antimicrobial activity.

1,3,4,5-Tetrasubstituted pyrazole derivatives were synthesized and tested for anti-microbial activity against *S. aureus*, *E. coli*, *Aspergillus flavus* and *C. albicans*. Compound **167** showed promising antibacterial and antifungal activity [72]. Padmaja et al. reported the synthesis and antimicrobial activity of substituted pyrazoles. Results showed that the compound with sulfone moieties **168** displayed the maximum activity [73]. Novel 1,5-diaryl pyrazole derivatives were synthesized and screened for their antimicrobial activity against *E. coli*, *S. aureus*, *P. aeruginosa*, *K. pneumonia* and for their antifungal activity against *A. flavus*, *A. fumigates*, *P. marneffei* and *T. mentagrophytes*. Compound **169** (Figure 6) exhibited good antibacterial and antifungal activity with MIC value of 12.5 mg/mL [74]. A series of 1,3-diarylpyrazoles derivatives were synthesized and evaluated for their in vitro antibacterial activity against *S. aureus*, *B. subtilis*, *E. coli*, *P. aeruginosa*, and in vitro antifungal activity against *A. niger* and *A. flavus*. Compound **169** exhibited moderate antibacterial and antifungal activities against the tested bacteria and fungi [75]. A series of 3-(4-chlorophenyl)-4-substituted pyrazoles were synthesized and tested for antifungal activity against a pathogenic strain of fungi and antibacterial activity against Gram-positive and Gram-negative organisms. Among those tested, compound **171** (Figure 6) showed good to excellent antimicrobial at MIC values of 0.0025 to 12.5 µg/mL against all the selected pathogenic bacteria and fungi [76].

**Figure 7:**
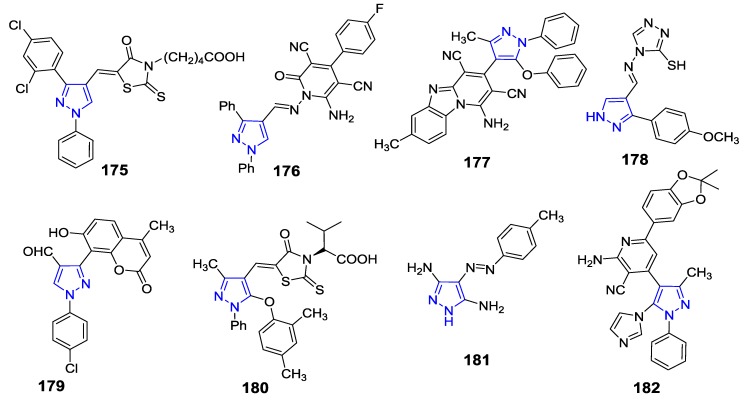
Pyrazole derivatives showing antimicrobial activity.

A series of novel imidazole derivatives containing substituted pyrazole moiety were synthesized by Vijesh et al. and screened for antifungal and antibacterial activities. Among the synthesized compounds, compound **172** was found to be potent antimicrobial agent [77]. Several pyrazole acyl thiourea derivatives were synthesized and antifungal activity against *G. zeae*, *F. oxysporum*, *C. mandshurica*. Compound **173** displayed good antifungal activities against the tested fungi [78]. A series of *N*-(substituted-pyridinyl)-1-methyl(phenyl)-3-trifluoromethyl-1*H*-pyrazole-4-carboxamide derivatives were synthesized by Wu et al. and evaluated in vitro against three kinds of phytopathogenic fungi (*G. zeae*, *F. oxysporum*, *C. mandshurica*). The results showed that the compound **174** displayed more than 73% inhibition activities against *G. zeae* at 100 µg/mL [79]. A series of 1,3-diaryl pyrazole derivatives bearing rhodanine-3-fatty acid moieties (Figure 7) were synthesized and investigated for their in vitro antimicrobial activities against various Gram-positive and Gram-negative bacteria. Compound **175** was found active against the methicillin-resistant *Staphylococcus aureus* (MRSA) with a MIC of 2 mg/mL [80]. A series of novel pyrazole derivatives were synthesized by Desai et al. and screened for their in vitro antibacterial activity against *S. aureus*, *S. pyogenes*, *E. coli*, *P. aeruginosa*. The results indicated the compound **176** showed potent antibacterial activity against *S. aureus*, *E. coli* at 12.5 mg/mL [81]. Pyrido[1,2-*a*]benzimidazole derivatives bearing the aryloxypyrazole nucleus were synthesized and investigated for in vitro antimicrobial activity. Compound **177** was found active against employed pathogens [82]. Malladi et al. synthesized a series of new Schiff bases containing pyrazole rings and screened them for their antibacterial (*S. aureus*, *B. subtilis*, *E. coli* and *P. aeruginosa*) activity. The results revealed that, compound **178** exhibited significant antibacterial activity against the tested microorganisms with MIC value of 1.61 mg/mL [83]. A series of formyl-pyrazoles derivatives were synthesized and screened in vitro for their antibacterial and antifungal activities. The compounds **179** exhibited promising antifungal and antibacterial activity with MIC values of 15–60 µg/mL against the different organisms tested [84]. The anti-bacterial activity of 5-aryloxypyrazole derivatives was reported by Song et al. Compound **180** showed good inhibitory activity against selected methicillin resistant and quinolone-resistant *S. aureus* (MRSA, QRSA) with MIC values in the range of 2–4 µg/mL [85]. Sayed and co-workers described the synthesis and antimicrobial activity of new pyrazole derivatives. The results revealed that the compound **181** showed significant antimicrobial activity against the tested microorganisms [86]. A series of novel 5-imidazopyrazole derivatives were synthesized and evaluated for their in vitro antibacterial activity against a panel of pathogenic strains of bacteria and fungi. Compound **182** exhibited excellent antimicrobial activity as compared with the first line drugs [87].

**Figure 8:**
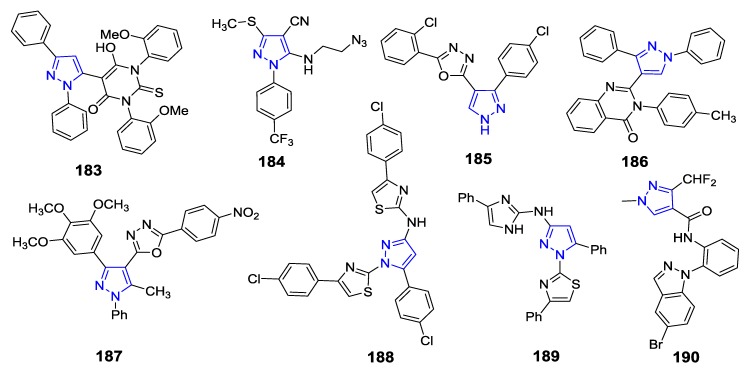
Pyrazole derivatives with antimicrobial activity.

Pyrimidine pyrazole derivatives (Figure 8) were synthesized by Kumar et al. and screened for their antimicrobial activity against bacteria and fungi. Among all the compounds, compound **183** was found to be the most active with MIC value of 31.25 µg/mL against *S. aureus* and *B. cereus* [88]. Several pyrazole derivatives were synthesized and evaluated for their fungicidal activities against *Botrytis cinerea*, *Rhizoctonia solani*, *Valsa mali Miyabe et Yamada*, *Thanatephorus cucumeris, Fusarium oxysporum*, and *Fusarium graminearum*. The results indicated that the compound **184** showed the highest activity, with EC_50_ values of 2.432, 2.182, 1.787, 1.638, 6.986 and 6.043 μg/mL against *B. cinerea*, *R. solani*, *V. mali*, *T. cucumeris*, *F. oxysporum*, and *F. graminearum*, respectively [89]. A series of 2,5-disubstitued-1,3,4-oxadiazole derivatives bearing a pyrazole moiety were synthesized and screened for their antibacterial activity against *E. coli*, *S. aureus* and *P. aeruginosa*, and for antifungal activity against *A. flavus*, *C. keratinophilum* and *C. albicans*. The evaluation of antimicrobial screening revealed that compounds **185** exhibited excellent activity [90]. A new series of quinazolin-4(3H)-one derivatives containing a (1,3-diphenyl-1*H*-pyrazol-4-yl) core were synthesized by Mehta et al. and screened for their antimicrobial, antifungal activities. The results showed that the compound **186** was found the most active against the tested pathogens [91]. A series of pyrazole derivatives containing 1,3,4-oxadiazoles moiety were synthesized by Ningaiah et al. and evaluated for their antimicrobial activity. Among the synthesized compounds, compound **187** showed good to moderate activity with MIC in the range of 20–50 μg/mL against bacteria and 25–55 μg/mL against fungi [92]. A series of pyrazole derivatives linked thiazole and imidazole were prepared and tested for antimicrobial activity. The compounds **188** and **189** exhibited excellent antibacterial and antifungal activities [93]. Du et al. synthesized a series of novel 3-(difluoromethyl)-1-methyl-1*H*-pyrazole-4-carboxylic acid amides and tested in vitro for their activities against seven phytopathogenic fungi. Among them *N*-(2-(5-bromo-1*H*-indazol-1-yl)-phenyl)-3-(difluoromethyl)-1-methyl-1*H*-pyrazole-4-carboxamide (**190**) exhibited higher antifungal activity against the seven phytopathogenic fungi than boscalid [94]. 

**Figure 9:**
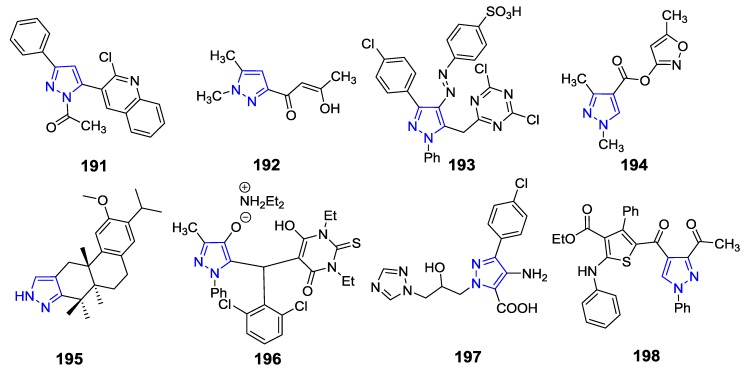
Pyrazole derivatives with antimicrobial activity.

Miniyar et al. synthesized a novel series of pyrazole derivatives bearing a 2-chloroquinoline ring (Figure 9) and screened them for antibacterial and antifungal activity. Among the series, compound **191** was found moderately active against *A. fumigatus*, *P. notatum*, *B. subtilis* and *E. coli* with MIC values of 48, 46, 44 and 87 µg/mL, respectively [95]. Radi et al. reported the synthesis and antifungal activity of novel pyrazole derivatives. Compound **192** had the most potent activity against *Fusarium oxysporum* f.sp *albedinis FAO* with n IC_50_ value of 0.055 µM [96]. A series of new pyrazole derivatives were synthesized and evaluated for antimicrobial activity. Compound **193** showed the highest activities against tested organisms [97]. A series of isoxazolol pyrazole carboxylate derivatives were synthesized and bioassayed in vitro against four types of phytopathogenic fungi (*Alternaria porri*, *Marssonina coronaria*, *Cercospora petroselini* and *Rhizoctonia solani*). The results showed that the compound **194** exhibited significant antifungal activity against *R. solani*, with an EC_50_ value of 0.37 μg/mL [98]. A series of novel diterpene derivatives containing pyrazole ring were synthesized and investigated for their activity against *S. aureus* Newman strain and multidrug-resistant strains (*NRS-1*, *NRS-70*, *NRS-100*, *NRS-108* and *NRS-271*). Among the compounds tested, compound **195** showed the most potent activity with MIC values of 0.71–3.12 µg/mL against five multidrug-resistant *S. aureus* [99]. Elshaier et al. described the synthesis and antimicrobial activity of new series of pyrazole-thiobarbituric acid derivatives. Compound **196** was the most active against *C. albicans* with MIC = 4 µg/L, and exhibited the best activity against *S. aureus*, *B. subtilis* and *E. faecalis* with MIC = 16 µg/L [100]. A series of novel pyrazole-5-carboxylate derivatives containing a *N*-triazole scaffold were synthesized and screened for their in vitro antimicrobial activity against three Gram-positive and Gram-negative bacteria as well as three fungi. The results revealed that the compound **197** was more potent antibacterial activity against all bacterial strains, and showed excellent antifungal activities against *A. niger* and *C. albicans* in MIC = 4 µg/L [101]. Several new pyrazole derivatives incorporating a thiophene moiety were synthesized and evaluated for their antibacterial and antifungal activities. The results showed that compound **198** revealed a high degree of antibacterial activity towards *Pseudomonas aeruginosa* and inhibition effects against *Escherichia coli* [102].

**Figure 10:**
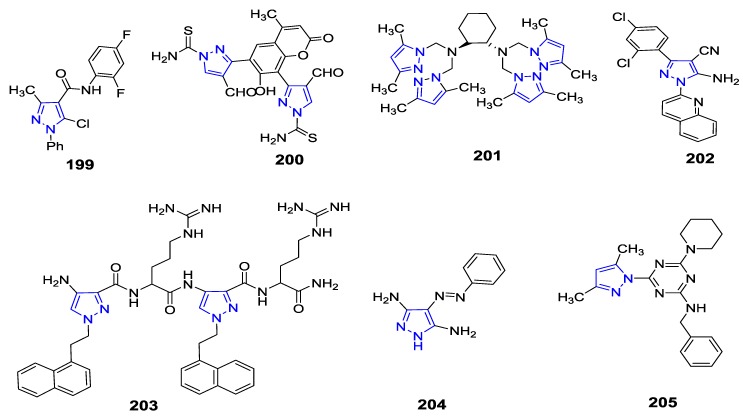
Pyrazole derivatives with antimicrobial activity.

A series of novel pyrazole amide derivatives (Figure 10) were synthesized and evaluated in vivo for their antifungal activity against *P. ultimum* Trow, *Phytophthora infestans* (Mont.) De Bary, *Corynespora cassiicola*, *Botrytis cinerea* and *Rhizoctonia solani*. The fungicidal results indicated that the compound **199** exhibited good control efficacy (77.78%) against *P. ultimum* Trow at a concentration of 100 µg/mL [103]. Nagamallu et al. synthesized a series of novel coumarin pyrazole hybrids were synthesized and evaluated for antimicrobial activities. Among the series, compound **200** showed excellent antimicrobial activity against different bacterial and fungal strains with MIC values in range of 12.5–50 µg/mL [104]. In another sequence of pyrazole derivatives synthesized by Radi et al., a series of new *N*,*N*,*N′*,*N′*-tetradentate pyrazoly derivatives were screened for their antimicrobial activity. Among the compound **201** was exceedingly antifungal active against budding yeast (*Saccharomyces cerevisiae*) cells with MIC = 500 µM [105]. A series of quinoline derivatives bearing pyrazole moiety were synthesized and evaluated for their antibacterial and antifungal activities. Pyrazole compound **202** showed better results when compared with the reference drugs as revealed from their MIC values (0.12–0.98 µg/mL) against the following human pathogens strains: *S. flexneri*, *A. clavatus*, *C. albicans*, *P. vulgaris*, *S. epidermidis* and *A. fumigatus* [106]. Ahn et al. reported the synthesis and antimicrobial activities of pyrazole-derived amino acids and peptidomimetics. Compound **203** showed the good activity against *E. coli*, *P. aeruginosa*, *S. epidermidis* and *S. aureus* with MIC values range from 4 to 32 µg/mL [107]. Nada et al. synthesized new series pyrazol-based derivatives and tested for their antimicrobial against bacterial strains *E. coli* and *S. aureus*. Data showed that pyrazole compound **204** was potent only at 0.075 mg/mL against the tested microorganisms [108]. A new series of pyrazole-containing s-triazine derivatives were synthesized by Sharma et al. and investigated for antimicrobial and antifungal activity against the growth of several microorganisms. Compound **205** exhibited antibacterial activity against bacterial strains: *P. aeruginosa*, *M. luteus* and methicillin-resistant *S. aureu* [109].

**Figure 11:**
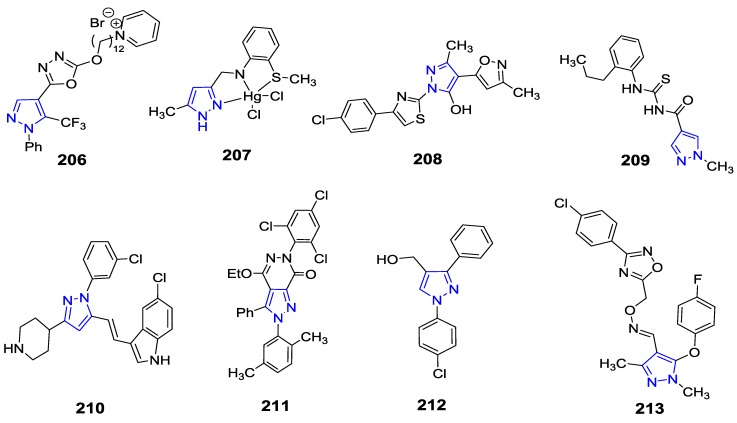
Pyrazole derivatives with antimicrobial activity.

A series of pyridinium-tailored 5-trifluoromethylpyrazoles containing 1,3,4-oxadiazole moieties (Figure 11) were constructed by Zhou et al. and evaluated in vitro for antimicrobial activities against three types of pathogenic bacteria and six fungal strains. Among these derivatives, compound **206** showed potent antibacterial effects against *Xanthomonas oryzae* pv. oryzae and *Xanthomonas axonopodis* pv. citri with EC_50_ values within 0.467 and 0.600 µg/mL, respectively [110]. Recently, Mondal et al. reported the syntheses and antimicrobial activity of some Ni(II), Cd(II) and Hg(II) complexes of a pyrazole containing Schiff base ligands. The results showed that the complex **207** demonstrated highest antimicrobial agents against both Gram positive and Gram negative bacteria [111]. A series of isoxazolyl thiazolyl pyrazoles were synthesized by Mor et al. and evaluated in vitro for antimicrobial activity. Amongst the newly synthesized compounds, compound **208** was found to exhibit the promising antibacterial activity against *S. aureus* [112]. A series of *N*-(1-methyl-1*H*-pyrazole-4-carbonyl)-thiourea derivatives were prepared and evaluated for antibacterial and antifungal activities. Compound **209** was found to be the most potent antimicrobial agent [113]. The synthesis and antibacterial activity of pyrazole derivative were described by Tanitame et al. Results showed that the compound **210** possesses potent antibacterial activity and selective inhibitory activity against bacterial *topoisomerases* (*MRSA*, *PRSA* and *VRE*) with MIC = 1–2 µg/mL [114]. Antibacterial activity of a-Acyl-pyrazole-3-carboxylic acids was described by Çetin et al. Results showed that the compound **211** has very potent antibacterial activity against *B. subtilis*, *S. aureus*, *E. coli*, *P. aeruginosa*, and *K. pneumonia* [115]. A series of novel pyrazolyl alcohols were prepared and evaluated for their anti-bacterial activity. The compound **212** displayed the potent anti-bacterial activity against *Micrococcus luteus* (MIC 3.9 and MBC 7.81 µg/mL) [116]. A series of novel pyrazole oxime derivatives were synthesized and evaluated for fungicidal activities in vivo against *Pseudoperonospora cubensis*. Among the synthesized compounds, compound **213** (EC_50_ = 0.51 µg/mL) showed excellent fungicidal activity against *P. cubensis* comparable or better than that of the control pyraclostrobin (EC_50_ = 4.59 µg/mL) [117]. 

### 3.2. Anticancer Activity

**Figure 12:**
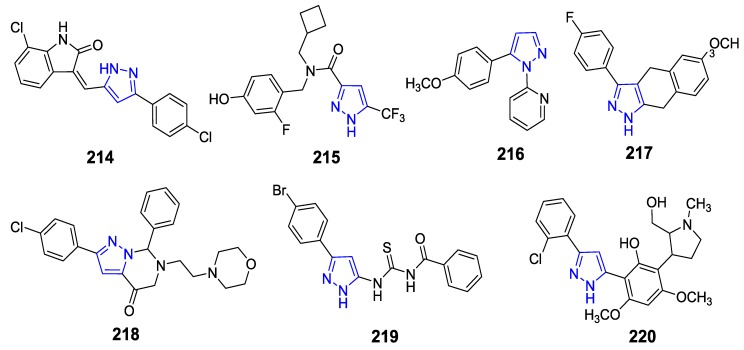
Structures of pyrazole derivatives with anticancer activity.

A new class of pyrazole-oxindole (Figure 12) were synthesized and investigated for their antiproliferative activity on different human cancer cell lines. Among the active compound **214** manifested significant cytotoxicity and inhibited tubulin assembly with IC_50_ 3 µM [118]. Xu et al. reported compound **215** as hPKM2 activator with (AC_50_ = 0.011 µM) as the most active anticancer agent with IC_50_ of 5.94 and 6.40 µM against A549 and NCI-H1299 cell lines respectively [119]. Pyrazoles derivatives synthetized by Viale et al. were secrenned for their anti-proliferative activity. The comparative data once again demonstrate the good antiproliferative activity of these compounds, confirming that **216** may be considered as a good lead compound for subsequent development [120]. Several pyrazole derivatives were reported. The bioactivities of the new compounds were evaluated through in vitro assays for endothelial cell proliferation and tube formation. Results indicated that the synthetized compound **217** exhibit potent cytostatic properties displaying IC_50_ values of 1.5 µM [121]. A series of pyrazolo[1,5-*a*]pyrazin-4(5*H*)-ones were synthesized and tested to determine their ability to inhibit the growth of A549 and H322 cancer cells. Results showed that the compound **218** exerted good activity, with an IC_50_ of of 24.2 and 29.4 µM against A549 and H322 cells lines, respectively [122]. Balbi et al. synthesized a series of pyrazole derivatives and studied their antiproliferative activity in human ovarian adenocarcinoma A2780 cells, human lung carcinoma A549 cells, and murine P388 leukemia cells. compound **216** demonstated significant antiproliferative agent [123]. A series of substituted pyrazole compounds were synthesized and evaluated in vitro for their anticancer effects on a panel of 60 cellular lines. Results showed that the compound **219** presented significant growth inhibitory effects on the tested cancer cells [124]. A series of 3,5-diarylpyrazole derivatives were synthesized and evaluated for their anticancer activity against five cell lines (breast cancer, prostate cancer, promyelocytic leukemia, lung cancer, colon cancer). Compound **220** was identified as a potent anticancer agent against all selected cell lines [125].

**Figure 13:**
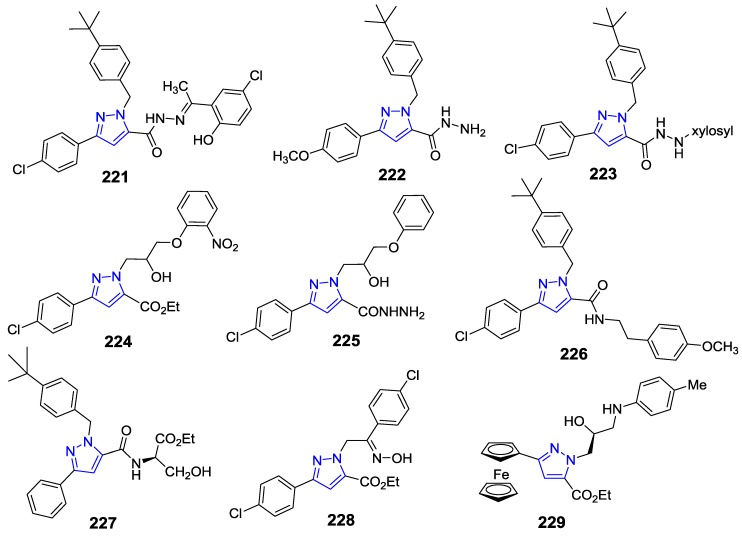
Pyrazole derivatives showing anticancer activity.

Miao and co-worker synthesized a series of pyrazoles derivatives (Figure 13) and investigated the effects of all the compounds on A549 cell growth. The results showed that the compounds **221–229** possessed the highest growth inhibitory effect and induced apoptosis of A549 lung cancer cells [126,127,128,129,130,131,132,133,134].

**Figure 14:**
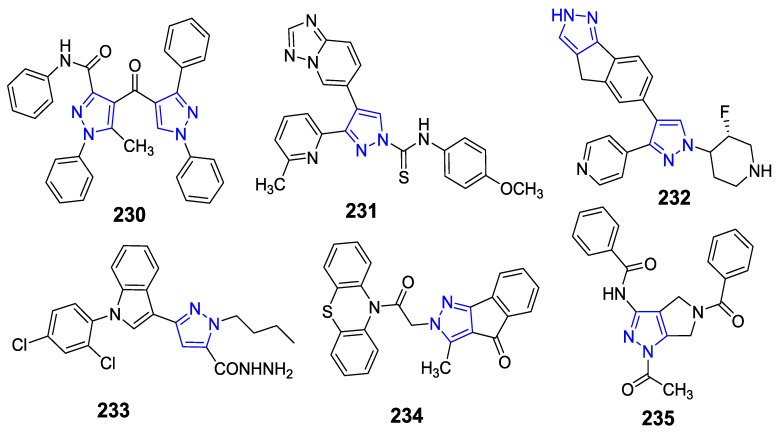
Pyrazole derivatives with anticancer activity.

Farag et al. synthesized a series of pyrazole derivatives (Figure 14) and evaluated them for their antitumor activity. Compound **230** was reported as the most potent anticancer agent against Ehrlich ascites carcinoma tumor cells [135]. Several [1,2,4]triazolo[1,5-*a*]pyridine-bearing pyrazole moieties were prepared by Jin et al. and evaluated for their ALK5 inhibitory activity in an enzyme assay and in a cell-based luciferase reporter assay. The compound **231** exerted the maximun anticancer actvity with IC_50_ of 0.57 nM [136]. Newhouse et al. reported the synthesis and anticancer activity of pyrazole derivatives. Results showed that the compound **232** exhibit excellent enzyme activity (B-Raf inhibitor) [137]. A series of novel 3-(1*H*-indole-3-yl)-1*H*-pyrazole-5-carbohydrazide derivatives were synthesized and evaluated for their in vitro cytotoxic activity against A549, HepG-2, BGC823 and BT474 cell lines. Compound **233** exhibited more potent antiproliferative activity [138]. A new family of protein farnesyltransferase inhibitors, based on a phenothiazine containing pyrazole scaffold, was synthesized and evaluated for their antiproliferative activity on a NCI-60 cancer cell line panel. Indenopyrazole **234** exhibited the most potent in vitro cytostatic activity inhibiting the growth of HCT-116, LOX IMVI and SK-MEL-5 cell lines [139]. A series of 1,4,5,6-tetrahydropyrrolo[3,4-*c*]pyrazole derivatives were synthesized and initially evaluated for their in vitro anticancer activity against human colon carcinoma HCT-116 cell line. These results indicated that the compound **235** showed considerable in vitro anticancer activity with IC_50_ of 0.58 µM [140]. 

**Figure 15:**
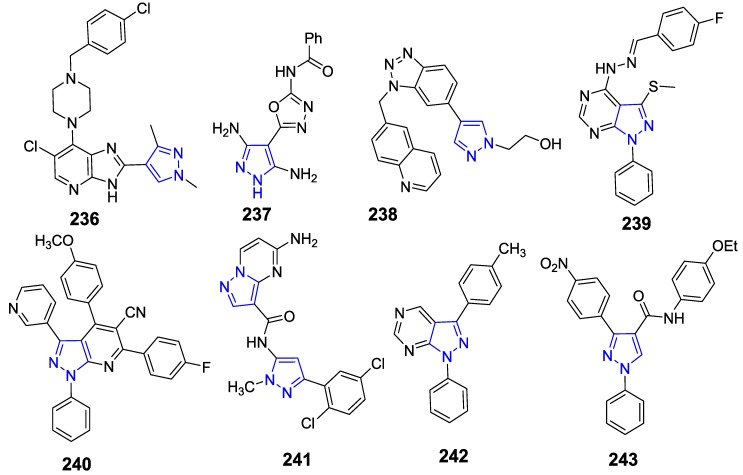
Pyrazole derivatives with anticancer activity.

A 1,3-dimethyl-1*H*-pyrazole-based series of imidazo[4,5-*b*]pyridines (Figure 15) were synthesized by Bevetsias et al. and assessed for their FLT3/Aurora inhibitory activity. Amongst the series, compound **236** displayed antiproliferative activity in a range of human tumor cell lines, including HCT116 human colon carcinoma (GI_50_ = 0.300 μM) and the human FLT3-ITD positive AML cell lines MOLM-13 (GI_50_ = 0.104 μM) and MV4−11 (GI_50_ = 0.291 μM) [141]. Some novel 1,3,4-oxadiazole derivatives carrying pyrazole rings were developed and evaluated for their antitumor and cytotoxic activities. The results revealed that the compound **237** displayed promising in vitro antitumor activity in the 4-cell lines assay [142]. Cui et al. identified a new c-MET inhibitor. Compound **238** demonstrated effective tumor growth inhibition in c-MET dependent tumor models with good oral PK properties and an acceptable safety profile in preclinical studies. Compound **238** progressed to clinical evaluation in a Phase I oncology setting [143]. A series of new pyrazolo[3,4-*d*]pyrimidines were synthesized and investigated for their anti-tumor activity. Compound **239** was the most active compound with IC_50_ equal to 7.5 nM [144]. A series of pyrazolo[3,4-*b*]pyridine derivatives were prepared by El-Borai et al. and tested for antitumor activity against liver cell line. Compounds **240** showed the highest activity with IC_50_ 3.43 µg/mL [145]. Hanan et al. developed a new series of pyrazoles derivatives bearing pyrazolo[1,5-*a*]pyrimidine scaffold and optimized for their activity against Janus kinase *2* inhibitors. Compound **241** was identified as a potent inhibitor of Jak2 (Ki = 0.1 nM) and demonstrated a time-dependent knock-down of pSTAT5 (IC_50_ = 7.4 nM) [146]. A novel series of pyrazolo[3,4-*d*]pyrimidines derivatives were developed by Huang et al. and evaluated for their anticancer activity. Among the examples, compound **242** possessed better potency against NCI-H226 and NPC-TW01 cancer cells with GI_50_ values 18 mM [147]. Li et al. synthesized a series of 1*H*-pyrazole-4-carboxamide derivatives and evaluated for their potential antiproliferation activity and Aurora-A kinase inhibitory activity. Among all the compounds, compound **243** possessed the most potent biological activity against HCT116 and MCF-7 cell lines with IC_50_ values of 0.39 µM and 0.46 µM, respectively. Compound **243** also exhibited significant Aurora-A kinase inhibitory activity (IC_50_ = 0.16 ± 0.03 µM) [148]. 

**Figure 16:**
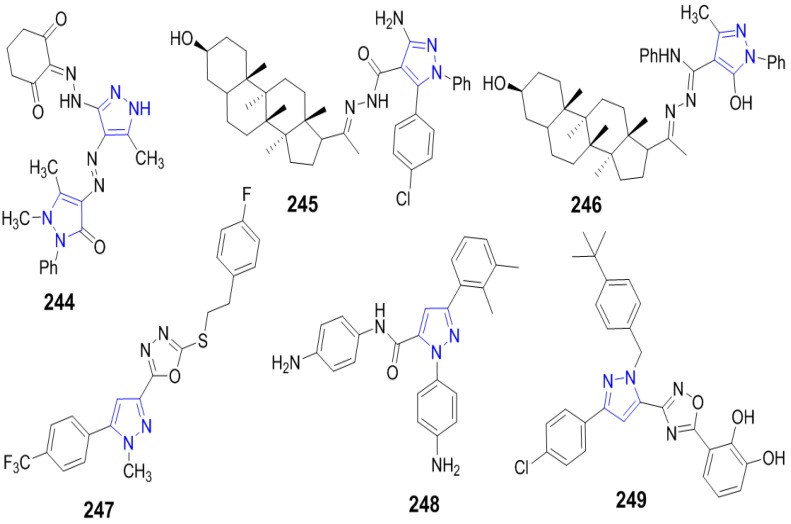
Pyrazole derivatives with anticancer activity.

A series of pyrazoles derivatives (Figure 16) were synthesized and screened for their cytotoxic activity. The results showed clearly that compound **244** displayed promising in vitro anticancer activity against four different cell lines (HepG2, WI 38, VERO and MCF-7) [149]. Mohareb et al. synthesized of new series of pyrazoles derivatives containing pregnenolone moiety and evaluated for their cytotoxicity against three human tumor cell lines. Compound **245** was found to exhibit much higher inhibitory effects towards adenocarcinoma *(*MCF-7), non-small cell lung cancer (NCI-H460) and CNS cancer (SF-268), with IC_50_ values 0.01, 0.02 and 0.04 µM, respectively [150]. The same authors synthesized a series of pyrazolyl semicarbazidoandrrostane derivatives and tested their cytotoxicity activity, whereby compound **246** displayed excellent cytotoxicity with IC_50_ values 0.02, 0.01 and 0.03 µM against MCF-7, NCI-H460 and SF-268 cells, respectively [151]. Puthiyapurayil et al. reported the synthesis and anticancer activity of pyrazoles derivatives bearing a *S*-substituted-1,3,4-oxadiazole moiety. Amongst the tested compounds, the compound **247** was the most promising anticancer agent, with an IC_50_ value of 15.54 µM in MCF-7 cells, compared to doxorubicin as standard drug [152]. Small pyrazole derivatives were developed and evaluated in vitro for their anticancer activity on HCC-derived cell lines. The compound **248** selected as potential agents active against hepatocellular carcinoma (HCC) and showed a promising inhibitory growth efficacy (IC_50_ = 50 mM) in SNU449 cell line [153]. Synthesis and anticancer activities of new pyrazole derivatives bearing 1,2,4-oxadiazole moiety were reported by Vujasinovic et al. Therefore, compound **249** presents a good starting point for design of new, more potent and safer anticancer therapeutics [154].

**Figure 17:**
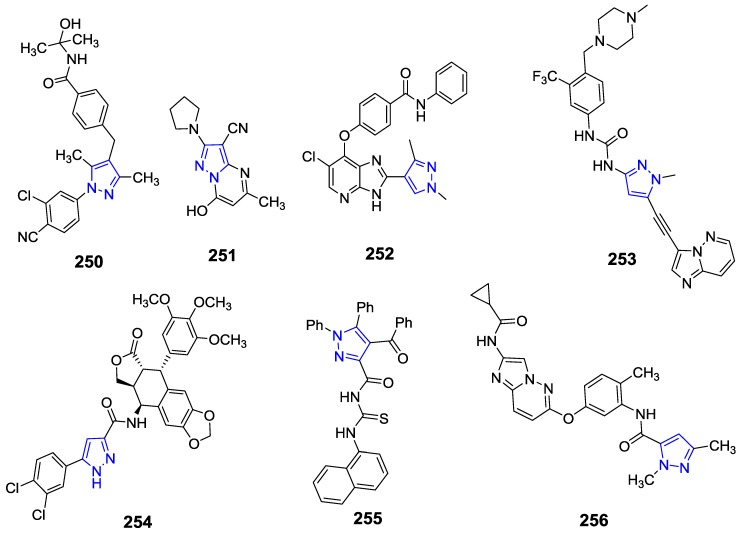
Pyrazole derivatives with anticancer activity.

Yamamoto et al. synthesized a series of 4-arylmethyl-1-phenylpyrazole derivatives (Figure 17) and evaluated their potential as new-generation androgen receptor (AR) antagonists therapeutically effective against castration-resistant prostate cancer (CRPC). Compound **250** exhibited potent antitumor effects against a CRPC model of LNCaP-hr cell line in a mouse xenograft model [155]. Some new pyrazolo[1,5-*a*]-pyrimidines were prepared and evaluated for their cytotoxicity activity against Vero cells. Compound **251** was reported as the most active compounds of the series [156]. Bevetsias et al. synthesized a new 1,3-dimethyl-1*H*-pyrazole-based series of imidazo[4,5-*b*]pyridines and tested them for their Aurora-A kinase inhibitory activity. Amongst the series, compound **252** displayed antiproliferative activity in a range of human tumor cell lines, including Aurora-A (GI_50_ = 0.067 μM) Aurora-A (IC_50_ = 12.71 μM) and in HCT116 human colon carcinoma cells (p-T288, IC_50_ = 0.065 μM; p-HH3, IC_50_ = 24.65 μM) [157]. Desai et al. synthesized of novel *Abl* kinase inhibitors. Within just **253** compounds, identified a novel template and hinge binding motif with pIC_50_ > **8** against Abl kinase both wild type and clinically relevant mutants [158]. A series of pyrazoles derivatives bearing 4b-amidopodophyllotoxin rings were synthesized and evaluated for anticancer activity against five human cancer cell lines. Among the series, one of the compounds, 254, showed significant antiproliferative activity in A549 (lung cancer) cell line [159]. Koca et al. a new series of acyl thiourea derivatives containing pyrazole ring were prepared and evaluated for their anticancer activity. Compound **255** was reported as the most potent anticancer agent [160]. Miyamoto et al. described the synthesis and anticancer activity of 1*H*-pyrazole-5-carboxamide deriavtives. Compound **256** emerged as a highly potent VEGF receptor 2 kinase inhibitor with an IC_50_ value of 0.95 nM and suppressed proliferation of VEGF-stimulated human umbilical vein endothelial cells with an IC_50_ of 0.30 nM [161].

**Figure 18:**
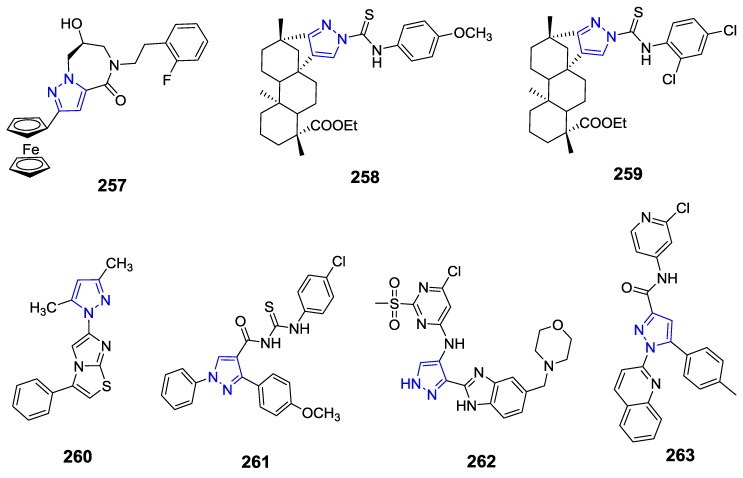
Pyrazole derivatives with anticancer activity.

A series of novel pyrazolo[1,5-*a*][1,4]dia-zepin-4-one derivatives containing ferrocene scaffolds (Figure 18) were synthesized and evaluated for their anticancer activity against A549, H322 and H1299 lung cancer cells. Of them, compound **257** possessed notable cytotoxicity and selectivity for A549 vs. H1299 and H322 lung cancer cells [162]. Zhu et al. prepared a series of novel carbothioamide-pyrazole derivatives and tested for their in vitro cytotoxic activities against four human tumor cell lines. Results indicated that compound **258** exhibited potent cytotoxicity (IC_50_ = 6.51 µM) against Raji cell [163]. 

A series of novel isosteviol-fused pyrazole derivatives were synthesized and evaluated in vitro for their antiproliferative activities on four human malignant cell lines. Results revealed that compound **259** displayed better cytotoxities with IC_50_ values: 2.71, 3.18, 1.09 and 13.52 mM against SGC 7901, A549, Raji and HeLa, respectively, compared to cisplatin (IC_50_ values: 7.56, 17.78, 17.32 and 14.31 mM, respectively) [164]. A series of imidazo[2,1-*b*]thiazoles bearing pyrazole moieties were synthesized and tested in vitro for their anticancer activity. Result showed that compounds **260** had the highest anticancer effect against CNS SNB-75 and renal UO-31 cancer cell lines [165]. Sun et al. synthesized a series of 1*H*-pyrazole-4-carboxamide derivatives and evaluated for their antiproliferative activities as CDKs inhibitors. Among all the synthesized compounds, compound **261** inhibited CDK2 with an IC_50_ value 25 nM [166]. Novel pyrazole–benzimidazole derivatives were synthesized and evaluated for their anticancer activities against cancer cell lines U937, K562, A549, LoVo and HT29 and were screened for Aurora A/B kinase inhibitory activity in vitro. The **262** demonstrated significant cancer cell lines and Aurora A/B kinase inhibitory activities [167]. A series of novel 5-(*p*-tolyl)-1-(quinolin-2-yl)pyrazole-3-carboxylic acid derivatives were synthesized and assessed for their antiproliferative activities against three human cancer cell lines (Huh7, human liver; MCF7, breast and HCT116, colon carcinoma cell lines). Compound **263** exhibited promising cytotoxic activity against all cell lines with IC_50_ values of 1.6 µM, 3.3 µM and 1.1 µM for Huh7, MCF7 and HCT116 cells, respectively [168].

**Figure 19:**
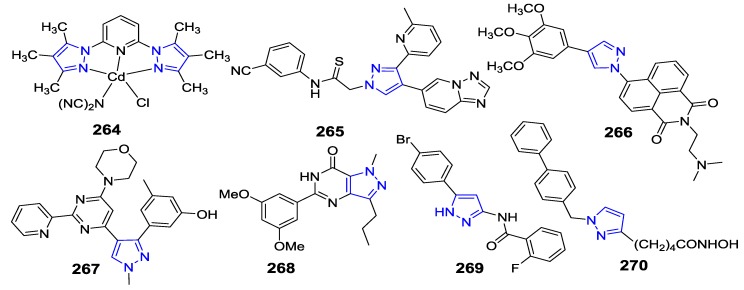
Pyrazole derivatives with anticancer activity.

Cd(II) complexes of tridentate nitrogen donor ligand, 2,6-bis(3,4,5-trimethylpyrazolyl)pyridine (Figure 19) were synthesized and tested for cytotoxic activity against the human carcinoma cell lines HEP3B (hepatocellular carcinoma), PC3 (prostate adenocarcinoma), MCF7 (breast adenocarcinoma) and Saos2 (osteosarcoma). The results showed that, complex **264** is the most cytotoxic complex for PC3 [169]. A series of pyrazoles derivatives were synthesized and evaluated for their ALK5 inhibitory activity. Among them, compound **265** inhibited ALK5 phosphorylation with an IC_50_ value of 0.018 µM [170]. 

Li et al. synthesized a novel series of 4-pyrazolyl-1,8-naphthalimide derivatives and tested in vitro for their anticancer activity. Compound **266** showed improved cytotoxic activity with IC_50_ value of 0.51 µM against MCF-7 cells line [171]. Park et al. reported trisubstituted pyrazole-based ROS1 inhibitors. Among these compounds, compound **267** (IC_50_ = 13.6 nM) has exerted 5 fold potency than crizotinib and exhibited high degree of selectivity (selectivity score value = 0.028) representing the number of non-mutant kinases with biological activity over 90% at 10 µM [172]. A series of *1H*-pyrazolo[4,3-*d*]pyrimidin-7(6*H*)-ones were developed and evaluated in vitro for their anticancer activity against human cancer cell lines HeLa, CAKI-I, PC-3, MiaPaca-2, A549 gave good results. Compound **268** revealed that the compound shows anticancer activity through apoptosis mechanism and also inhibits mTOR with nonomolar potency [173]. 

A series of novel 5-phenyl-1*H*-pyrazole derivatives were synthesized by Wang et al. and evaluated for their antiproliferative as potential BRAFV600E inhibitors. Among them, compound **269** exhibited the most potent inhibitory activity with an IC_50_ value of 0.33 µM for BRAFV600E and has better antiproliferative activity against WM266.4 and A375 with IC_50_ value of 2.63 and 3.16 µM, respectively [174]. Yao et al. reported compound **270** to have appreciable inhibitory activity against class I and IIb HDAC and in vitro anticancer activities against several cancer cell lines [175]. 

**Figure 20:**
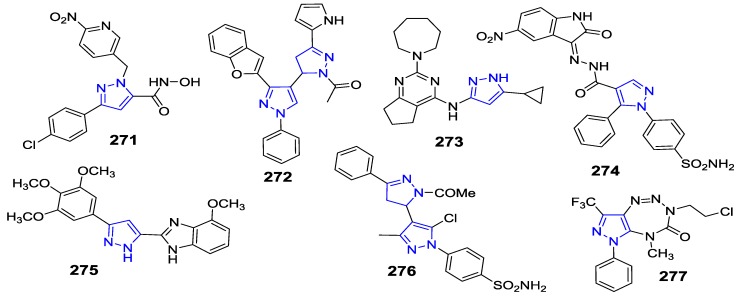
Some structures of pyrazoles with anticancer activity.

A series of novel pyrazolyl hydroxamic acid derivatives (Figure 20) were synthesized and investigated in vitro for their anticancer activities against human lung cancer cell line A549. The results showed that the compounds **271** (10 µM) exerted more effective anti-proliferation activity against A549 cell line [176]. Abd El-Karim et al. synthesized a series of novel benzofuran-pyrazole and evaluated for their in vitro antiproliferative activity. Compound **272** exhibited remarkable growth inhibitory activity pattern against leukemia CCRF-CEM, MOLT-4, lung cancer HOP-92, colon cancer HCC-2998, CNS cancer SNB-75, melanoma SK-MEL-2, ovarian cancer IGROV1, renal cancer 786-0, RXF 393, breast cancer HS 578T and T-47D (GI_50_: 1.00–2.71 µM) [177]. A series of novel 4-pyrazolecyclopentylpyrimidines were prepared and evaluated in vitro as IGF-1R tyrosine kinase inhibitors. Compound **273** was found to be most active, with an IC_50_ value of 10 nM [178]. Several new series of benzenesulfonamide derivatives incorporating pyrazole were prepared by Ibrahim et al. and screened for anti-tumor activity against the metalloenzyme carbonic anhydrase and human isoforms hCA I, II, IX and XII. The in vitro evaluation demonstrated compounds **274** was found to inhibit hCA XII with Ki of 3.7 nM [179]. New arylpyrazole linked benzimidazole conjugates were synthesized and evaluated for their ability to inhibit the growth of sixty cancer cell line panel. Compound **275** exhibited significant growth inhibitory activity against most of the cell lines ranging from 0.3 to 3 µM and expressed appreciable cytotoxic potential [180]. A series of pyrazolylpyrazolines were synthesized and evaluated for carbonic anhydrase inhibitory activity against cytosolic human isozymes. Compound **276** exhibited better inhibition profile against hCA II (Ki = 0.17 nM) [181]. 3-(2-chloroethyl)-5-methyl-6-phenyl-8- (trifluoromethyl)-5,6-[3,4-*f*][1,2,3,5]tetrazepin-4-(3*H*)-one **277** synthesized by Maggio et al. was tested antiproliferative activity. The compound exerted the maximum antiproliferative activity with GI_50_ value of 2.3 µM [182].

**Figure 21:**
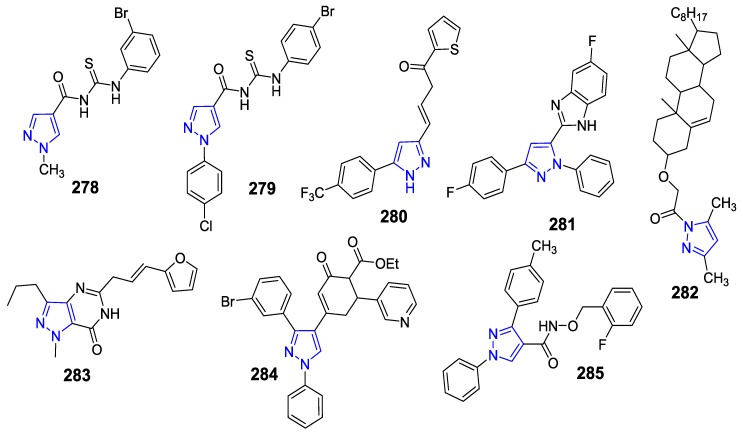
Pyrazole derivatives with anticancer activity.

Nitulescu et al. synthesized new pyrazole thiourea derivatives (Figure 21) and studied their apoptotic effects in human cancer cells. The results showed that the compound **278** exhibited the highest apoptosis-inducing effect [183]. The same author described the synthesis of new pyrazole derivatives as inhibitors of the cell cycle kinases. The compound **279** induced a significant increase of cells in G2/M phases in conjunction with an increased expression of cyclin A and cyclin B, emerging as a promising anticancer drug [184]. New pyrazole chalcones derivatives were described and assessed in vitro for their anticancer activity. The compound **280** was reported as potent anticancer agent with IC_50_ values of 18 and 47 µM against HeLa and MCF-7 cell lines, respectively [185]. A series of pyrazole derivatives containing benzimidazole moiety were synthesized and evaluated for their potential anti-proliferative activity against three human tumor cell lines. Compound **281** showed potent growth inhibition against A549, MCF-7, HeLa and HaCaT cell lines with IC_50_ values of 1.13, 0.95 and 1.57 µM, respectively [186]. Steroidal derivatives containing a pyrazole moiety were synthesized and evaluated for anticancer activity against a human leukemia cell line (HL-60). Compound **282** displayed promising behavior by showing better anticancer activity [187]. Shi et al. synthesized a series of pyrazole-carboxamide derivatives and evaluated for anticancer activity. Compound **283** exhibited strong inhibitory activity against MGC-803 cells, and showed the most potent telomerase inhibitory activity [188]. A series of pyrazoles derivatives synthesized by Alam et al. and evaluated for topoisomerase IIa inhibitory activity and in vitro cytotoxicity against a panel of cancerous cell lines (MCF-7, NCI-H460, HeLa) and a normal cell line (HEK-293T). Compound **284** showed superior cytotoxicity with an IC_50_ value of 7.01 µM for HeLa, 8.55 µM for NCI-H460 and 14.31 µM for MCF-7 cancer cell lines [189]. *N*-(Benzyloxy)-1,3-diphenyl-1*H*-pyrazole-4-carboxamide derivatives were synthesized and evaluated for anticancer activity as MEK inhibitors. Among these compounds, compound **285** showed the most potent inhibitory activity with IC_50_ of 91 nM for MEK1 and GI_50_ value of 0.26 μM for A549 cells [190].

**Figure 22:**
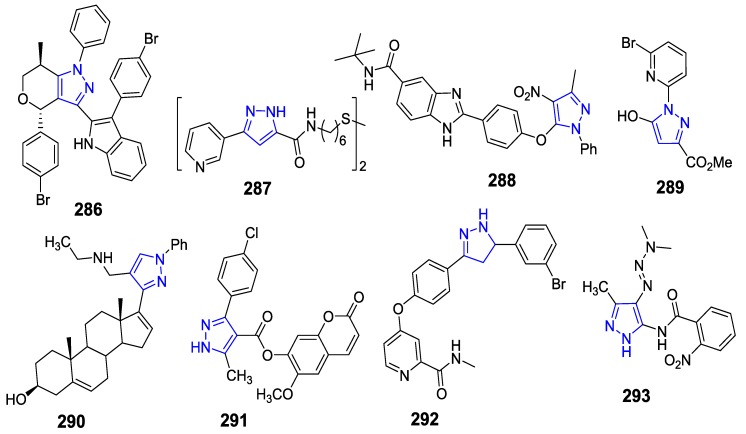
Pyrazole derivatives with anticancer activity.

A series of 1,4,6,7-tetrahydropyrano[4,3-*c*]pyrazole derivatives (Figure 22) were synthesized and tested in vitro for antitumor activity against four human cancer cell lines (MCF-7, EC-109, HGC-27, and PC-3). Compound **286** showed the most potent inhibitory activity against HGC-27 and PC-3 [191]. A new collection of *N*-(6-mercaptohexyl)-3-substituted-1*H*-pyrazole-5-carboxamide HDAC inhibitors was developed by Wen et al. The disulfide compound **287** was found to be potent cytotoxic agent against a panel of seven tumor cells, causing hyperacetylation of histone and non-histone proteins in cellular level, and demonstrated a notable in vivo anti-tumor activity in HCT-116 xeno-grafted model [192]. Pyrazole-benzimidazole derivatives are novel potent active Chk2 inhibitors were described by Galal et al. Out of the synthesized compounds, compound **288** was reported as the most potent effects as Chk2 inhibitors with cytotoxic properties besides their potentiation effects on the cytotoxicity of both cisplatin and doxorubicin, and showed marked antitumor activity as single agent in breast cancer–bearing animals [193]. A novel series of 1*H*-pyrazole-3-carboxylate derivatives were synthesized and screened for antitumor activity against BEL-7404, HepG2, NCI-H460, T-24, A549 tumor cell lines. Compound **289** exhibited lower IC_50_ value (129.75 µM) against HepG2 [194]. Li et al. synthesized novel steroidal pyrazole derivatives and evaluated for their cytotoxicity activity against 293T cell lines and three cancers cell lines: A549, Hela and MCF-7. Compound **290**, showed the highest potency, with an IC_50_ values of 0.87 µM and 0.53 µM for 293T cell lines and Hela cell lines, respectively [195]. A series of scopoletin-pyrazole hybrids were synthesized and their anticancer activities were evaluated in vitro against three human cancer cell lines including HCT-116, Hun7 and SW620. Results showed that the compound **291** exhibited potent cytotoxic activities with IC_50_ values below 20 µM [196]. A series of pyrazole derivatives bearing Sorafenib scaffold were synthesized by Wang et al. and evaluated for the cytotoxicity against A549, HepG2, MCF-7, and PC-3 cancer cell lines and some selected compounds were further evaluated for the activity against VEGFR-2/KDR, BRAF, CRAF, c-Met, EGFR and Flt-3 kinases. Compound **292** exhibited moderate to good activity toward c-Met and showed moderate to no activity against CRAF, EGFR, Flt-3 kinases, and showed strong antitumor activities against A549, HepG2 and MCF-7 cell lines with IC_50_ values of 2.84, 1.85 and 1.96 µM, respectively [197]. Several new 4-(3,3-dimethyltriazeno)-5-benzamidopyrazole derivatives were prepared and tested at 10 µM for their vitro anti-leukemic activity against K562 and Raji cell lines. The most active compound **293** showed growth inhibition values of 97.8% and 99.4% against the K562 and Raji cell lines, respectively [198].

### 3.3. Anti-Inflammatory and Analgesic Activity

**Figure 23:**
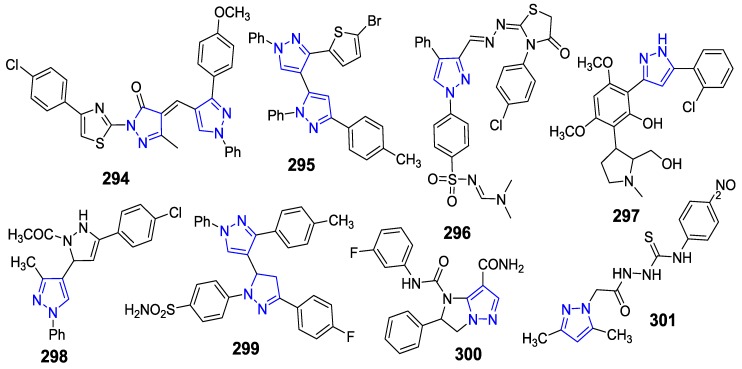
Pyrazole derivatives with anti-inflammatory activity.

A new group of pyrazole derivatives were designed (Figure 23) for evaluation as selective cyclooxygenase-2 (COX-2) inhibitors. Results indicated that the compound **294** exhibited significant COX-II inhibition (78.91±0.80 %) [199]. A series of 1*H*-pyrazolyl derivatives were described by Bekhit et al. and tested for their in vivo anti-inflammatory activity. Compound **295** exhibited anti-inflammatory activity comparable to that of indomethacin (LD_50_ > 500 mg/Kg), and showed good selective inhibitory activity against COX-2 enzyme [200]. Bekhit et al. synthesized a series of novel pyrazolyl benzenesulfonamide derivatives bearing thiazolyl ring and evaluated for anti-inflammatory activity. Among the synthesized compounds, compound **296** exhibited higher anti-inflammatory activity than the reference compound (indomethacin) [201]. 3,5-Diaryl pyrazole derivatives were prepared and evaluated for anti-inflammatory activity. Compound **297** was identified as the potent ant-inflammatory agent against TNF-α and IL-6 at 10 µM concentration [125]. Girisha et al. synthesized a novel series of 1-acetyl/propyl-3-aryl-5-(5-chloro-3-methyl-1- phenyl-1*H*-pyrazol-4-yl)-2-pyrazolines and screened for analgesic and anti-inflammatory activity. Compound **298** showed good activity comparable with that of standard drugs pentazocin and diclofenac sodium, respectively [202]. Sharma et al. synthesized and evaluated pyrazolyl-pyrazoline derivatives for their anti-inflammatory activity using carrageenan-induced rat paw edema assay. Amongst the tested compounds, **299** showed pronounced anti-inflammatory activity (32%) that was comparable to nimesulide (36%) [69]. Synthesis and anti-inflammatory evaluation of new 2,3-dihydro-imidazo[1,2-*b*]pyrazole derivatives were reported by Brullo et al., in which compound **300** showed an interesting dual activity inhibiting both fMLP-OMe and IL8-induced chemotaxis with IC_50_ values of 3.9 and 1.2 nM, respectively [203]. A novel series of pyrazoles containing benzenesulfonamides were synthesized by El-Moghazy et al., and evaluated in vivo for their anti-inflammatory activity. Compound **301** was found to be the most active anti-inflammatory agent (62.67% inhibition of edema) comparable to that of indomethacin (60.8% inhibition of edema) [204]. 

**Figure 24:**
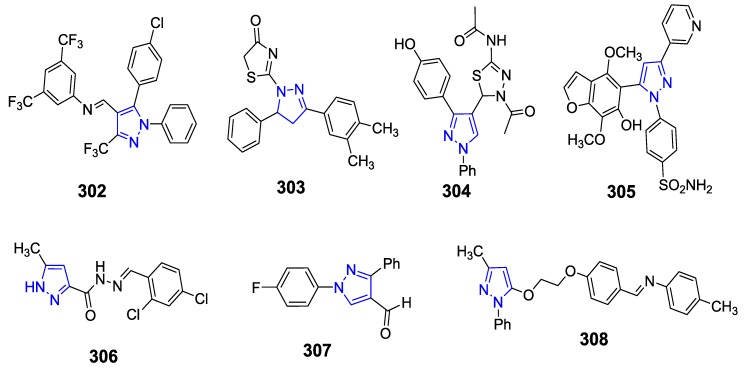
Pyrazole derivatives with anti-inflammatory and analgesic activities.

Pyrazole analogues (Figure 24) synthesized by Magda, et al., were evaluated in vitro for their ability to inhibit ovine COX-1/COX-2 isozymes. Among the tested compounds, compound **302** exhibited optimal COX-2 inhibitory potency (IC_50_ = 0.26 µM) and selectivity (SI = 192.3) comparable with reference drug celecoxib (IC_50_ value of 0.28 µM and selectivity index of 178.57) [205]. A series of dihydropyrazolyl-thiazolinone derivatives were synthesized and evaluated as potential cyclooxygenase-2 (COX-2) inhibitors. Among these compounds, compound **303** displayed the most potent COX-2 inhibitory activity with IC_50_ of 0.5 µM [206]. 1,3,4-Trisubstituted pyrazole derivatives were synthesized and screened for the anti-inflammatory activity by carrageenan-induced paw edema method. Compounds **304** showed excellent anti-inflammatory actvity (≥84.2% inhibition) compared to that of the standard drug diclofenac (86.72%) [207]. Novel series of celecoxib analogs were synthesized and evaluated for COX-1/COX-2 inhibitory activity and assessed for their anti-inflammatory activity and ulcerogenic liability in vivo. The 3-(pyridin-3-yl)pyrazole derivative **305** exhibited the highest anti-inflammatory activity and demonstrated about 40% reduction in ulcerogenic potential relative to the reference drug [208]. Karrouchi et al. synthesized pyrazole-hydrazone derivatives and evaluated for their anti-inflammatory activity. The anti-inflammatory activity of **306** at the dose of 100 mg/kg showed excellent protection against inflammation (92.59% inhibition) in comparison with Indomethacin [209]. A series of 1-(4-substituted-phenyl)-3-phenyl-1*H*-pyrazole-4-carbaldehydes were prepared and tested for their anti-inflammatory and analgesic activities. Among the prepared compounds, compound **307** exhibited the maximum anti-inflammatory and analgesic activities [210]. A novel series of pyrazole derivatives were synthesized by Tewari et al. and evaluated in vivo for their anti-inflammatory activity. Among all compounds, **308** showed comparable anti-inflammatory activity to nimesulide [211].

**Figure 25:**
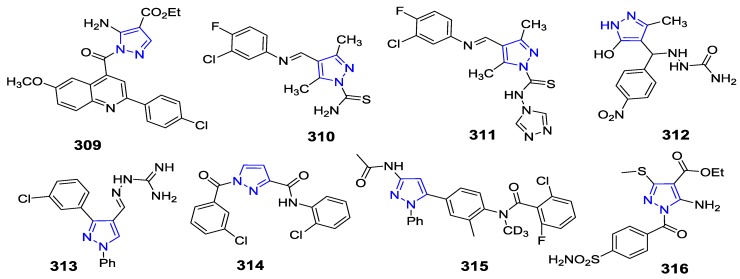
Pyrazole derivatives with anti-inflammatory and analgesic activities.

Several new pyrazole derivatives (Figure 25) containing a quinolone moiety were synthesized and tested for their anti-inflammatory and ulcerogenic effect. The most active compound **309** was found to be superior to celecoxib, and demonstrated the highest anti-inflammatory activity as well as the best binding profiles into the COX-2 binding site [212]. Hussain et al. synthesized pyrazole derivatives and investigated them for their, anti-inflammatory and analgesic activities. Results indicated that the compound **310** (80.29% inhibition) showed anti-inflammatory activity almost equal to that of the standard drug, ibuprofen (80.38%). Compound **311** showed moderate analgesic activity in comparison to their standard drugs [213]. Surendra Kumar et al. reported the synthesis and anti-inflammatory activity of pyrazole derivatives. The derivative **312** showed significant anti-inflammatory activity when compared to the standard drug diclofenc sodium [214]. A series of 1,3-diaryl pyrazole derivatives bearing aminoguanidine moiety were synthesized and evaluated for and anti-inflammatory activities. Compound **313** showed the greatest anti-inflammatory activity (93.59% inhibition), which was more potent than the reference drugs ibuprofen and indomethacin [215]. Compound **314** were identified by Pelcman et al., as the most potent inhibitors of human 15-lipoxygenase [216]. Wang et al. identified a series of novel pyrazole containing benzamides as potent RORγ inverse agonists. Compound **315** was found to be more potent, selective and have adequate profiles RORγ inverse agonists [217]. A series of novel ethyl-5-amino-3-methylthio-1*H*-pyrazole-4-carboxylates were synthesized and were screened for in vivo analgesic and anti-inflammatory activities. Compound **316** exhibited significant analgesic and anti-inflammatory activities at a dose of 25 mg/kg [218].

**Figure 26:**
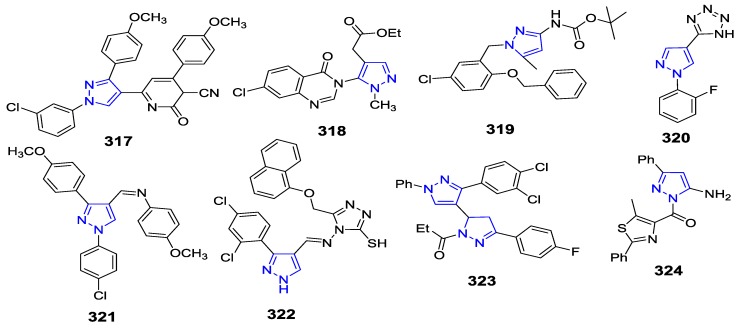
Pyrazole derivatives with anti-inflammatory and analgesic activities.

A new series of pyrazole-substituted were synthesized and investigated for their anti-inflammatory activity using the carrageenan-induced paw edema standard technique. Results revealed that, compound **317** seems to be the most effective prepared anti-inflammatory agent, revealing better activity (89.57% inhibition of edema) (Figure 26) [219]. Several new 1*H*-pyrazole-4-acetates containing quinazolinone rings were synthesized and screened for their analgesic and anti-inflammatory activities. Compound **318** showed appreciable anti-inflammatory and analgesic activities [220]. Compound **319** was identified by Hall et al. as brain penetrant compounds and both demonstrated efficacy in the CFA model of inflammatory pain [221]. De Moura et al. identified new pyrazole-containing tetrazole compound **320** as a non-steroidal anti-inflammatory drug [222]. Some novel 1,3,4-trisubstituted pyrazoles were synthesized by Ragab et al. and screened for their anti-inflammatory and analgesic activities. Compound **321** was found to be the most active one as anti-inflammatory and analgesic agents [223]. Vijesh et al. described the synthesis and analgesic activity of new 1,2,4-triazole and benzoxazole derivatives containing pyrazole moieties. The results revealed that the compound **322** showed significant analgesic activity [224]. A series of new substituted pyrazoline derivatives linked to a substituted pyrazole scaffold were prepared by Vieka et al. and screened for their anti-inflammatory and analgesic activities. The results revealed that the compound **323** could be identified as the most active member within this study with a dual anti-inflammatory and analgesic profile [225]. A series of novel 5-methyl-2-phenylthiazole-4-substituted-pyrazole derivatives were synthesized and evaluated for their anti-inflammatory and analgesic activities. Derivative **324** exhibited moderate to good anti-inflammatory and analgesic activities [226]. 

**Figure 27:**
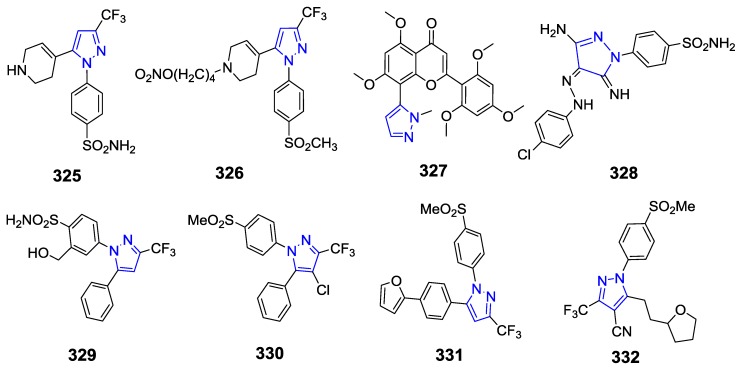
Pyrazole derivatives with anti-inflammatory activity.

A series of pyrazole derivatives were synthesized (Figure 27) by Chowdhury et al. All the compounds were evaluated for their anti-inflammatory potential using carrageenan-induced rat paw edema assay. Among the series compound **325** was found to possess potent anti-inflammatory potential (ED_50_ value 61.2 mg/kg) [227]. In order to improve anti-inflammatory profile some modified and novel nitric oxide releasing coxib prodrugs were synthesize. In contrary to previously described results, compound **326** exhibited higher oral anti-inflammatory potency [228]. A series of novel pyrazole amalgamated flavones were synthesized and tested for their in vitro COX inhibition and in vivo carrageenan induced hind paw edema in rats and acetic acid induced vascular permeability in mice. Among the synthesized compound **327** showed significant inhibitory profiles against COX-2, indicating that they are selective inhibitors for COX-2 [229]. Recently, novel, pyrazole-hydrazone derivatives as anti-inflammatory agents were synthesized and evaluated for their in vitro COX-1, COX-2 and 5-LOX enzymes inhibitition potential. Especially, Compound **328** (IC_50_ = 0.58 lM) showed better COX-2 inhibitory activity than celecoxib (IC_50_ = 0.87 lM) [230]. In search of novel anti-inflammatory motifs, several new pyrazole compounds were synthesized by Singh et al. and evaluated for cyclooxygenase inhibition against recombinant human COX-2 enzyme. Outcome of the study revealed that compound **329** having hydroxymethyl group ortho to sulfonamide group exhibited good inhibitory potency and selectivity toward COX-2 enzyme (SI = 297, COX-2 IC_50_ = 0.036 mM) [231]. In order to explore the canine selective COX-2 inhibitory potential of *N*-methanesulfonylpyridinyl-substituted trifluoro-methylpyrazole derivatives, several compounds have been screened for their canine selective COX-2 inhibitory activity using in vitro canine whole blood (CWB) COX inhibition assay. Among all the synthesized derivatives, compound **330** was found to be most potent COX-2 inhibitor with high selectivity index (IC_50_ = 0.06 mM, SI = 132) [232]. Cheng et al. synthesized some new *N*-methanesulfonyl pyridinyl-substituted pyrazole derivatives bearing heteroaryl moiety at 5-position of pyrazole and evaluated in vitro canine selective COX-2 inhibitory activity. Compound **331** is the most potent and selective canine COX-2 inhibitor (IC_50_ = 0.012 mM) which displayed COX-1/COX-2 selectivity ratio greater than 4000-fold and thus provided an excellent efficacy profile for the treatment of pain and inflammation [233]. Sakya and coworkers evaluated canine selective COX-2 inhibitory potential of some new substituted pyrazole derivatives. The results revealed that among prepared compounds, the compound **332** (COX-2 IC_50_ = 0.063 mM, SI = 262) was found to be most potent and COX-2 selective [234].

**Figure 28:**
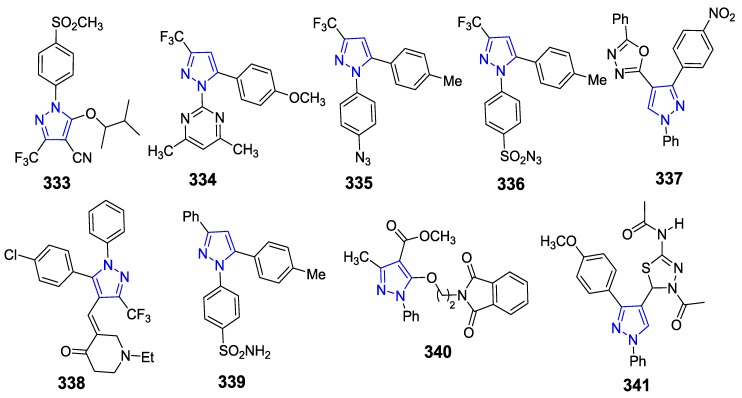
Pyrazole derivatives with anti-inflammatory activity.

Sakya et al. also evaluated in vitro canine selective COX-2 inhibitory activity of some new *N*-methanesulfonylpyridinyl-substituted pyrazole derivatives bearing ether/thioether substituents using CWB assay (Figure 28). The results revealed that among alkyl ethers, the compound **333** showed highest inhibitory potency and selectivity for COX-2 enzyme (IC_50_ = 0.09 mM, SI = 127) [235]. In a study, Aggarwal et al. explored the effect of 3/5-trifluoromethylpyrazole derivatives on in vivo anti-inflammatory potency at a dose level 50 mg/kg. Among all the tested compounds, **334** possessed very high anti-inflammatory potential 78% comparable to indomethacin (78%) after 3 h induction [236]. Knaus et al. synthesized and evaluated in vitro COX-1/COX-2 inhibitory potential of the celecoxib analogs having an azido group in place of the SO_2_NH_2_ moiety. The compound **335** was emerged as a selective COX-2 inhibitor [COX-1 IC_50_ > 100 µM, COX-2 IC_50_ = 1.5 µM, SI = 64] and displayed good anti-inflammatory properties [237]. In continuation to previous results, Knaus and coworkers have synthesized a series of celecoxib analogs and evaluated for their in vitro COX-1/COX-2 inhibitory activity with an expectation to find an efficient class of anti-inflammatory agents. Results revealed that compound **336** bearing SO_2_N_3_ group at para-position inhibited the activity of COX-1 enzyme selectively [COX-1 IC_50_ = 3.3 µM, COX-2 IC_50_ > 100, SI > 0.033] [238]. Some novel 2-phenyl-5-(1,3-diphenyl-1*H*-pyrazol-4-yl)-1,3,4-oxadiazoles derivatives have been synthesized for selective COX-2 inhibition with potent anti-inflammatory activity. Among all the tested compounds, compound **337** optimal COX-2 inhibitory potency (IC_50_ = 0.31 µM, ED_50_ = 74.3 mg/kg) [239]. Other 4-substituted novel trifluoromethylpyrazole derivatives have also been evaluated for their anti-inflammatory and COX-2 inhibitory potential. Results revealed that compound **338** displayed a promising degree of activity with ED_50_ value in the range 0.261 mmol/kg, in comparison to the standard drug, diclofenac (ED_50_ = 0.358 mmol/kg) and resulted 89.7% inhibition of edema at dose level of 200 mg/kg [240]. A series of pyrazolylbenzenesulfonamide derivatives were synthesized and evaluated for their anti-inflammatory and COX-1 and COX-2 inhibitory activities. Among investigated compounds, compound **339** was found to possess anti-inflammatory activity comparable to that of indomethacin and celecoxib, and exhibited COX-1/COX-2 selectivity [241]. Tewari et al. synthesized a series of pyrazole ester prodrugs analogues and evaluated in vitro for COX-2 inhibitory activity. The results indicated that compound **340** showed to possess maximum inhibitory effect when compared to control group [242]. A series of 1,3,4-trisubstituted pyrazole derivatives have been synthesized and evaluated for their cyclooxygenase (COX-1 and COX-2) inhibitory activity. Among these derivatives, compound **341** was the most potent and selective COX-2 inhibitor (IC_50_ = 1.33 μM), with a significant selectivity index (SI > 60) [243].

### 3.4. Anti-Tubercular Activity

**Figure 29:**
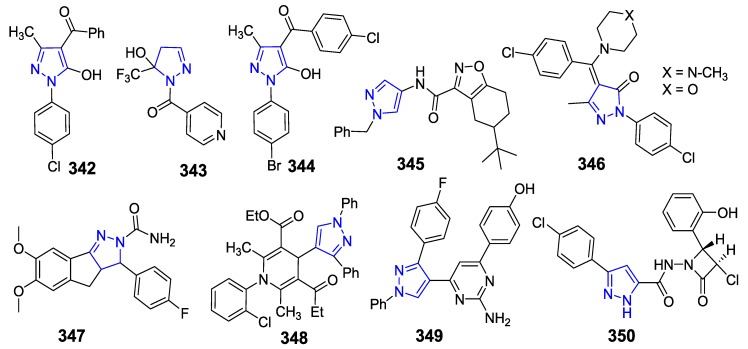
Pyrazole derivatives with anti-tubercular activity.

Manetti et al. identified new inhibitors of *Mycobacterium tuberculosis* (Figure 29). Compound **342** was found to be most active agent with a MIC value of 25 µg/mL [244]. A series of 3-substituted 5-hydroxy-5-trifluoro[chloro]methyl-1*H*-1-isonicotinoyl-4,5-dihydropyrazoles were synthesized by Almeida da Silva et al. and tested for their in vitro antimycrobial activity against *Mycobacterium tuberculosis H37Rv*, INH-resistant clinical *M. tuberculosis* isolates non-tuberculous mycobacteria. Amongst the synthesized compounds, compound **343** was found to be the most active agents against susceptible *M. tuberculosis* and several INH-resistant strains [245]. As a continuation of our previous work that turned toward the identification of antimycobacterial compounds with innovative structures, two series of pyrazole derivatives were synthesized by Castagnolo et al. and assayed as inhibitors of *M. tuberculosis* H37Rv. The pyrazole derivative **344**, with the *p*-bromophenyl group at the N1 position, was showed to be very active (MIC = 4 µg/mL) [246]. Velaparthi et al. presented a series of 5-*tert*-butyl-*N*-pyrazol-4-yl-4,5,6,7-tetrahydrobenzo[*d*]isoxazole-3-carboxamide derivatives as novel potent *M. tuberculosis* pantothenate synthestase inhibitors. The new compound **345** exhibited the maximum activity with IC_50_ of 90 nM [247]. Castagnolo et al. synthesized two series of novel rigid pyrazolone derivatives and evaluated as inhibitors of *M. tuberculosis*, the causative agent of tuberculosis. The results showed that compound **346** bearing *N*-Me-piperazine and morpholine moieties proved to be very active with MIC = 4 µg/mL [248]. A series of 3a,4-dihydro-3*H*-indeno[1,2-*c*]pyrazole-2-carboxamide analogues were synthesized and evaluated for antitubercular activity. The compound **347** was found to be the most promising compound active against *M. tuberculosis H37Rv* and isoniazid resistant *M. tuberculosis* with MIC concentration 3.12 µM and 6.25 µM, respectively [249]. A new *N*-aryl-1,4-dihydropyridines derivatives bearing 1*H*-pyrazole ring were synthesized and evaluated for antitubercular activity. The lowest MIC value 0.02 µg/mL, was found for compound **348** making it more potent than first line antitubercular drug isoniazid [250]. As a part of our ongoing research to develop novel antitubercular agents, a series of *N*-phenyl-3-(4-fluorophenyl)-4-substituted pyrazoles have been synthesized and tested for antimycobacterial activity in vitro against *M. tuberculosis* H37Rv. Amongst them, compound **349** displayed the most potency with IC_50_ of 0.47 µM [251]. As a part to develop novel antitubercular agent, a series of 3-(4-chlorophenyl)-4-substituted pyrazoles have been synthesized by Pathak et al. and tested for antitubercular activity in vitro against *M. tuberculosis* H37Rv strain. Among them tested, compound **350** showed excellent antitubercular activity with MIC of 0.35 mg/mL [76]. 

**Figure 30:**
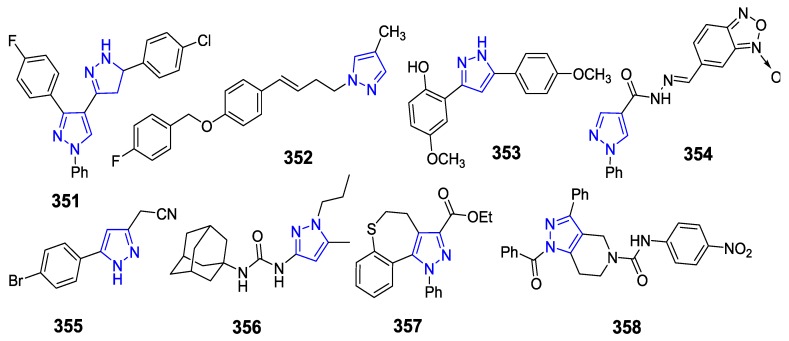
Pyrazole derivatives with anti-tubercular activity.

A new series of fluorinated pyrazoles (Figure 30) were synthesized and screened for their in vitro anti-tubercular activities against *M. tuberculosis* H37Rv. Results showed that pyrazoline **351** displayed significant anti-tubercular activities against the *M. tuberculosis* H37Rv strain (MIC = 6.25 µg/mL) [252]. A series of 1-[(4-benzyloxyphenyl)-but-3-enyl]-1*H*-azoles has been identified by Anand et al. as potent antitubercular agents against *M. tuberculosis*. Compound **352** exhibited significant antitubercular activities with MIC value 0.61 µg/mL, comparable to many standard drugs [253]. Fullam et al. described the synthesis and inhibitory potencies of a novel series of 3,5-diaryl-1*H*-pyrazoles as specific inhibitors of prokaryotic arylamine *N*-acetyltransferase enzymes. Compound **353** was found to have good anti-mycobacterial activity and inhibited the growth of both *M. tuberculosis* with an MIC < 10 µg/mL (34 µM) [254]. Hernández et al. reported the preparation and anti-tubercular activity on *M. tuberculosis* H37Rv of hybrid furoxanyl *N*-acylhydrazones bearing pyrazole moiety. Among them, compound **354** displayed good selectivity against *M. tuberculosis* with MIC value 17.9 µM [255]. Various substituted pyrazole derivatives have been synthesized and evaluated for their in vitro anti-tubercular activity against *M. tuberculosis* H37Rv strain. Compound **355** exhibited significant anti-tubercular activity at MIC value 25 µM concentration [256]. A new series of 1-adamantyl-3-heteroaryl ureas containing pyrazole ring were synthesized by North et al. and evaluated for their anti-mycobacterial activity against MTB H37Rv. Among the synthesized compounds, compound **356** exhibited excellent activity against MTB H37Rv with MIC value 1.56 µg/mL [257]. A series of pyrazole derivatives containing thiochromeno and benzothiepino moieties were prepared and evaluated for antituberculosis activity. The compound **357** showed moderate inhibitory activity against MTB at MIC 14 µM [258]. Samala and coworkers have reported the synthesis and evaluation of the bioactivity of 3-phenyl-4,5,6,7-tetrahydro-1*H*-pyrazolo[4,3-*c*]pyridine derivatives against M. tuberculosis (MTB) pantothenate synthetase Trypanosoma. Among the compounds, **358** was found to be the most active compound with IC_50_ of 21.8 µM against MTB PS [259].

**Figure 31:**
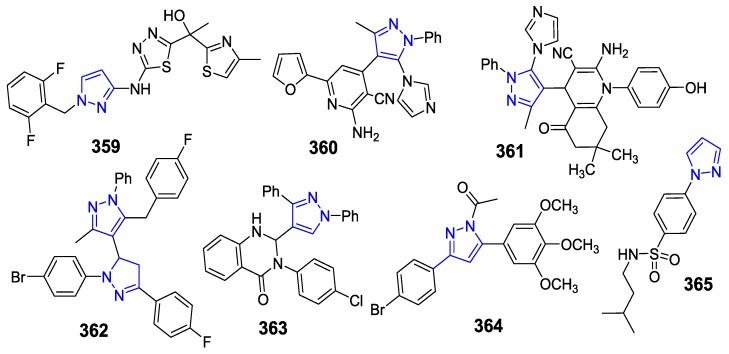
Pyrazole derivatives with anti-tubercular activity.

The design, synthesis and anti-mycobacterial activities of pyrazole bearing a methylthiazole scaffold (Figure 31) were reported by Shirude et al. Among the synthesized compounds, **359** showed similar enzyme inhibition against *M. tuberculosis* with a MIC value of 0.5 μM [260]. Novel 5-imidazopyrazoles incorporating 2-amino-3-cyano pyridine derivatives were synthesized and tested for their in vitro, anti-tuberculosis activity. Compound **360** displayed excellent inhibition (96% at 25 μg/mL) against anti-tubercular activity as compared to standard drugs [87]. A new category of polyhydroquinoline derivatives were synthesized were evaluated for their in vitro anti-tubercular activity against *M. tuberculosis* H37Rv strain. Compound **361** exhibited moderate anti-tuberculosis activity compared with the first line drugs. The outcome of the result revealed that, compound **361** was found to possess excellent activity (94% at 250 mg/mL and 100 mg/mL) against *M. tuberculosis* H37Rv [261]. Karad et al. reported the synthesized and anti-tuberculosis activity against *M. tuberculosis* H37Rv of a novel series of fluoro-substituted pyrazolylpyrazolines. Good anti-tubercular activity was exhibited by compound **362** (96% at 250 mg/mL) [262]. Mehta et al. synthesized a new series of quinazolin-4(3*H*)-one derivatives containing a (1,3-diphenyl-1*H*-pyrazol-4-yl) core and screened for their anti-tubercular activities. Compound **363** was reported as the most active compound with MIC of 12.5 µg/mL against MTB H37Rv strain [91]. Various substituted 1-(3,5-diaryl-4,5-dihydro-1*H*-pyrazol-1-yl)ethanone derivatives were synthesized by Pathak et al. and evaluated for their in vitro anti-tubercular activity against *M. tuberculosis* H37Rv strain. Compound **364** exhibited significant anti-tubercular activity at MIC value 12.5 µM concentration [263]. The design, synthesis, and in vitro anti-tubercular activity of compound **365** were reported by Villemagne and coworkers. The compound displayed moderate anti-tubercular activity (EC_50_ > 10 µM) [264].

**Figure 32:**
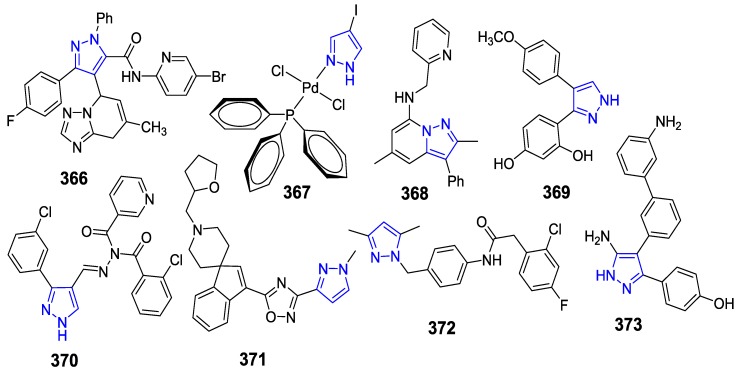
Pyrazole derivatives with anti-tubercular activity.

A series of novel pyrazole linked triazolo-pyrimidine hybrids (Figure 32) were synthesized and evaluated for their anti-tuberculosis activity against *M. tuberculosis* H37Rv strain. Compound **366** inhibited *M. tuberculosis* (99%) at MIC 0.39 µg/mL [265]. A series pyrazolyl-based Pd(II) complexes were synthesized by Da Silva et al. and evaluated in vitro for antimycobacterial activity. Anti-tuberculosis evaluation demonstrated that compound **367** displayed excellent activity with MIC of 7.61 µM [266]. A novel aminopyrazolo[1,5-*a*]pyrimidine derivatives were synthesis by Street et al. and tested for their anti-mycobacterial activity. Among the prepared compounds, compound **368** displayed promising anti-mycobacterial activity (MIC_99_ = 5 µM) [267]. Mutai et al. reported the synthesis and antimycobacterial activity of formononetin analogues bearing pyrazole ring. When all compounds were tested at a concentration of 10 µM, the pyrazole linked compound **369**, exhibited 40% inhibition of the H37Rv strain of *M. tuberculosis* [268]. The design, synthesis and evaluation of anti-tubercular activity of new INH-pyrazole analogs were reported by Nayak et al. the in vitro anti-mycobacterial evaluation demonstrated that compound **370** emerged as promising anti-tubercular agents with MIC of 0.8 µg/mL which is much lower than the MIC of the first line anti-tubercular drug, ethambutol [269]. Several 1,2,4-oxadiazole/pyrazole derivatives have been screened for anti-tubercular activity, and most of them showed weak to moderate activity. Amongst them, 1,2,4-oxadiazole pyrazole **371** displayed moderate potency against *M. tuberculosis* H37Rv strain [270]. A series of *N*-benzyl-4-((heteroaryl)methyl)-benzamides as a novel class of direct InhA inhibitors by high-throughput screening were identified by Guardia et al. These compounds displayed potent activity against MTB (MIC_90_: 6 to 125 µM), maintaining activity versus *KatG* mutant clinical strains (IC_50_: 12–31 µM) and emerging as a potential tool against MDR-TB and XDR-TB. The pyrazole derivative **372** (IC_50_ = 0.04 µM) is a potent direct InhA inhibitor with moderate whole-cell activity and an encouraging safety profile, but unfortunately it was not efficacious in an in vivo murine model of TB infection [271]. Using a fragment-based approach, a novel series of potent and isoform selective inhibitors of the essential MTB enzyme CYP121 have been developed by Kavanagh et al. The good selectivity of CYP121 inhibitors, particularly compound **373**, demonstrated here against human P450s, is promising for the development of this series of CYP121 inhibitors [272].

**Figure 33:**
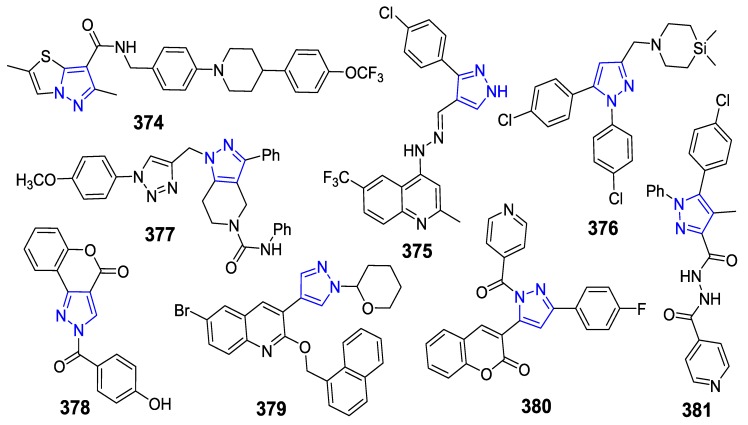
Pyrazole derivatives with anti-tubercular activity.

A series of biheterocyclic (1*H*-indole, benzofuran, pyrazolo[1,5-*a*]pyrimidine, pyrazolo[1,5-*a*]pyrimidin-5-4*H*)-one, imidazo[2,1-*b*]thiazole and pyrazolo[5,1-*b*]thiazole) derivatives (Figure 33) were synthesized and evaluated for their anti-tubercular activities. The imidazo[2,1-*b*]thiazoles and pyrazolo[5,1-*b*]thiazoles exhibited promising anti-tubercular activity in varying degrees. Especially, the 2,6-dimethylpyrazolo[5,1-*b*]thiazole **374** exhibited strong suppressing function against H37Ra strain with MIC value of 0.03 µg/mL [273]. Nayak et al. reported the synthesis and anti-tubercular activity of some active fluorine containing quinoline-pyrazole hybrid derivatives. Among the synthesized compounds, derivatives **375** emerged as active anti-TB leads which exhibited low toxicity profile and high selectivity index value [274]. A novel sila analogues of Rimonabant as potent anti-tubercular agents were identified by Ramesh et al. the sila analogue **376** was found to be the most potent anti-mycobacterial compound with MIC = 31 nM from this series with an excellent selectivity index [275]. Various 1-((1-(substituted)-1*H*-1,2,3-triazol-4-yl) methyl)-*N*,3-diphenyl-6,7-dihydro-1*H*-pyrazolo[4,3-*c*]pyridine-5(4*H*)-carboxamides were prepared and screened for in vitro anti-tubercular activity against *M. tuberculosis* H37Rv strain. Among the compounds, **377** was found to be the most active compound with IC_50_ 1.01 µM against MTB PS; it inhibited MTB with MIC 24.72 µM [276]. A new 2-aroyl-[1]benzopyrano[4,3-*c*]pyrazol-4(1*H*)-one derivatives containing hydrazide-hydrazone analogues were prepared and tested in vitro for their anti-mycobacterial activity against reference strain *M. tuberculosis* H37Rv. Compound **378** demonstrated significant MIC value 0.32 µM, which was comparable to those of isoniazid [277]. A new bedaquiline derivatives containing a pyrazole moiety were identified by He et al. and tested for their inhibitory activities against ATP synthesis inhibition in mycobacteria. The results showed that compound **379** inhibited ATP synthesis with IC_50_ > 62.9 µM [278]. Various pyrazole derivatives derived from isoniazide pharmacophore along with coumarin scaffold were investigated for their anti-mycobacterial activity against MTB H37Rv by a resazurin MIC assay. Amongst them, compound **380** showed excellent activity at MIC 0.625 µg/mL and exhibited 80% growth inhibition of MTB H37Rv [279]. A series of pyrazole derivatives were synthesized by Sriram et al. and subjected to in vitro screening against MTB H37Rv. The most potent analogue **381** exhibits MIC value of 3.13 µg/mL compared to 3.25 and 50 µg/mL for ethambutol and pyrazinamide [280].

**Figure 34:**
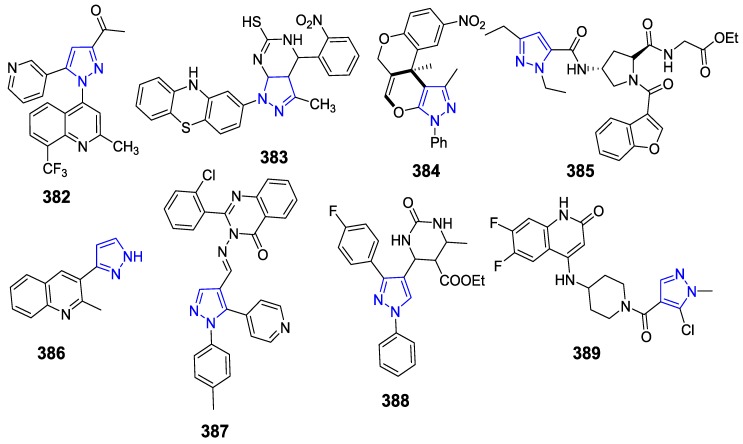
Pyrazole derivatives with anti-tubercular activity.

The design, synthesis, and in vitro anti-tubercular activity of a new series of 8-trifluoromethyl quinoline substituted pyrazole-3-carboxamides (Figure 34) were described by Nayak et al., Among the tested compounds, compound **382** showed significant inhibition activity against *M. tuberculosis* H37Rv strain with MIC of 3.13 μg/mL, which is comparable with the activity of standard drug, ethambutol [281]. A series of phenothiazine clubbed pyrazolo[3,4-*d*]pyrimidines were synthesized by Trivedi et al. and their ability to inhibit growth of *M. *tuberculosis** in vitro have been determined. The results showed that compound **383** exhibited excellent anti-tubercular activity with percentage inhibition of 96%, at a MIC < 6.25 μg/mL [282]. Labana et al. described the synthesis and anti-tubercular activity of benzopyran-annulated pyrano[2,3-*c*]pyrazoles derivatives. Results indicated that compound **384** showed excellent anti-tubercular activity with a percent inhibition growth around 93% [283]. According to the research by Encinas et al., benzofuran pyrazole derivatives showed considerable in vitro activity against MTB H37Rv, and compound **385** showed the most potency with MIC_90_ of 0.05 µM [284]. A series of quinolinyl heterocycles were evaluated for their anti-mycobacterial activity against *M. smegmatis*, and some quinolinyl pyrazole hybrids showed excellent anti-mycobacterial activity such as **386** (MIC = 14.66 µg/mL) was as potent as isoniazide (MIC = 12.07 µg/mL) [285].The in vitro abilities of quinazolinone pyrazole derivatives to inhibit growth of MTB H37Rv have been reported by Pandit et al. The results exhibited all hybrids showed considerable anti-TB activity, particularly, hybrid **387** (96%, MIC_90_ < 3.125 µg/mL) warrant further investigation [286]. Dihydropyrimidine pyrazole derivatives were synthesized and evaluated for their in vitro anti-tubercular activity against MTB H37Rv, compound **388** were found to be the most active compounds in vitro with MIC of 0.02 µg/mL, with the highest SI > 500 were more potent than INH (0.03 µg/mL) [287]. A series of 4-aminoquinolone piperidine amides were synthesized by Naik et al. and evaluated for their anti-tubercular activity against non-replicating phase (NRP) and drug-resistant strains of MTB. The most active **389** (MIC of 0.4–50 µM) showed promising activity against MTB H37Rv, DprE1 overexpressed (OE), InhA OE, TopA OE, PimA OE, BTZ043 (C387S), TMC207-resistant mutant clone 8.1 and moxifloxacin-resistant mutant clone 4.1 strains [288]. 

### 3.5. Anti-Viral Activity

**Figure 35:**
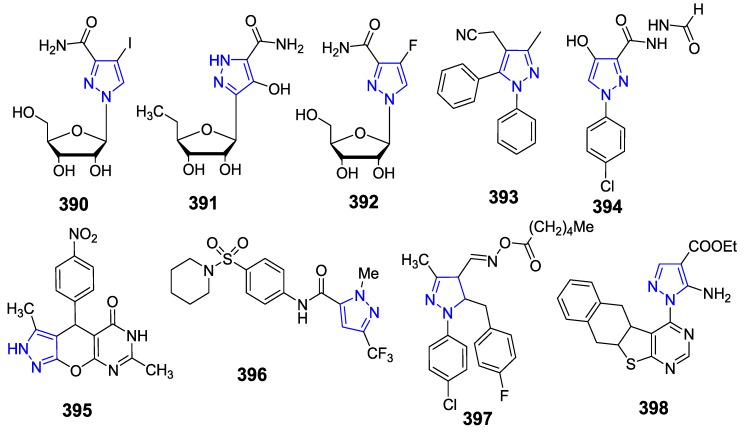
Pyrazole derivatives with anti-viral activity.

Several pyrazole and pyrazolo[4,3-*d*]-1,2,3-triazine-4-one ribonucleozides (Figure 35) were prepared by Manfredini et al. and tested in vitro for antiviral activities against herpes simplex type 1 (HSV-1), africain swine fever (ASFV), polio, coxsackie, vescicular stomatitis virus (VSV), and HIV-1. Among pyrazole nucleosides, compound **390** showed a selective and inhibited the HIV-1 multiplication in acutely infected C8166 cells [289]. In searching for derivatives of pyrazofurin that could display antiviral properties by means that do not require 5′-deoxypyrazofurin derivatives has been synthesized by Chen and Schneller and evaluated for their antiviral activity against a large number of viruses including herpes-, pox-, myxo-, toga-, arena-, rhabdo-, picorna-, reo-, and retroviruses. Compound **391** proved active against respiratory syncytial virus (in HeLa cells), vaccinia virus (in embryonic skin-muscle fibroblast cells), vesicular stomatitis virus (in HeLa cells), and influenza A virus (in Madin-Darby canine kidney cells) at concentrations ranging from 4 to 20 µg/mL [290]. A novel fluoropyrazole ribonucleoside has been synthesized and evaluated in vitro for their anti-influenza activity. The fluoropyrazole nucleoside **392** was found to have excellent activity against influenza A and B in vitro with I_50_ values of 0.2 and 0.4 µg/mL, respectively [291]. Genin et al. discovered a novel 1,5-diphenylpyrazole class of HIV-1 nonnucleoside reverse transcriptase inhibitors (NNRTIs). Compound **393** was found to have good activity versus wild-type (IC_50_ = 2.3 µM) and delavirdine-resistant P236L (IC_50_ = 1.1 µM) reverse transcriptase (RT) [292]. Anovel series of 1-(4-chlorophenyl)-4-hydroxy-1*H*-pyrazole-3-carboxylic acid hydrazide analogs has been synthesized and were investigated for their in vitro effect on the replication of hepatitis-C virus (HCV) in HepG2 hepatocellular carcinoma cell line infected with the virus using the reverse transcription-polymerase chain reaction (RT-PCR) technique. The results revealed that compounds **394** were capable of inhibiting the replication of both the HCV RNA (+) and (−) strands at 10–100 µg/mL concentration range [293]. Some novel pyrazolo[4′,3′:5,6]pyrano[2,3-*d*]pyrimidine derivatives were prepared and tested for antiviral activity against Herpes Simplex Virus type-1 (HSV-1). The obtained results revealed that the pyrazolopyranopyrimidine **395** showed the highest effect on HSV-1 than the other tested compounds, where its antiviral activity increased from 63% at concentration of 20 µg/10^5^ cells to 95% at concentration of 40 µg/10^5^ cells [294]. Sun et al. identified 1-methyl-3-(trifluoromethyl)-*N*-[4-(pyrrolidinylsulfonyl)phenyl]-1*H*-pyrazole-5-carboxamide as a novel and potent inhibitor against multiple primary isolates of diverse measles virus (MV) genotypes currently circulating worldwide. The most active piperidine derivative **396**, when subjected to a secondary virus titer reduction assay, revealed activity against live MV (0.012 ± 0.017 µM, strain Alaska) and no cytotoxicity [295]. A novel series of pyrazolaldoxime ester derivatives were synthesized by Ouyang et al. and evaluated for their antiviral activities against TMV. The results of bioassay showed that these title compounds exhibited weak to good anti-TMV bioactivity. Title compound **397** showed better biological activity and exhibited a higher affinity for TMV CP [296]. Some novel substituted pyrazole and pyrazolo[3,4-*d*]pyrimidine derivatives 2, 4, 8, and 9 were synthesized and tested for their antiviral activity against hepatitis-A virus (HAV) and herpes simplex virus type-1 (HSV-1). Compound **398** revealed the highest anti-HAV activity at a concentration of 20 µg/10^5^ cells, in comparison with the other tested compounds [297].

**Figure 36:**
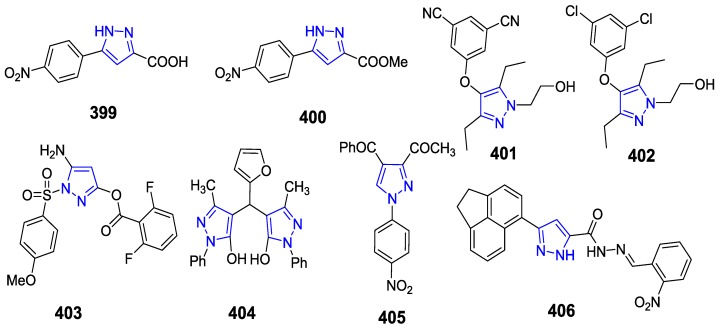
Pyrazole derivatives with anti-viral activity.

Zeng et al. have been synthesized a novel phenyl-substituted 1*H*-pyrazole-3-carboxylic acids (Figure 36) and were conveniently examined with respect to the effect on the IN inhibition and HIV replication. The best antiviral effect was exhibited by 5-(4-nitrophenyl)-1*H*-pyrazole-3-carboxylic acid **399** and 3-(3-(benzyloxy)phenyl)isoxazole-5-carboxylic acid **400** with an EC_50_ value of 3.6 and 253 µM [298]. A new series of *N*-hydroxyethyl pyrazoles derivatives were prepared by Mowbray et al. and evaluated in vivo for their anti-HIV activity. Compound **401** demonstrated excellent activities against large panels of wild type and drug-resistant HIV consistent with the encouraging profile demonstrated against the isolated RT enzymes shown above [299]. In another study, the same authors described the design and synthesis of a novel series of non-nucleoside HIV reverse transcriptase inhibitors (NNR-TIs) based on a pyrazole template. The compounds are active against wild type reverse transcriptase (RT) and retain activity against clinically important mutants. Combining the best 3- and 5-substituents gave the 3,5-diethylpyrazole **402** as the most potent compound in this early series [300]. Sidique et al. described the design and synthesis of 3-substituted pyrazole ester derivatives which are active as allosteric inhibitors of West Nile Virus NS2B-NS3 proteinase. Compound **403** was found to be more promising in comparison to the other one, with the IC_50_ value 1.96 µM [301]. 4,4′-(Arylmethylene)bis(1*H*-pyrazol-5-ol) derivatives has been synthesized by Sujatha et al. and evaluated for in vitro antiviral activity against peste des petits ruminant virus (PPRV). Compound **404** emerged as the most interesting compound in this series exhibiting excellent antiviral activity against PPRV and found to be more potent than the standard drug ribavirin used [302]. Three series of novel pyrazole derivatives were synthesized by Riyadh et al. and tested for anti-viral activity against HCV. Compound **405** was proved to be a notable anti-HCV activity with MIC value of 0.144 µg/mL [303]. Shih et al. identified novel pyrazole compound with potent and selective anti-influenza virus activity using a similar cell-based neutralization (inhibition of virus-induced cytopathic effect) assay. After screening 20,800 randomly selected compounds from a library, we found that **406** has potent inhibitory activity [304]. 

**Figure 37:**
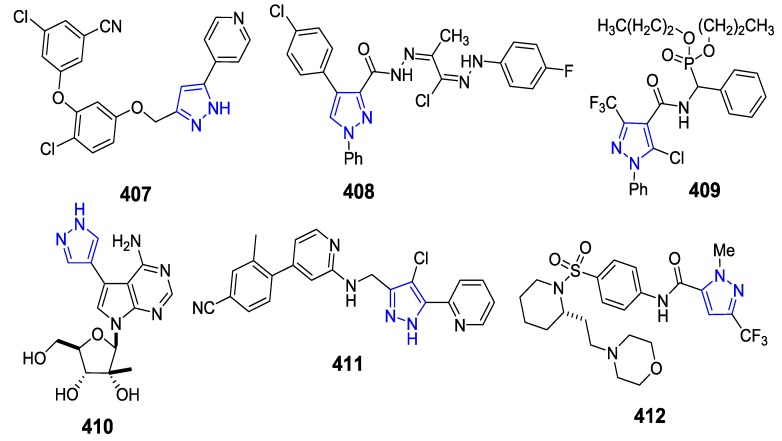
Pyrazole derivatives with anti-viral activity.

Su et al. reported a series of novel pyrazole derivatives (Figure 37) as potent inhibitors of HIV-1 RT with nanomolar intrinsic activity on the WT and key mutant enzymes and potent antiviral activity in infected cells. Compound **407** showed excellent intrinsic antiviral activity against WT, K103N, and Y181C mutants, and exhibited excellent activity in the cell base assay against the same panel of mutants with small shift in activity between 10% FBS and 50% NHS [305]. Several new pyrazole- and isoxazole-based heterocycles were synthesized by Dawood et al. and screened for their inti-viral activity against Herpes simplex type-1 (HSV-1). Among the tested compounds, compound **408** showed the highest activity and reduced the number of HSV-1 plaques by 69% [306]. A series of novel pyrazole amides containing α-aminophosphonate moiety were synthesized by Wu et al. and evaluated for their antiviral activity. The title compound **409** showed some curative activities (50.1%) against tobacco mosaic virus at 0.5 mg/mL [307]. A novel series of potent nucleoside inhibitors of Hepatitis C virus (HCV) NS5B polymerase bearing pyrazole ring were reported by Di Francesco et al. Amongst these, the pyrazole analog **410** showed significantly improved intrinsic potency, both inhibiting NS5B polymerase with NTP IC_50_ = 0.5 µM and displayed interesting levels of anti-viral activity in the replicon assay, with EC_50_ = 7.8 µM [308]. Kim et al. identified a novel class of aryl substituted pyrazole compounds as potent non-nucleoside reverse transcriptase inhibitors (NNRTIs) for anti-human immunodeficiency virus (HIV) activity using a cell-based full replication assay. The optimization of the antiviral activity leading to the discovery of compound **411** which possessed excellent potency against wild-type HIV-1 (EC_50_ = 0.2 nM) as well as viruses *bearing Y181C* and *K103N* resistance mutations in the reverse transcriptase gene [309]. Ndungu et al. the synthesis and a SAR strategy that led to the discovery of novel pyrazole derivatives as potent measles virus (MeV). Optimization of in vitro potency and aqueous solubility led to the discovery of pyrazole **412**, a potent inhibitor of MeV (EC_50_ = 60 nM) with an aqueous solubility of approximately 60 μg/mL [310].

**Figure 38:**
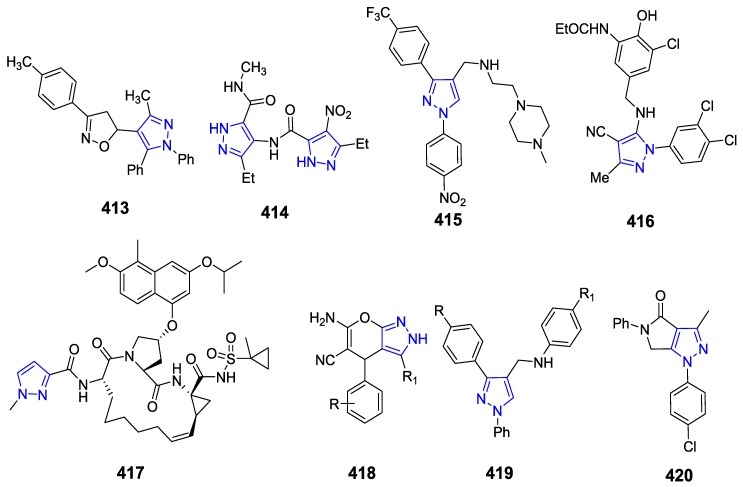
Pyrazole derivatives with anti-viral activity.

A new series of 4-substituted 3-methyl-1,5-diphenyl-1*H*-pyrazoles (Figure 38) has been synthesized by Tantawy et al. and evaluated in vitro for antiviral activity against herpes simplex virus type-1 grown on Vero African green monkey kidney cells through plaque-reduction assay method using acyclovir as a positive control. The results of the antiviral activity of the prepared compounds showed that compound **413** exhibited strong antiviral activity with IC_50_ value of 0.02 compared to the used reference drug [311]. A novel series of bis-pyrazole compounds were synthesized by Zhang et al. and tested for anti-viral activity against tobacco mosaic virus (TMV). Compound **414** showed higher activity superior to ningnanmycin at a concentration of 0.5 µg/mL and equal activity at 0.1 µg/mL [312]. Hwang et al. identified a series of 1,3,4-trisubstituted pyrazoles as potent hepatitis C virus (HCV) inhibitor by our phenotypic high-throughput screening using infectious HCVcc. Among them compound **415** was the most potent compound with EC_50_ value of 0.11 µM [313]. Mizuhara et al. synthesized a series of phenylpyrazole derivatives for the development of novel anti-HIV agents. Among the synthesized compounds, the 3,4-dichloro derivative **416** also exhibited more potent anti-HIV activity when (EC_50_ = 0.047 µM) [314]. Given the emergence of resistance observed for the current clinical-stage hepatitis C virus (HCV) NS3 protease inhibitors, there is a need for new inhibitors with a higher barrier to resistance. Moreau et al. reported a rational approach to the discovery of macrocyclic acylsulfonamides bearing pyrazole moiety as HCV protease inhibitors addressing potency against clinically relevant resistant variants. Compound **417** displayed most remarkable antiviral activity with an EC_50_ values of 0.14 and 1.0 nM against mutant D168V 1b and R155K 1a, respectively [315]. A series 24 compounds of diarylaniline analogues bearing pyrazole scaffold as non-nucleoside reverse transcriptase inhibitors (NNRTIs) were developed by Bhadoriya et al. using 3D-QSAR and pharmacophore modelling of *NNRTIs*. The survived 12 hits show new scaffolds 2,4-dihydropyrano[2,3-*c*]pyrazole **418** for anti-HIV-1 chemotherapy as NNRTIs [316]. A series of *N*-((1,3-diphenyl-1*H*-pyrazol-4-yl)methyl)anilines were synthesized by Fioravanti et al. and evaluated in vitro for cytotoxicity and antiviral activity against a large panel of viruses. Most of the tested compounds **419** interfered with RSV replication in the micromolar concentrations (EC_50_s ranging from 5 μM to 28 μM) [317]. A series of novel pyrazole fused heterocyclic derivatives were synthesized and evaluated for their catalytic DNA cleavage abilities and anti-BVDV activities. Among them, compound **420** showed the highest antiviral activity (EC_50_ = 0.12 mmol/L) and was 10 fold more than that of the positive control ribavirin (EC_50_ = 1.3 mmol/L) [318].

**Figure 39:**
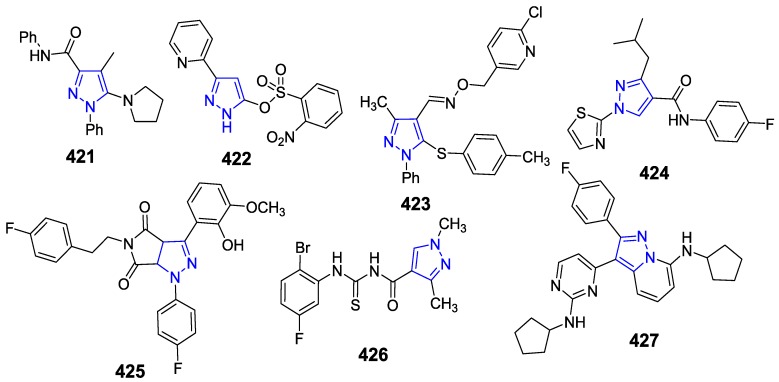
Pyrazole derivatives with anti-viral activity.

Manvar et al. reported the synthesis and mechanism of inhibition of pyrazolecarboxamide derivatives (Figure 39) as a new class of HCV inhibitors. Compound **421** exhibited an EC_50_ of 6.7 µM and selectivity index of 23 against HCV 1b, and reduced the RNA copies of the infectious Jc1 chimeric 2a clone by 82% at 7 µM [319]. A new series of pyridine-pyrazole-sulfonate compounds were synthesized evaluated for their anti-HBV activities and established the structure-activity relationship (SAR) in HepG2 2.2.15 cells. Among these compounds, compound **422** showed the most potent inhibitory activity with IC_50_ value of 9.19 µM, and high selectivity index, SI (TC_50_/IC_50_) 35.46 [320]. Ouyang et al. reported the synthesis and antiviral activities of pyrazole derivatives containing oxime moiety. The bioassay revealed that the compounds possessed antiviral activities. Compound **423** was found to possess inactivation effects against tobacco mosaic virus (TMV) (EC_50_ = 58.7 μg/mL) as the commercial product ningnanmycin (EC_50_ = 52.7 μg/mL) [321]. Jia et al. identified a series of novel pyrazole derivatives as non-nucleoside HBV inhibitors via bioisosterism and pharmacophore hybrid strategy. In particular, compound **424** displayed the most potent activity against the secretion of HBsAg and HBeAg with IC_50_ of 24.33 µM and 2.22 µM, respectively [322]. A series of pyrrolopyrazole derivatives were synthesized by Liu et al. and evaluated for their anti-viral activity against HIV-1. Among of them, compound **425** had potent anti-HIV-1 activities (EC_50_ = 3.98 µM) and excellent therapeutic index (TI, CC_50_/EC_50_ > 105.25). The compound has potential as lead compounds for further optimization into clinical anti-HIV-1 agents [323]. Novel pyrazole acyl thiourea derivatives were synthesized and tested for their anti-TMV activity. Amongst the new products compound **426** showed curative rates by 41.23% [78]. Pyrazolo[1,5-*a*]pyridine derivatives synthesized by Johns et al. were evaluated for antiviral activity against herpes virus. Compound **427** was reported as the most potent anti-viral agent with EC_50_ = 0.26 µM [324].

### 3.6. Anti-Azheimer’s Activity

**Figure 40:**
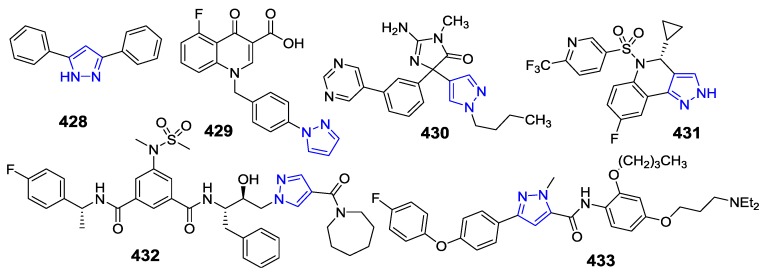
Pyrazole derivatives with anti-azheimer activity.

Chimenti et al. prepared a series of 3,5-diaryl pyrazoles (Figure 40) and assayed for their ability to inhibit reversibly monoamine oxidase-A (MAO-A) and monoamine oxidase B (MAO-B). Several compounds show inhibitory activity with concentration values in the nanomolar range. Compound **428** showed good inhibitory activity against MAO-A and MAO-B, but low selectivity (pIC_50_ MAO-A = 9.00 nM, pIC_50_ MAO-B = 8.00 nM, and pSI = 1.00) [325]. Kuduk et al. identified compound **429** as a potent and selective full agonist of the M_1_ positive allosteric modulators. Compound **429** also exhibited high potency, which gave an M1 IP = 94 nM and a high free fraction (10%) in rat and human plasma [326]. Malamas et al. developed new pyrazolyl and thienyl aminohydatoins as potent BACE1 inhibitors. The *n*-butyl analog **430** was the most potent analog, with an IC_50_ value of 8 nM [327]. A series of metabolically stable γ-secretase inhibitors selective for inhibition of the production of amyloid-β over notch were reported by Probst et al. Compound **431** both entered human clinical trials and lowered Aβ in the CSF of healthy human volunteers [328]. A series of pyrazole based compounds were synthesized by Zou et al. and identified them as novel C-terminus Beta-secretase 1 (BACE1) inhibitors. Further, modification over pyrazole scaffold lead to the identification of compound **432** as a potent inhibitor of BACE1 with IC_50_ value of 0.025 µM [329]. In an effort to develop novel inhibitors of receptor for advanced glycation end products (RAGE) for the treatment of Alzheimer’s disease, a series of pyrazole-5-carboxamides were synthesized by Han et al. and evaluated for anti-Alzheimer’s activity. Results indicated that the most active analogs **433** exhibited higher inhibitory activities and exhibited significant brain Aβ-lowering effects as well as favorable aqueous solubility [330].

**Figure 41:**
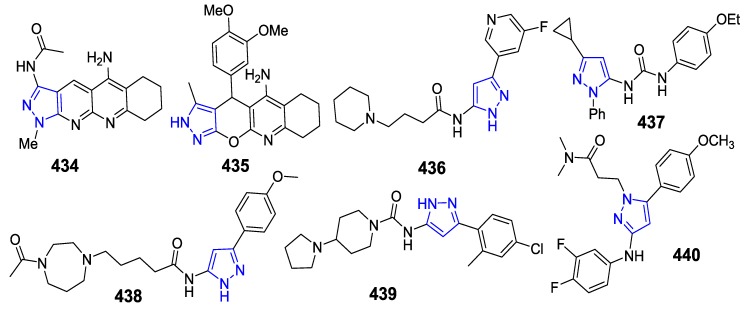
Pyrazole derivatives as anti-azheimer’s compounds.

A new series of pyrazolotacrine as acetylcholinesterase (AChE) inhibitors (Figure 41) were reported by Silva et al. The results showed that compound **434** was the most potent inhibitor of AChE, which inhibited the aforementioned enzyme with an IC_50_ value of 0.069 µM [331]. Khoobi et al. synthesized a new tetracyclic tacrine analogs containing pyrano[2,3-*c*]pyrazole and evaluated for inhibition of acetylcholinesterase (AChE). Compound **435** bearing 3,4-dimethoxyphenyl group was the most potent compound against AChE, being more active than the reference drug tacrine [332]. Zanaletti et al. developed a new α7 nicotinic acetylcholine receptors (α7 nAChR) represented promising therapeutic candidates for the treatment of cognitive impairment associated with Alzheimer’s disease (AD) and schizophrenia. Compound **436** was found a potent and selective full agonist of the α7 nAChR that demonstrated improved plasma stability, brain levels, and efficacy in behavioral cognition models [333]. The same author reported the synthesis and α7 nAChR inhibitory activity of novel class of pyrazole derivative. Compound **437** proved to be potent and selective, and it demonstrated a fair pharmacokinetic profile accompanied by efficacy in rodent behavioral cognition models [334]. Nencini et al. reported the design and synthesis of a pyrazole hybrid series of potent and selective agonists of α7 nicotinic acetylcholine receptor. The results revealed that compound **438** was the most potent inhibitor of α7 nAChR with an IC_50_ value of 0.07 µM [335]. AstraZeneca AB developed diverse series of pyrazole derivatives as positive allosteric modulators (PAMs). Compound **439** expressed good activity by inhibiting nicotinic acetylcholine receptors (*nAChRs*) [336]. Janssen Pharmaceutica identified a new series of trisubstituted pyrazole derivatives to PAM types 1–4 related to their kinetic properties in whole-cell voltage-clamp recordings using the agonist choline at a concentration of 1 mM. The trisubstituted pyrazole **440** showed remarkable activity with a pEC_50_ value of 7.11 (6268% efficacy) and a PAM type 4 profile [337].

### 3.7. Anti-Diabetic Activity

**Figure 42:**
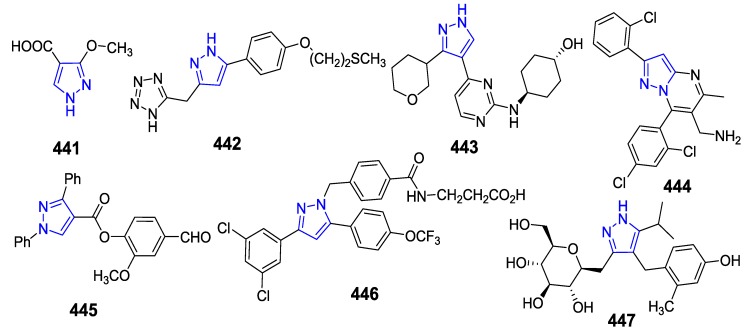
Pyrazole derivatives with anti-diabetic activity.

A new series of substituted pyrazole-4-carboxylic acids were synthesized (Figure 42) by Cottineau et al. and evaluated for their antidiabetic activity. The results indicated that compound **441** emerges as the best hypoglycemic agent in the series [338]. Sharon et al. synthesized a new series of 5-[(5-aryl-1*H*-pyrazol-3-yl)methyl]-1*H*-tetrazoles and evaluated them for their in vivo anti- hyperglycemic activity. Out of screened compounds, compound **442** demonstrated 24.6% of blood glucose lowering activity at 100 mg/kg [339]. Humphries et al. reported the development of a series of novel 4-pyrazolyl-2-aminopyrimidines as inhibitors of c-Jun *N*-terminal kinases. This study led to the identification of **443**, which showed good selectivity across a panel of diverse protein and lipid kinases [340]. Several pyrazolopyrimidines were synthesized by Brigance et al. and evaluated as inhibitors of dipeptidyl peptidase-4 (DPP4). Among the synthesized compounds, the **444** displayed the greatest potency (Ki = 20 nM) and demonstrated excellent selectivity over the other dipeptidyl peptidases [341]. Choi et al. identified 1,3-diphenyl-1*H*-pyrazole derivatives as a new series of potent *PPARγ* partial agonists using an improved virtual screening method combining ligand-centric and receptor-centric methods. The pyrazole-based compound **445** showed relatively strong binding activities against PPARγ among the virtual candidates [342]. A novel class of 1,3,5-pyrazoles has been discovered by Shen et al. as potent human glucagon receptor antagonists. Compound **446** was identified as a potent human glucagon receptor antagonist with good pharmacokinetic profiles in four preclinical species, and showed excellent oral pharmacodynamics efficacy in rhesus monkeys and transgenic mice by blocking glucagon-induced hyperglycemia [343]. A series of 4-benzyl-1*H*-pyrazol-3-yl β-d-glucopyranoside derivatives were synthesized and evaluated for their inhibitory activity toward sodium glucose co-transporter **1** (SGLTs). Compound **447** was identified as potent and selective SGLT1 inhibitors, both of which showed improved in vitro intestinal stability over phlorizin, high solubility to water [344].

**Figure 43:**
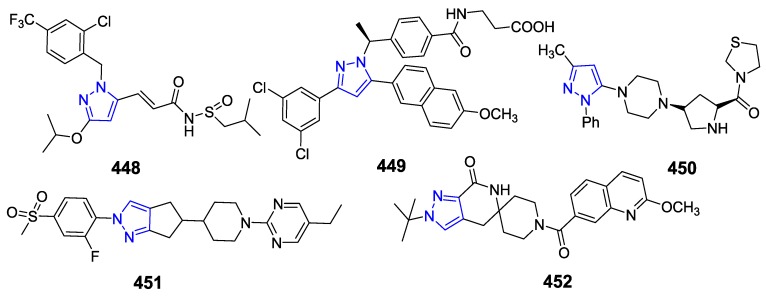
Pyrazole derivatives with anti-diabetic activity.

Rikimaru et al. described the design, synthesis, and structure-activity relationships of novel benzylpyrazole acylsulfonamides as non-thiazolidinedione, non-carboxylic-acid-based *peroxisome* proliferator activated receptor (PPAR) γ agonists (Figure 43). Overall, the compound **448** exhibited favorable metabolic stability and potent PPARγ agonist with EC_50_ values of 8.3 nM [345]. Xiong et al. discovered a new pyrazole compound **449** as a potent, selective glucagon receptor antagonist by optimization of a previously identified lead. Compound **449** is a reversible and competitive antagonist with high binding affinity (IC_50_ of 6.6 nM) and functional *cAMP* activity (IC_50_ of 15.7 nM) [346]. Yoshida et al. reported the discovery and preclinical profile of teneligliptin as a highly potent, selective, long-lasting and orally active dipeptidyl peptidase IV inhibitor for the treatment of type 2 diabetes. Compound **450** (teneligliptin), at 0.03 mg/kg or higher doses, significantly inhibited the increase of plasma glucose levels after an oral glucose load in Zucker fatty rats, and has been approved for the treatment of type 2 diabetes in Japan [347]. A series of pyrazole-based *GPR119* agonists were designed by Futatsugi et al., a novel and potent GPR119 full agonist **451** was identified through a conformational restriction-core flipping strategy. Compound **451** was roughly 10-fold less potent than exemplars from other series [348]. Griffith et al. disclosed a series of acetyl-CoA carboxylase (ACC) inhibitors based on a spirocyclic pyrazololactam core. Compound **452** showed excellent oral bioavailability, moderate systemic clearance, and acceptable exposure in rat pharmacokinetic studies. The oral dosing of rats with **452** resulted in potent, dose-proportional inhibition of ACC activity as measured by incorporation of ^14^C in DNL product [349].

**Figure 44:**
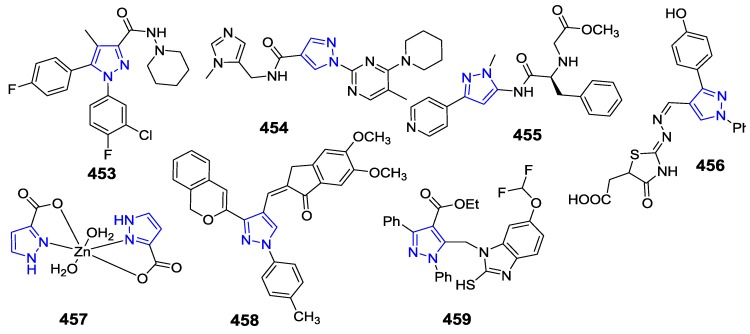
Pyrazole derivatives with anti-diabetic activity.

Novel 1,5-diaryl pyrazole derivatives were synthesized (Figure 44) by Hernández-Vázquez et al. and evaluated in vivo for their hypoglycemic activity. Compound **453** showed the most significant plasma glucose reduction, with decreases of 64% [350]. Toda et al. identified a new pyrazole compounds as novel insulin secretagogues for the treatment of type 2 diabetes. Compound **454** showed potent glucose lowering effects during an oral glucose tolerance test in mice and monkeys [351]. Yu et al. reported the lead optimization of pyrazole based GPR142 agonists. Structure−activity relationship studies indicated that amino-pyrazole-phenylalanine carboxylic acid **455**, exhibited good agonistic activity (h-GPR142-EC_50_ = 0.052 µM), high target selectivity, desirable pharmacokinetic properties, and no cytochrome P450 or hERG liability [352]. Bhosle et al. synthesized a new series of 2-hydrazolyl-4-thiazolidinone-5-carboxylic acids having pyrazolyl pharmacophores and evaluated their anti-hyperglycemic activity. Among the prepared compounds, **456** have displayed significant anti-hyperglycemic activity at 100 mg/kg [353]. A novel Zn mononuclear complex with 3-carboxy-pyrazole ligand has been prepared and evaluated in vivo for their antidiabetic activity. This compound **457** exhibits a potential in vivo anti-diabetic activity (62% reduction in blood glucose in the diabetic group treated compared with untreated diabetic) [354]. Kenchappa et al. reported the synthesis of coumarin derivatives containing pyrazole and indenone rings as potent anti-hyperglycemic agents. The compound **458** showed significant decrease in glucose concentration (115 and 138 mg/dL) with the dose of 100 mg/kg [355]. A series of substituted pyrazoles derivatives were synthesized by Doddaramappa et al. and tested in vitro for their anti-diabetic activity by measuring the α-amylase and α-glucosidase inhibitory potential. Among the synthesized compounds, compound **459** emerged as an excellent antidiabetic agent with IC_50_ values of 10 and 15 µg/mL against α-amylase and α-glucosidase inhibitors, respectively [356]. 

**Figure 45:**
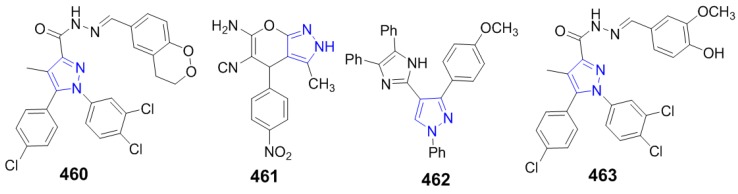
Pyrazole derivatives with anti-diabetic activity.

Hernández-Vázquez et al. reported the design, synthesis and anti-diabetic activity of novel *N*′-arylidene pyrazole-3-carbohydrazides (Figure 45). Compound **460** exhibited a remarkable hypoglycemic effect with a 90% of plasma glucose reduction [357]. A series of dihydropyrano[2,3-*c*] pyrazoles derivatives were synthesized and evaluated for their α-glucosidase inhibitory activity. Compound **461** was the most potent analog of this series (IC_50_ = 54.2 µM), when compared with standard drug, i.e., acarbose (IC_50_ = 937.0 µM) [358]. A series of imidazolylpyrazoles were synthesized by Chaudhry et al. and tested for their α-glucosidase inhibitory activity. The in vitro enzyme inhibition indicated that the compound **462** showed significant inhibitory potentials and binding affinities (IC_50_ = 23.95 µM) as compared to that of reference acarbose [359]. During current investigation concerning the synthesis of novel 1,5-diarylpyrazole derivatives as antidiabetic entities, Hernández-Vázquez et al. synthesized the hybrid **463**, a novel dual compound that exhibited both anti-diabetic and in vitro antioxidant effects. Compound **463** showed a pronounced anti-hyperglycemic effect even at a dose of 5 mg/kg (*p* < 0.001) in a glucose tolerance test on normoglycemic rats [360].

### 3.8. Anti-Leishmanial Activity

**Figure 46:**
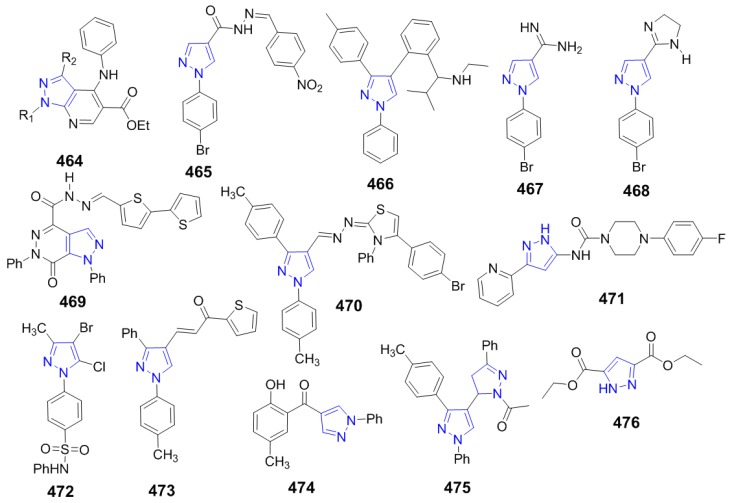
Pyrazole derivatives with anti-leishmanial activity.

A series of 4-anilino-1*H*-pyrazolo[3,4-*b*]pyridine-5-carboxylic esters (Figure 46) were synthesized by De Mello et al. and tested against promastigote forms of *Leishmania amazonensis* as part of a program to study potential anti-Leishmania drugs. The very promising results showed the compound **464** as the most active [IC_50_ = 0.12 μM (22)] [361]. Bernardino et al. reported the synthesis and in vitro leishmanicidal acitivities of 1*H*-pyrazole-4-carbohydrazides. Among all the 1*H*-pyrazole-4-carbohydrazides derivatives examined, the compound **465** was found the most active against *L. amazonensis*, *L. chagasi* and *L. braziliensis* species [362]. Dardari et al. reported the synthesis and the antileishmanial activity of a new pyrazole derivative **466**. This compound inhibited the in vitro multiplication of *Leishmania tropica*, *Leishmania major*, and *Leishmania infantum* with IC_50_ values of 0.48 μg/mL, 0.63 μg/mL and 0.40 μg/mL, respectively [363]. 1-Aryl-1*H*-pyrazole- 4-carboximidamide derivatives were synthesized by Dos Santos et al. and evaluated in vitro for their anti-leishmanial activities. Compound **467** showed an activity profile that can be improved through medicinal chemistry strategies [364]. This same author, synthesized a series of 1-aryl-4-(4,5-dihydro-1*H*-imidazol-2-yl)-1*H*-pyrazoles and evaluated in vitro against three Leishmania species: *L. amazonensis*, *L. braziliensis* and *L. infantum* (*L. chagasi syn*.). Among the derivatives examined, compound **468** emerged as the most active on promastigotes forms of *L. amazonensis*, with an IC_50_ value of 15 μM [365]. A new series of pyrazolo[3,4-*d*]pyridazin-7-one derivatives were synthesised and evaluated for their *in vitro* anti-leishmanial activity against *Leishmania amazonensis* promastigote and axenic amastigote forms. The results showed that compound **469** exhibit better anti-leishmanial activity with IC_50_ 3.63 and 2.32 μM, against the *promastigote* form and axenic amastigote form, respectively [366]. 

A new series of pyrazole derivatives were synthesized by Bekhit et al. and evaluated for their in vitro anti-leishmanial activity against Leishmania aethiopica promastigotes and amastigotes. The results showed that compound **470** had the highest anti-leishmanial activity, with an IC_50_ value of [367]. Mowbray et al. identified a novel series of amino-pyrazole ureas with potent in vitro anti-leishmanial activity. Furthermore, compound **471** showed high levels of in vivo efficacy (>90%) against *Leishmania infantum*, thus demonstrating proof of concept for this series [368]. A series of 4-(1*H*-pyrazol-1-yl)-benzenesulfonamides were synthesized and evaluated in vitro for their anti-leishmanial profile against *Leishmania infantum* and *Leishmania amazonensis*. Interestingly, **472** showed the best in vitro active profile against the infective *L. amazonensis* promastigotes and *L. infantum* forms, with IC_50_ values of 0.059 and 0.070 mM, respectively [369]. Bekhit et al. prepared a novel series of 1*H*-pyrazole derivatives and tested for their in vitro anti-leishmanial activities against *L. aethiopica* promastigotes. The highest anti-leishmanial activity was exhibited by compound **473**, with an IC_50_ of 0.079 µg/mL [370]. Pyrazole derivatives were prepared and tested for their in vitro antiparasitic activity against promastigotes of *Leishmania mexicana* (Bel 21) and epimastigotes of *Trypanosoma cruzi* (DM28) using a modified MTT assay. Only **474** displayed selectivity on *L. mexicana* with a SI of 3, however, the IC_50_ obtained here was around four times higher (25 μM) [371]. A new series of pyrazole derivatives were prepared and tested in vitro for their anti-leishmanial activity. Compound **475** was found to be the most active (IC_50_ = 0.0112 µg/mL) than the standards miltefosine (IC_50_ = 0.3 ± 0.04 µg/mL) and amphotericin B deoxycholate (IC_50_ = 0.2 ± 0.02 µg/mL) for *Leishmania donovani* [372]. Reviriego et al. reported the synthesis and antiprotozoal activity of some simple dialkyl pyrazole-3,5-dicarboxylates against *Trypanosoma cruzi*, *Leishmania infantum* and *Leishmania braziliensis*. The diethyl ester **476** showed high efficiency against the mentioned protozoa [373].

### 3.9. Anti-Malarial Activity

**Figure 47:**
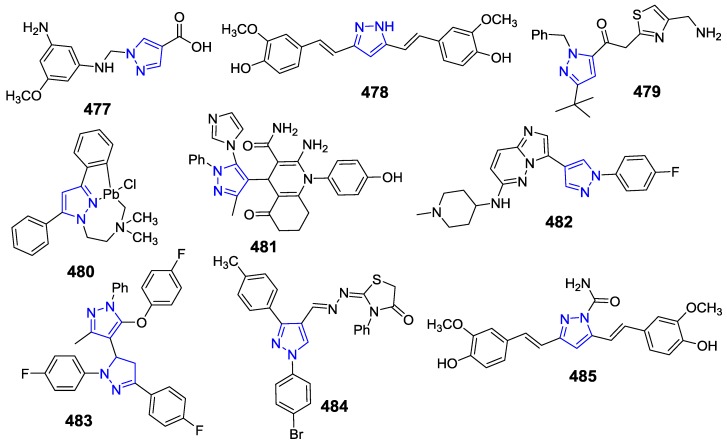
Pyrazole derivatives with anti-malarial activity.

A series of pyrazoles were described (Figure 47) as part of efforts directed toward the synthesis of some potent antimalarial agents. Further modification of the heterocyclic ring to give substituted aryl derivatives afforded potent antimalarial derivatives **477** with IC_50_ = 0.149 µmol/L [374]. Novel curcumin analogues bearing pyrazole ring were prepared and evaluated for their anti-malarial activity against *CQ-S* and *CQ-R Plasmodium falciparum* culture. Compound **478** was found to be the most potent analogue with IC_50_ values 0.48 and 0.45 μM against *CQ-S* and *CQ-R*, respectively [375]. Gonzalez Cabrera et al. identified an aminomethylthiazole pyrazole carboxamide lead **479** with good in vitro antiplasmodial activity [IC_50_: 0.08 μM (*K1*, chloroquine and multidrug resistant strain) and 0.07 μM (NF54, chloroquine-sensitive strain)] and microsomal metabolic stability from whole cell screening of a SoftFocus kinase library. Compound **479** also exhibited in vivo activity in the *P. berghei* mouse model at 4 × 50 mg/kg administration via the oral route, showing 99.5% activity [376]. Quirante et al. described the synthesis and in vitro antimalarial activities of platinum(II) and palladium(II) complexes with ligands derived from pyrazole. Compound **480** exhibited only moderate antimalarial activities against two *P. falciparum* strains (3D7 and W2) [377]. A new category of polyhydroquinoline derivatives containing pyrazole moieties were prepared by Kalaria et al. and evaluated for their *in vitro* antimalarial activity against *Plasmodium falciparum*. Among the tested compounds, compound **481** exhibited excellent antimalarial activity with an IC_50_ value of 0.033 µg/mL [261]. A series of imidazopyridazine inhibitors of *Plasmodium falciparum* calcium-dependent protein kinase 1 (PfCDPK1) has been explored and extended by Large et al. Diaminocyclohexane **482** showed a good in vitro potency and metabolic stability profile against *Plasmodium falciparum* (PfCDPK1 enzyme IC_50_ = 0.056 µM, Pf anti-parasite EC_50_ = 0.262 µM) [378]. A novel series of fluoro-substituted pyrazolylpyrazolines were synthesized and screened for their antimalarial activity against *Plasmodium falciparum*. Compound **483** displayed excellent activity with an IC_50_ value of 0.022 µg/mL against *P. falciparum* stain as compared to quinine IC_50_ = 0.268 µg/mL [262]. A new series of pyrazole derivatives were synthesized and evaluated for their in vivo antimalarial activity against *Plasmodium berghei*-infected mice and the most active derivatives were further examined for their in vitro antimalarial activity against chloroquine resistant (RKL9) strain of *Plasmodium falciparum*. Compound **484** had more than 90% parasite suppression activity of that found with the antimalarial reference standard drug, chloroquine phosphate and had lower IC_50_ values than chloroquine [367]. Belaji et al. reported the molecular modelling, synthesis, and antimalarial potentials of curcumin analogues containing pyrazole ring. The compound **485** showed the most significant result, with maximum schizonticidal (IC_50_ = 1.48 μM) and parasiticidal activities (MKC; 3.87 = μM) [379]. 

### 3.10. Anti-Parkinson Activity

**Figure 48:**
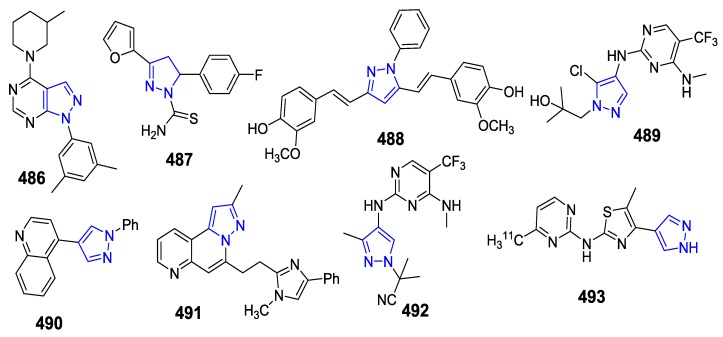
Pyrazole derivatives with anti-parkinson activity.

Niswender et al. discovered a new pyrazolo[3,4-*d*]pyrimidines derivatives (Figure 48) as novel modulators of the metabotropic glutamate receptor subtype 4 (mGluR4) positive allosteric modulators. Results indicated that the compound **486** showed a remarkable anti-parkinson activity [380]. A series of *N*1-thiocarbamoyl-3,5-di(hetero)aryl-4,5-dihydro-(1*H*)-pyrazole derivatives has been synthesized by Chimenti et al. and tested for their ability to inhibit the activity of the A and B isoforms of human monoamine oxidase (hMAO). Compound **487** was found the most active of the series with IC_50_ value of 2.75 µM and selectivity ratio of 25 [381]. Maher et al. synthesized a pyrazole structure **488** as aderivative of curcumin and was tested for its ability to enhance the activity of Ca^2+^/calmodulin dependent protein kinase II (CaMKII). Results indicated that the compound **488** enhanced the induction both long-term potentiation (LTP) in rat hippocampal slices and memory in a rat object [382]. Chan et al. identified a new aminopyrazole as a Leucine-Rich Repeat Kinase 2 (LRRK2) inhibitor. In in vivo rodent PKPD studies, compound **489** demonstrated good brain exposure and engendered significant reduction in brain pLRRK2 levels post-ip administration [383]. 4-(1-Phenyl-1*H*-pyrazol-4-yl)quinoline **490** was identified by screening the Lundbeck compound collection, and characterized as having mGlu4 receptor positive allosteric modulator properties. Compound **490** showed excellent anti-parkinson activity with an EC_50_ value of 220 nM [384]. Dore et al. designed and synthesized a novel tricyclic pyrazoles as potent phosphodiesterase 10A (*PDE10A*). Pyrazolo[5,1-*f*] [1,6]naphthyridine **491** showed the highest affinity for PDE10A enzyme (IC_50_ = 40 nM) [385]. Estrada et al. discovered a new aminopyrazoles as Leucine-Rich Repeat Kinase 2 (LRRK2) inhibitors. Compound **492** was identified as as highly potent and selective LRRK2 inhibitors with IC_50_ value of 3 nM [386]. Fujinaga et al. reported the radio-synthesis and evaluation of 5-methyl-*N*-(4-[^11^C]methylpyrimidin-2-yl)-4-(1*H*-pyrazol-4-yl)thiazol-2-amine (493) as a novel radio-ligand for imaging of metabotropic glutamate receptor subtype 4 (mGluR4). In vitro autoradiography and ex vivo bio-distribution study in rat brains showed specific binding of compound **493** in the cerebellum, striatum, thalamus, cerebral cortex, and medulla oblongata, which showed dose-dependent decreases by administration with multiple dosing [387].

### 3.11. Agrochemical Activity

**Figure 49:**
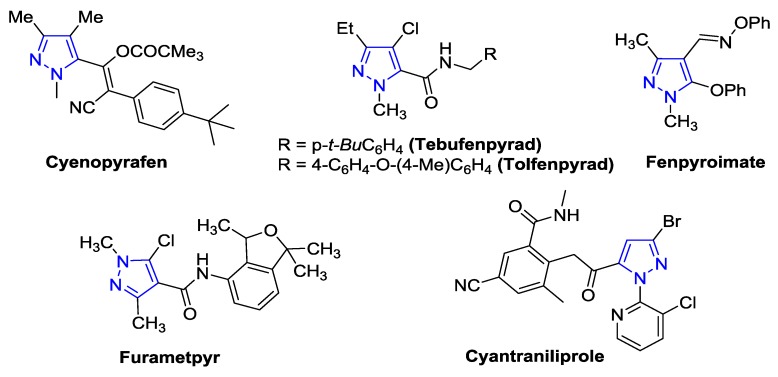
Agrochemical molecules containning pyrazole scaffold.

In the past few years, the interest in pyrazole derivatives has increased due to their proven usefulness as intermediates in the preparation of new biological materials. Specifically, pyrazole derivatives have a long history of application in the agrochemical industry as herbicides, insecticides, fungicides and acaracides. The pyrazole ring is present in many agrochemically important compounds, such as the pesticides, furametpyr [388], cyantraniliprole [389], cyenopyrafen [390], tebufenpyrad [391], tolfenpyrad [392], and fenpyroximate [388] (Figure 49).

**Figure 50:**
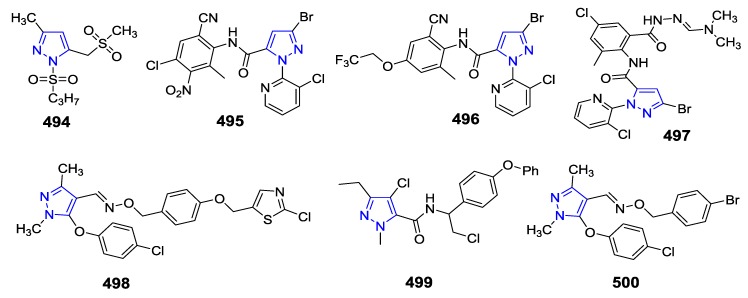
Pyrazole derivatives with insecticidal and acaricidal activities.

Finkelstein et al. described the synthesis and insecticidal activity of novel pyrazole methanesulfonates (Figure 50) against *Diabrotica undecimpunctata Howardi*, *Nilaparvata lugens*, *Nephotettix cincticeps*. Compound **494** showed a very high level of activity with LD values of 0.5, 2.5 and 5.5 mg/L against *Diabrotica undecimpunctata Howardi*, *Nilaparvata lugens*, *Nephotettix cincticeps*, *r*espectively [393]. A series of novel *N*-pyridylpyrazolecarboxamides were designed and synthesized by Mao et al. The bioassays showed that some of the compounds exhibited excellent insecticidal activities against oriental armyworm (*Mythimna separata*) and diamondback moth (*Plutella xylostella*). Compound **495** showed 86% larvicidal activities against *P. xylostella* at the concentration of 0.1 mg/L, while the activity of compound **496** against *M. separata* was 80% at 1 mg/L [394]. Wu et al. reported the synthesis and insecticidal activities of novel pyrazole amide derivatives containing hydrazone substructures. In vivo tests indicated that the compound **497** exhibited good activity against different insect species, such as *P. xylostella*, *H. armigera*, *C. pipiens pallens*, *N. lugens* and *R. maidis* [395]. A series of new pyrazole oximes bearing substituted thiazole ring were prepared and tested for their insecticidal and acaricidal activities. The results of primary bioassay indicated that the compound **498** was more potent against *Tetranychus cinnabarinus* and *Plutella xylostella* than other analogues [396]. Song et al. synthesized a novel pyrazole derivatives and evaluated for their insecticidal activity against cotton bollworm (*Helicoverpa armigera*), diamondback moth (*Plutella xylostella*), bean aphid (*Aphis craccivora*), mosquito (*Culex pipiens pallens*), and spider mite (*Tetranychus cinnabarinus*). The results of bioassays indicated that the compound **499** showed high insecticidal activity against cotton bollworm was 60% at 5 mg/kg [397]. Fu et al. reported the synthesis and insecticidal activities of novel pyrazole oxime ether derivatives. Bioassays showed that at a 10 mg/L, the insecticidal activity of compounds **500** exceeded 90% [398].

**Figure 51:**
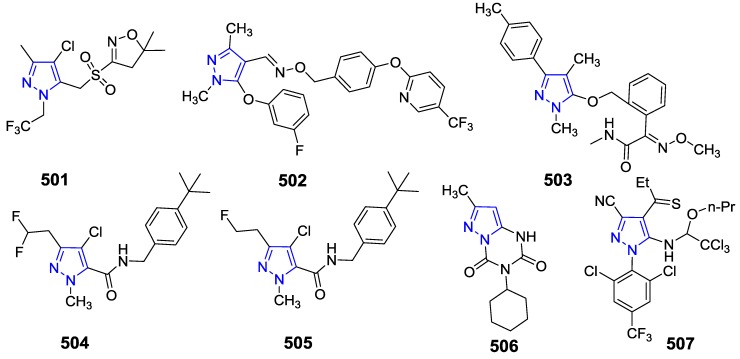
Pyrazole derivatives with pesticidal activity.

A series of novel *N*-(2,2,2)-trifluoroethylpyrazole derivatives (Figure 51) were synthesized and tested for their herbicidal activity. The bioassay results indicated that the compound **501** showed the best preemergence herbicidal effects against both dicotyledonous and monocotyledonous weeds with good safety to maize and rape at the dosage of 150 g·ha^−1^ in greenhouse [399]. Dai et al. synthesized a new series of pyrazole oxime derivatives containing a 5-trifluoromethylpyridyl moiety and evaluated for their insecticidal and acaricidal activities against *Plutella xylostella*, *Aphis craccivora* and *Tetranychus cinnabarinus.* Results showed that the compound **502** possessed excellent acaricidal activity against *T. cinnabarinus* and displayed potential insecticidal activity against *P. xylostella* and *A. craccivora* [400]. Li et al. reported Synthesis and fungicidal activities of new pyrazole derivatives. The test results indicated that the compound **503** exhibited strong fungicidal activities against *Pyricularia oryzae*, *Phytophthora infestans*, *Pseudoperonospora cubensis*, and *Erysiphe graminis* [401]. Fustero et al. described the synthesis of new fluorinated tebufenpyrad analogs with acaricidal activity. Among the synthesized compounds, two of these compounds **504** and **505** display a fertility inhibition superior to that of tebufenpyrad [402]. Two series of new pyrazoles, namely pyrazolo[1,5-*a*][1,3,5]triazine-2,4-dione and pyrazolo-[1,5-*c*][1,3,5]thiadiazine-2-one derivatives, were synthesized as potential inhibitors of the photosynthetic electron transport chain at the photosystem II level. Among the pyrazolo[1,5-*a*][1,3,5]triazine-2,4-dione derivatives, those including cyclohexyl substituents like **506** showed maximal activity [403]. A series of novel phenylpyrazoles containing a 2,2,2-trichloro-1-alkoxyethyl moiety were designed and synthesized by Zhao et al. and tested for their insecticidal activity. The results of bioassays indicated that the target compounds possessed excellent activities against a broad spectrum of insects such as bean aphid (*Aphis craccivora*), mosquito (*Culex pipiens pallens*) and diamondback moth (*Plutella xylostella*). Especially, the foliar contact activity against bean aphid of compound **507** at 2.5 mg/kg was 89% [404].

## 4. Conclusions

Pyrazoles represent a major pharmacophore with various biological properties, and some pyrazole-containing derivatives have already been used for therapeutic purposes. This literature review shows that pyrazole derivatives are pharmacologically very potent and, therefore, their design and synthesis is the potential area of research. It has been noted so far that the structural modifications of the basic structure of pyrazole, have allowed the preparation of new derivatives with a broad spectrum of biological activity, with the most important structural variations concerning the substituents at the 1-position, the carbon at the 3-position and the substituent at the 5-position. Previous studies have shown that the structural modification on the different positions of the basic molecule allows for improving its pharmacological profile, giving it antimicrobial, anticonvulsant, analgesic, anti-inflammatory, anti-viral, anti-malarial and anti-cancer properties. For the moment, researchers have been drawn to the design of more potent pyrazole derivatives having great diversity of biological activity.

## Figures and Tables

**Table 1:** Preparation of pyrazoles **55** from β-aminoenones **49a**, **49b** and **49c**.

Starting Compound	R	R1	Yields of 51 (%)
**50a**	(CH_2_)_2_Ph	Me	81
	(CH_2_)_2_Ph	t-Bu	78
**50b**	iPr	Me	84
	iPr	t-Bu	86
**50c**	t-Bu	Me	72
	t-Bu	t-Bu	86

**Table 2:** Preparation of pyrazoles **54** from β-aminoenones **54d**, **54e** and **54f**.

Starting Compound	R	R1	Yields of 54 (%)	Ratio 54:53
**50d**	(CH_2_)_2_Ph	Me	81	1:0
	(CH_2_)_2_Ph	t-Bu	23	1:1.4
**50e**	iPr	Me	71	1:0
	iPr	t-Bu	-	1:9
**50f**	t-Bu	Me	72	11:1
